# Discovery and characterisation of an amidine-containing ribosomally-synthesised peptide that is widely distributed in nature†

**DOI:** 10.1039/d1sc01456k

**Published:** 2021-08-02

**Authors:** Alicia H. Russell, Natalia M. Vior, Edward S. Hems, Rodney Lacret, Andrew W. Truman

**Affiliations:** Department of Molecular Microbiology, John Innes Centre Norwich NR4 7UH UK andrew.truman@jic.ac.uk

## Abstract

Ribosomally synthesised and post-translationally modified peptides (RiPPs) are a structurally diverse class of natural product with a wide range of bioactivities. Genome mining for RiPP biosynthetic gene clusters (BGCs) is often hampered by poor annotation of the short precursor peptides that are ultimately modified into the final molecule. Here, we utilise a previously described genome mining tool, RiPPER, to identify novel RiPP precursor peptides near YcaO-domain proteins, enzymes that catalyse various RiPP post-translational modifications including heterocyclisation and thioamidation. Using this dataset, we identified a novel and diverse family of RiPP BGCs spanning over 230 species of Actinobacteria and Firmicutes. A representative BGC from *Streptomyces albidoflavus* J1074 (formerly known as *Streptomyces albus*) was characterised, leading to the discovery of streptamidine, a novel amidine-containing RiPP. This new BGC family highlights the breadth of unexplored natural products with structurally rare features, even in model organisms.

## Abstract 2

Genome mining for pathways containing YcaO proteins revealed a widespread novel family of RiPP gene clusters. A model gene cluster was characterised through genetic and chemical analyses, which yielded streptamidine, a novel amidine-containing RiPP.

## Graphical abstract

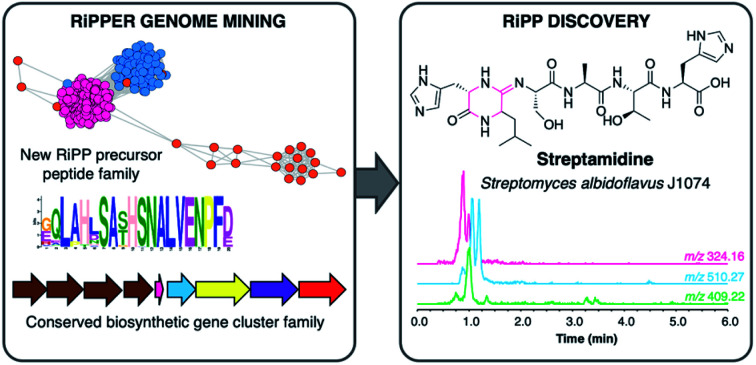

## Introduction

Microorganisms produce an array of natural products (NPs) with diverse and important biological activities.^1^ The phylum Actinobacteria is a particularly prominent source of NPs that have been utilised as antimicrobial drugs.^2^ It has been widely shown that bacteria are capable of producing many more NPs than are currently known, due to the abundance of uncharacterised biosynthetic gene clusters (BGCs) present in microbial genomes.^3,4^ Ribosomally synthesised and post-translationally modified peptides (RiPPs) are a large and growing class of structurally diverse NPs.^5^ RiPPs are produced from a ribosomally synthesised precursor peptide that is typically comprised of a leader region and a core region; some precursors also feature a follower peptide in addition to, or instead of, the leader peptide.^6^ Post-translational modifications are installed onto the core region of the precursor peptide by a series of RiPP tailoring enzymes (RTEs), which introduce structural diversity and complexity.^7,8^ The leader peptide is usually proteolytically removed as a late-stage step in RiPP biosynthesis.

Whilst genome mining is a popular approach to identify uncharacterised BGCs, the identification of novel RiPP BGCs is particularly challenging because the small precursor peptides that are ultimately transformed into the final product are often not annotated in genomes. Also, unlike with other natural product classes such as polyketides and non-ribosomal peptides, the short biosynthetic pathways for RiPPs lack universally shared features.^9^ Specific genome mining tools for RiPPs have been developed,^10–16^ but many of these tools rely on the identification of homology to known RiPP classes. Therefore, the opportunity to identify novel RiPP precursor peptides, and subsequent untapped structural complexity, might be missed. In the last two decades, hundreds of thousands of bacterial genomes have been sequenced, but their biosynthetic capacities have not been fully explored. The use of more bespoke genome mining tools therefore represents an important opportunity to identify cryptic and uncharacterised BGCs.

**Fig. 1:**
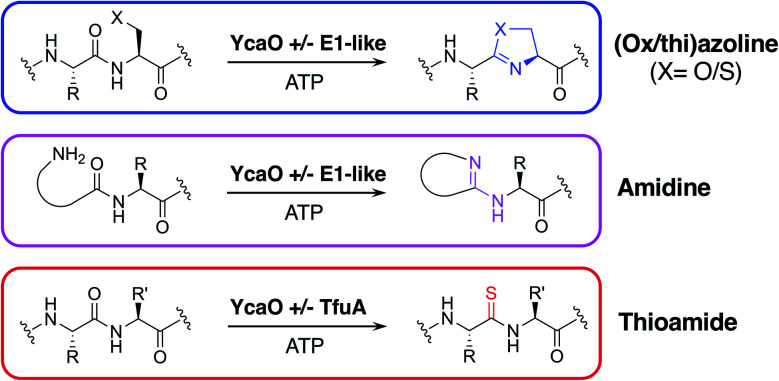
Reactions catalysed by YcaO-domain proteins.

One of the most widespread families of proteins associated with RiPP biosynthesis are YcaO-domain proteins, which are ATP-dependent enzymes found in both bacteria and archaea,^17,18^ and have been shown to catalyse various post-translational modifications of RiPPs (Fig. 1). These modifications include the installation of oxazoline and thiazoline heterocycles onto the precursor peptide backbone, where cyclodehydration is catalysed by the YcaO-domain in cooperation with a protein homologous to an E1 ubiquitin-activation enzyme or an “Ocin-ThiF-like” protein.^19,20^ YcaO proteins have also been demonstrated to catalyse the formation of amidine rings in bottromycin^21,22^ and klebsazolicin,^23^ and can also function with a TfuA-domain protein to introduce thioamide bonds into RiPPs such as thiopeptin^24^ and the thioamitides,^9,25^ and archaeal methyl-coenzyme M reductase.^26^ Over 15 000 proteins are annotated with YcaO domains (UniProtKB), but the function of the majority of these remains unknown.^18^ The diversity of YcaO-domain catalysed modifications means that associated precursor peptides can greatly vary in amino acid sequence.

We have previously reported a RiPP genome mining tool, RiPPER^9,27^ (RiPP Precursor Peptide Enhanced Recognition), which identifies precursor peptides without the need for information about RiPP structural class. RiPPER captures surrounding DNA regions of putative RTEs and searches for short open reading frames that might encode RiPP precursor peptides. In this study, we use RiPPER to identify precursor peptides encoded near all standalone YcaO-domain proteins in Actinobacteria. This analysis identified a large family of novel and diverse RiPP BGCs that span over 230 bacterial species. The sequence variation of both the identified precursor peptides, as well as the associated RTEs, suggests that these BGCs produce structurally distinct molecules with variable post-translational modifications. We characterised an exemplar of this new RiPP BGC family from the model actinobacterium, *Streptomyces albidoflavus* J1074 (formerly known as *Streptomyces albus* J1074 (ref. 28)), which led to the discovery of streptamidine, a novel and structurally rare amidine-containing molecule. The prevalence of this RiPP family highlights that we are still scratching the surface of the huge biosynthetic capabilities of microorganisms.

## Results and discussion

### Identification of novel RiPP precursor peptides

**Fig. 2:**
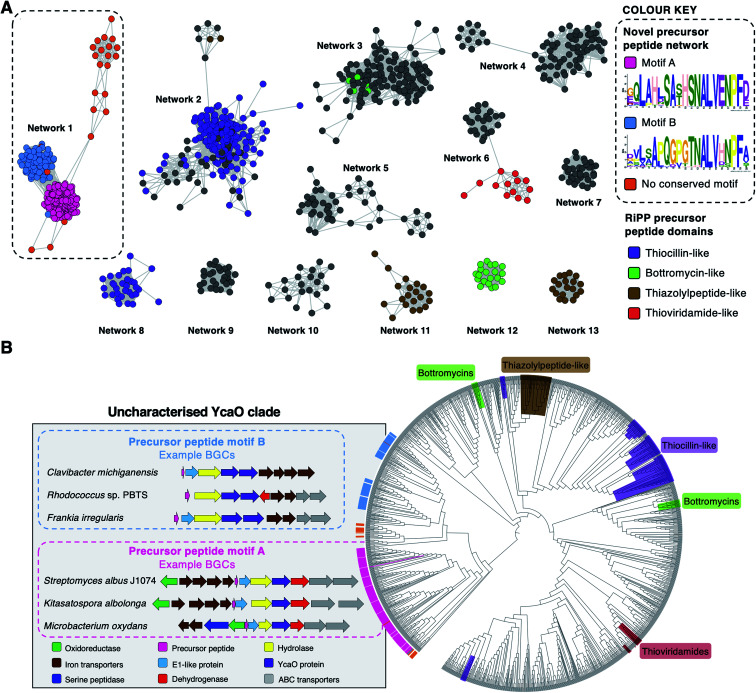
Bioinformatic identification of a new family of RiPPs. (A) Sequence similarity networking of short peptides identified by RiPPER analysis of actinobacterial YcaO proteins. Peptides with homology to known RiPP classes are highlighted. Network 1 is the focus of this study, which is sub-divided based on the presence of distinct sequence motifs. The full peptide networking output is shown in Fig. S1 and ESI dataset 1.† (B) Phylogenetic tree^32^ of all standalone actinobacterial YcaO-domain proteins (a tree with branch distances is shown in Fig. S2†). The novel clades of YcaO-domain proteins identified from this analysis are highlighted according to their associated precursor peptide in pink (motif A), blue (motif B) and orange (no conserved motif). BGC examples are shown. YcaO proteins associated with precursor peptides with known RiPP HMM domains^33^ from NCBI are also highlighted: red (thioviridamide family, NF033415), green (bottromycin family, NF033414), brown (thiazolylpeptide family, NF033400), and purple (thiocillin-like families, NF033482 and NF033401).

To investigate the diversity of YcaO-associated RiPP pathways, we focussed on standalone YcaO-domain proteins (*i.e.* those not fused to an additional domain) encoded in actinobacterial genomes, as the function of most of these standalone YcaO proteins are unknown^18^ and, unlike in archaea^29^ or the phylum Proteobacteria,^30^ there is no evidence that YcaO proteins are involved in non-RiPP modifications in Actinobacteria. 2574 proteins were retrieved from GenBank, which were further filtered to 1514 using a 95% maximum identity cut-off.^31^ Using these YcaO proteins as bait, RiPPER was used to retrieve associated short peptides and group them into families using similarity networking (40% minimum identity cut-off). This analysis revealed a series of peptide families encoded within 8 kb of the *ycaO* genes (Fig. S1, ESI datasets 1 and 2†). As expected, these families included precursors to known YcaO-modified RiPP families, including the bottromycins, thioviridamide-like molecules and thiopeptides (Fig. 2A). However, the most abundant peptide family (“Network 1”) consisted of 231 peptides whose RiPP products were completely unknown and only 78 were originally annotated as genes.

To determine whether this precursor peptide family was present in other phyla, further analyses using BLAST^34^ and RiPPER were carried out. This revealed that six bacteria in the phylum Firmicutes also encoded related peptides near YcaO proteins (ESI dataset 2†). Overall, 237 network 1 precursor peptides were identified by RiPPER, and were present in eight orders, 22 bacterial families and 57 different genera (ESI dataset 3†). The identified precursor peptides varied in length between 31 and 89 residues, highlighting their diversity. A MEME analysis^35^ of the peptides identified two distinct sequence motifs (A and B, Fig. 2A), either of which appeared once, twice or three times in each precursor peptide (Fig. S3 and S4†). Whilst these motifs differ greatly in sequence, three consecutive residues (ALV) are conserved between the two motifs. In addition, 22 sequences lacked motifs A or B, and might therefore represent further precursor peptide diversification within this family. Notably, none of these peptides have serine/threonine/cysteine-rich regions that are characteristic of many precursor peptides modified by YcaO proteins.^18^ NeuRiPP, a machine learning algorithm for the detection of precursor peptides,^36^ was unable to recognise the majority (91%) of network 1 peptides as RiPP precursor peptides (ESI dataset 3†), which highlights their sequence novelty in relation to known RiPPs.

To investigate the relationship between putative precursor peptide sequence and YcaO-domain protein, these peptides were mapped to a phylogenetic tree of all actinobacterial standalone YcaO proteins (Fig. 2B). This mapping clearly showed that this putative new family of precursor peptides is associated with a single clade of phylogenetically related YcaO-domain proteins. There are also distinct sub-clades that clearly associate with precursor peptides containing either motif A or motif B. A further similarity networking analysis of these 237 peptides using an 80% minimum identity cut-off resulted in a series of sub-families that mainly group by bacterial phylogeny (Fig. S5†). These sub-families again map tightly to YcaO protein phylogeny (Fig. S6†).

### Genetic organisation of newly discovered BGCs

The genes accompanying the YcaO and precursor peptide genes in this new family of RiPPs also show a high degree of conservation. MultiGeneBlast^37^ analysis of the newly identified BGCs revealed several subsets of BGCs whose genetic organisation correlates with the subclades identified within the family (Fig. 2B and S7†). The major one, found in over 90 BGCs (Fig. S7A†) contains the following set of conserved genes: four iron transporter genes with homology to the FecBCDE system^38^ (*amiF1–F4*), the putative precursor peptide (*amiA*), a conserved hypothetical protein (*amiB*), a hydrolase (*amiC*), the YcaO-domain protein (*amiD*), a flavin-dependent dehydrogenase (*amiE*) and two ABC transporters (*amiT1* and *amiT2*). This subset of BGCs is usually associated with precursor peptides containing motif A (Fig. 2B). An exemplar of this BGC is found in the model streptomycete, *S. albidoflavus* J1074.^39^ This BGC also features a partially conserved hypothetical gene upstream of the iron transporters (*amiX*), which could also form part of the BGC. Other BGCs associated with the identified peptides have further diversity in their genetic composition. For example, many of the BGCs lack homologues of the *amiB*, *amiX* and *amiE* (dehydrogenase) genes, or contain additional hypothetical proteins with no identifiable conserved domains (Fig. S7B†). Within these BGCs, a subset found primarily in *Frankia*, *Rhodococcus* and *Clavibacter* each encode two YcaO-domain proteins and are usually associated with precursor peptides containing motif B (Fig. 2B and S7B†).

### Heterologous expression of the *S. albidoflavus* BGC

**Fig. 3:**
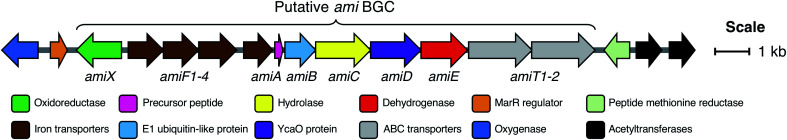
The 18.5 kb region of the *S. albidoflavus* J1074 genome cloned into pCAP03 to generate pCAPSalbC. This region contains the putative full ami BCG (marked with a bracket) and additional flanking genes.

The BGC from *S. albidoflavus* J1074 was selected as a model for characterisation, as this contained the most widespread precursor peptide motif and BGC architecture. The resulting natural product would therefore represent the most abundant RiPP produced by the identified BGCs. We used transformation-associated recombination (TAR) cloning^40,41^ in yeast to capture an 18.5 kb region of genomic DNA from *S. albidoflavus* J1074 (full region shown in Fig. 3) and generate plasmid pCAPSalbC. This region contained the putative BGC, as well as additional upstream and downstream genes that could feasibly have biosynthetic roles, including genes encoding an oxygenase, a MarR transcriptional regulator, a peptide methionine sulfoxide reductase, and two acetyltransferases.

**Fig. 4:**
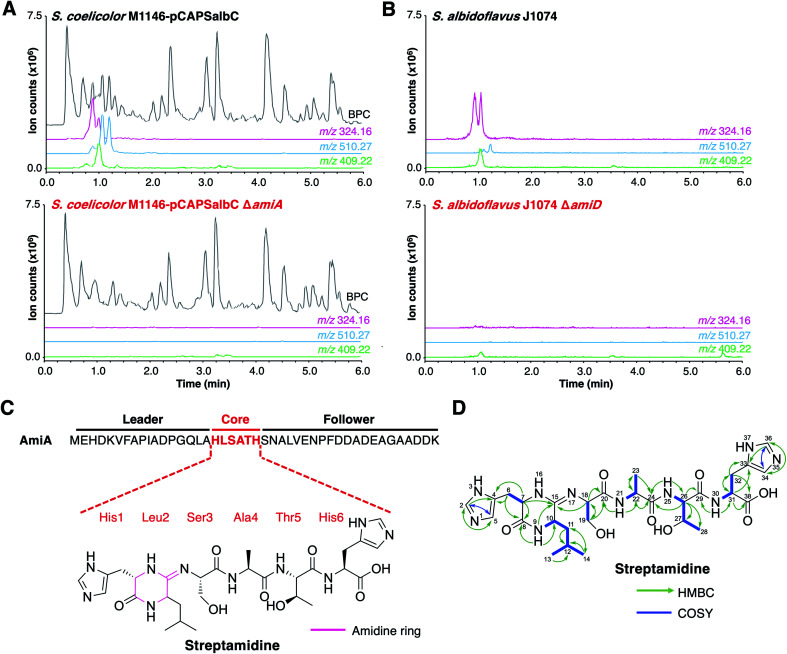
Discovery of streptamidine. (A) LC-MS chromatograms of *S. coelicolor* M1146-pCAPSalbC compared with *S. coelicolor* M1146-pCAPSalbC Δ*amiA*. Extracted ion chromatograms of three BGC-associated compounds are shown: *m*/*z* 324.16 (pink), *m*/*z* 510.27 (blue) and *m*/*z* 409.22 (green). BPC = base peak chromatogram. (B) Corresponding LC-MS data for *S. albidoflavus* J1074 compared with the *S. albidoflavus* J1074 BGC mutant (Δ*amiD*). (C) Precursor peptide AmiA with leader, core and follower regions highlighted along with the structure of streptamidine. (D) Detailed HMBC and COSY NMR correlation data for streptamidine (associated data shown in Fig. S11–S20 and Table S9†).

To determine the RiPP product of the BGC, an in-frame deletion of the precursor peptide gene, *amiA*, was generated in pCAPSalbC *via* PCR-targeting.^42^ “Wild type” pCAPSalbC and pCAPSalbC *ΔamiA* were introduced into *Streptomyces coelicolor* M1146,^43^*Streptomyces lividans* and *Streptomyces laurentii via* intergeneric conjugation from *Escherichia coli*, and the resulting strains were fermented in multiple media. Untargeted metabolomic analysis of liquid chromatography-mass spectrometry (LC-MS) data revealed three major compounds (*m*/*z* 647.32, *m*/*z* 510.27 and *m*/*z* 409.22) that were produced by *S. coelicolor* M1146 containing the full cluster (*S. coelicolor* M1146-pCAPSalbC) but not the negative control strain that lacked the precursor peptide gene (*ΔamiA*, Fig. 4A and S8†).

Based on similar tandem MS (MS/MS) fragmentation data (Fig. S9†), we proposed that these three compounds were related, and that the smaller masses might represent different intermediates or breakdown products of the final natural product, *m*/*z* 647.32 (also observed as [M + 2H]^2+^, *m*/*z* 324.16). To further confirm that these compounds were produced by the putative BGC, we constructed a mutant disrupted in the YcaO gene (*amiD*) in *S. albidoflavus* J1074 and grew this along with wild type *S. albidoflavus* J1074 under the same conditions as for heterologous expression. LC-MS analysis showed that all identified compounds (*m*/*z* 647.32, *m*/*z* 510.27 and *m*/*z* 409.22) were produced by *S. albidoflavus* J1074 but were not produced by the *ΔamiD* mutant (Fig. 4B). MS/MS fragmentation for *m*/*z* 647.32 was identical in both *S. albidoflavus* and *S. coelicolor* M1146-pCAPSalbC (Fig. S10†). No known natural products with this mass and MS/MS fragmentation could be identified in publicly available databases.^44–46^ These data provided strong support for the hypothesis that the *ami* BGC produces a new type of RiPP.

### Structural elucidation of streptamidine

High-resolution LC-MS/MS data indicated that the compound with *m*/*z* 647.32 corresponds to the proton adduct of molecule with formula C_28_H_42_N_10_O_8_ (calculated [M + H]^+^*m*/*z* 647.3260; observed *m*/*z* 647.3251). An analysis of all possible core peptides from AmiA along with a set of likely modifications indicated that the mass was consistent with a central HLSATH core peptide of AmiA that had undergone dehydration. The formation of an oxazoline would be consistent with ATP-dependent cyclodehydration catalysed by the YcaO-domain protein. Following large-scale fermentation, this compound was purified and the structure was elucidated by NMR (^1^H, ^13^C, COSY, HSQCed, HMBC, TOCSY and HSQC-TOCSY, Table S9, Fig. S11–S20†). This verified that the compound derived from the HLSATH core peptide. However, the chemical shifts for the side-chains of Ser3 (core peptide numbering) and Thr5 were consistent with unmodified amino acids rather than the corresponding heterocycles, whereas the ^13^C shift for the sp^2^ C15 between Leu2 and Ser3 (*δ*_C_ 157.1 ppm) differed from either an unmodified amide carbonyl or an oxazoline ring. Instead, HMBC correlations supported a structure with a 6-membered amidine ring formed between the N-terminal amine of His1 and the carbonyl of Leu2. Correlations are shown in Fig. 4D and include HMBC correlations between C15–H9 (*δ*_H_ 8.05), C15–H7 (*δ*_H_ 3.95–3.91) and C15–H18 (*δ*_H_ 4.30–4.27), which support the presence of an amidine ring. The chemical shift of C15 (*δ*_C_ 157.1 ppm) is similar to that of the corresponding carbons in the amidine rings of bottromycin (*δ*_C_ 157.9 ppm) in CDCl_3_ (ref. 47) and klebsazolicin (*δ*_C_ 156.8 ppm) in DMSO-d_6_.^48^

Marfey's method for amino acid analysis^49^ was used to determine the absolute configuration of this molecule (Fig. S21†). This analysis determined that all amino acids were l-configuration, with the exception of Leu2, which exists as mixture of d- and l-isomers. This may partially account for the multiple peaks observed for *m*/*z* 324.16 by LC-MS (Fig. 4), although multiple protonation states could also contribute. NMR analysis revealed a time-dependent isomerisation that supports a structural change in the amidine ring region of the molecule (Fig. S20 and S22†), which could be associated with spontaneous Leu2 epimerisation.

Due to the widespread presence of this BGC in streptomycetes and the rare amidine ring, this new compound was named streptamidine. The small size of streptamidine and the lack of conventional (ox/thi)azoles prompted further MS analysis using methodology optimised for larger peptides.^50^ In some *S. coelicolor* M1146-pCAPSalbC cultures, a potential pathway-related compound with *m*/*z* 414.69 was observed (Fig. S8†), but this was never observed in *S. albidoflavus* J1074 (Fig. S8†). The production of substantial amounts of streptamidine in *S. albidoflavus* J1074 (Fig. 4B) provided support that streptamidine is the major product of the *ami* BGC. Evidence for streptamidine distribution in nature was assessed by an analysis of mass spectral databases using MASST (Mass Spectrometry Search Tool),^51^ which identified a molecule with identical mass and MS/MS fragmentation to streptamidine in a marine actinomycete MS dataset (Fig. S23,† MassIVE MSV000078679), although the precise identity of this actinobacterium is not known.

High-resolution LC-MS/MS analysis of two other compounds produced by the *ami* BGC (*m*/*z* 510.2668 and *m*/*z* 409.2195), indicated that these have masses that match those calculated for dehydrated HLSAT and HLSA peptides respectively (calculated *m*/*z* 510.2671 and *m*/*z* 409.2196, respectively for [M + H]^+^). These compounds have MS/MS spectra highly similar to streptamidine, including multiple identical fragments that are characteristic of the N-terminal amidine and the presence of histidine and leucine residues (Fig. S9†).

The prevalence of this BGC family across Actinobacteria suggests an important function for streptamidine-like molecules. We hypothesised that this wide distribution could be related to metal import, given the frequent association with *fecBCDE*-like genes. However, metal binding could not be detected with an iron-based CAS assay or with LC-MS-based binding assays with a range of metal ions [iron(ii), cobalt(ii), copper(ii), magnesium(ii), manganese(ii), nickel(ii) and zinc(ii)]. Similarly, the streptamidine-null *S. albidoflavus ΔamiD* mutant was phenotypically identical to wild type *S. albidoflavus* under metal starvation conditions. No antibacterial or antifungal activity could be detected in assays against multiple strains using either purified streptamidine or in co-cultures (Table S10†).

### Identification of key biosynthetic machinery

**Fig. 5:**
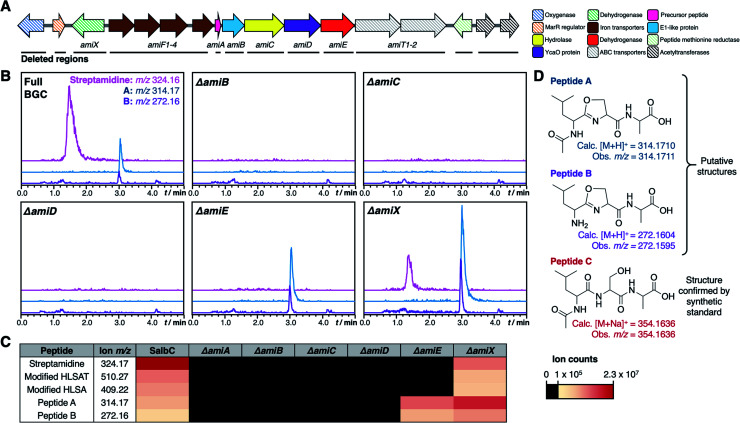
Mutational analysis of *ami* BGC. (A) TAR cloned genetic region from *S. albidoflavus* with minimal biosynthetic gene cluster indicated. Striped arrows represent genes that are not essential for production of streptamidine, filled in arrows represent genes whose deletion abolishes production of streptamidine and are therefore essential for biosynthesis. The black lines beneath the genes indicate the regions that were independently deleted in this study. (B) Metabolomic profiles of expressed gene cluster and key pathway mutants, including extracted ion chromatograms of shunt metabolite masses. (C) Heat map indicating intensity of different metabolites produced by the wild type BGC (SalbC) and pathway mutants. (D) Predicted structures of streptamidine pathway shunt metabolites.

To determine the minimal set of genes required for streptamidine production, we generated a series of in-frame deletion mutants in the pCAPSalbC plasmid (Fig. 5A). Deletion of *amiB* (hypothetical protein), *amiC* (hydrolase), *amiD* (YcaO-like protein), *amiE* (dehydrogenase), and *amiF1–F4* (iron transporters) abolished production of all pathway-associated compounds (Fig. 5B, C and S8†). These data indicated that these genes are essential for biosynthesis and enabled us to determine the minimal *ami* BGC (Fig. 5A). In contrast, deletion of *amiX* (putative oxidoreductase) gene, the MarR gene, the oxygenase gene, the peptide methionine sulfoxide reductase gene and the acetyltransferase genes did not abolish production of the compounds, indicating that these genes are not required for biosynthesis (Fig. S8†). Deletion of *amiT1–T2* (ABC transporters) did not fully abolish production but did substantially decrease streptamidine production (Fig. S8†) and can therefore be considered as important genes for streptamidine biosynthesis.

In addition to abolishing streptamidine production, the dehydrogenase mutant (*ΔamiE*) increased production of additional molecules: *m*/*z* 272.16 and *m*/*z* 314.17. In addition, *m*/*z* 354.16 was seen in some cultures (Fig. S8†), but not consistently. High resolution MS indicated that these molecules could derive from the Leu–Ser–Ala tripeptide within the core peptide (Fig. 5D): dehydrated LSA ([M + H]^+^ calc. *m*/*z* 272.1604, obs. *m*/*z* 272.1595), *N*-acetylated and dehydrated LSA ([M + H]^+^ calc. *m*/*z* 314.1710, obs. *m*/*z* 314.1711) and *N*-acetylated LSA ([M + Na]^+^ calc. *m*/*z* 354.1636, obs. *m*/*z* 354.1636). MS/MS fragmentation for *m*/*z* 272.16 and *m*/*z* 314.17 were consistent with oxazoline-containing molecules (Fig. S24†), while the identity of *N*-acetylated LSA was confirmed by a comparison to a synthetic standard, which has an identical retention time and MS/MS fragmentation (Fig. S25†). The irregular production of this peptide could reflect that it derives from the spontaneous ring-opening of peptide A (*m*/*z* 314.17). Production of these compounds was also increased in the oxidoreductase mutant (*ΔamiX*), although streptamidine was still produced by this strain (Fig. 5B).

### Biosynthesis of the amidine ring

To date, only two RiPPs with amidine rings have been characterised: bottromycin^52^ and klebsazolicin.^48^ In klebsazolicin biosynthesis, the BGC encodes one YcaO-domain protein that installs azole heterocycles and the amidine ring^23^ in cooperation with a partner E1-like protein and a dehydrogenase. The BGC for bottromycin encodes two YcaO proteins, where one is required for macroamidine formation and the other catalyses heterocyclisation of a cysteine residue to a thiazoline; both function without a partner protein.^21,22^ In the case of streptamidine, the gene deletion data are consistent with a role in cyclisation for AmiB and the YcaO protein, AmiD. Conventional sequence analysis did not identify any conserved domains for AmiB, but Phyre2 (ref. 53) analysis predicts that it has a homologous structure to residues 4–315 of the cyanobactin heterocyclase TruD,^20^ encompassing a RiPP recognition element (RRE)^54,55^ and an E1-like domain.^56^ This homology suggests that AmiB and AmiD cooperate to catalyse cyclisation in an analogous way to heterocycle-forming YcaO proteins, although the weak sequence identity with characterised E1-like domains is reflected by the lack of an identifiable RRE in AmiB using RRE-Finder.^55^ AmiD features a proline-rich C-terminus, which is a characteristic feature of azoline-forming YcaO proteins^57^ (Fig. S26†). Deletion of the hydrolase gene *amiC* abolishes streptamidine production, which is consistent with a predicted role of leader peptide removal prior to amidine formation, which requires a free N-terminal amine on His1.

Deletion of genes encoding dehydrogenase AmiE and hypothetical protein AmiX led to the accumulation of molecules with accurate masses consistent with dehydrated LSA derived from the core peptide (Fig. 5). These peptides could result from premature hydrolysis of AmiA during biosynthesis (Fig. S27†), although their formation could partly be a consequence of inefficient processing by the heterologous host. This hints that an oxazoline-containing intermediate could be formed before the final amidine-containing structure is generated, and that the dehydrogenase has a cryptic role in cyclisation. In klebsazolicin biosynthesis, Travin *et al.*^23^ proposed that an intermediate ring structure might form on the Ser3 residue before the amidine is ultimately produced, which could potentially happen in streptamidine biosynthesis (Fig. S27†). In relation to this mechanism, Ser3 of the streptamidine core peptide is conserved across motif A-containing precursor peptides (Fig. S3†), although there is variation elsewhere in this core region. In contrast, there are no heterocycle-forming residues within the equivalent region of motif B peptides (Fig. S4†).

To assess the importance of Ser3 for streptamidine biosynthesis, this residue was mutated to cysteine, as the equivalent mutation in the klebsazolicin pathway previously led to the *in vitro* production of a thiazole instead of an amidine.^21^ A single nucleotide mutation on the core peptide region of *amiA* in pCAPSalbC was made using oligonucleotide-directed mutagenesis^58^ in the *mutS*-deficient strain *E. coli* HME68 (Fig. S28†). However, no pathway-associated metabolites could be detected when pCAPSalbC-S3C was expressed in *S. coelicolor* M1146 (Fig. S28†). Abolition of production suggests that there is a direct role for Ser3 in amidine formation, although this result could instead reflect tight substrate specificity of the pathway.

The production of high levels of streptamidine by both the heterologous host and wild type *S. albidoflavus* J1074 indicates that it is a major product of the pathway, although it is surprising that the dehydrogenase AmiE is essential for streptamidine production given the lack of an oxidation in streptamidine. Possible explanations include: (a) AmiE is fulfilling a key structural role for proper cyclisation activity; (b) AmiE is catalytic but a reductase reverses this activity; (c) the oxidised part of the peptide is hydrolysed from the streptamidine core region. AmiE is highly dissimilar to characterised azole-forming dehydrogenases (∼10% identity to the microcin B17 dehydrogenase McbC) but has a HY motif that structurally aligns with the catalytic KY residues of McbC^59^ (Fig. S29†). Detailed biochemical experiments will be required to determine the precise role of the dehydrogenase in streptamidine biosynthesis.

## Conclusions

This study shows that the application of targeted genome mining tools is a valuable approach to identify uncharacterised novel biosynthetic gene clusters. Using standalone YcaO proteins in Actinobacteria, we identified over 230 novel BGCs that are widespread in well-studied bacteria such as *Streptomyces*, as well as understudied genera such as *Frankia* and *Rhodococcus.* These BGCs all encode a common family of precursor peptides that can be subdivided into two major groups (A and B) based on sequence motifs (Fig. 2A). Guided by genetic and metabolomic analyses, we isolated and characterised streptamidine, a previously overlooked amidine-containing RiPP from *S. albidoflavus* J1074, a model streptomycete.^39^ Streptamidine represents a very rare example of an amidine-containing peptide in nature, yet our analysis indicates that related compounds could be widespread.

The *S. albidoflavus* J1074 precursor peptide sequence contains motif A. The precursor peptides from this group are encoded in BGCs with very conserved genetic architectures, which suggests that a range of close homologues of streptamidine are produced in nature. In contrast, the precursor sequences containing motif B feature very distinct amino acid sequences and are encoded within varied BGC architectures (Fig. S7†). These BGCs might therefore collectively produce a wide range of structurally distinct RiPPs. This highlights that there is still a vast amount of untapped chemical diversity to be discovered from uncharacterised RiPP BGCs, as have other recent studies that have used genomics-led approaches to identify widespread novel RiPP chemistry.^9,60–62^ Along with RiPPER, recent workflows and bioinformatic tools are addressing the challenge of systematically discovering this RiPP novelty.^55,63,64^ An unanswered question about these newly discovered RiPPs is the role of the conserved ‘ALV’ motif present in both motif A and motif B-containing precursor peptides. This motif could represent an important recognition sequence or cleavage site, which would be analogous to cyanobactin precursor peptides. Cyanobactin precursors feature conserved recognition sequences that flank hypervariable core peptides and are important for recognition of modification enzymes.^65,66^

The biological role of streptamidine remains unknown. Interestingly, Metelev *et al.*^48^ observed that the six-membered amidine ring of klebsazolicin is essential for its unique ability to form a compact conformation inside the ribosome exit tunnel and block translation. While streptamidine itself does not possess this activity, the prevalence and widespread distribution of streptamidine-like BGCs in nature indicates that the amidine chemotype is much more prevalent than previously expected and suggests a beneficial role for the producing organism. This is comparable to other widespread natural products whose activities remain a mystery.^67,68^ The resulting molecules may therefore have an important role that could be linked to signalling or development rather than inhibitory activity, which warrants further investigations into this new family of RiPP. Furthermore, the extent of uncharacterised BGCs encoding YcaO proteins alongside diverse precursor peptides (Fig. 2 and S7, ESI datasets 1 and 2†) highlights the wealth of RiPP diversity that remains to be discovered.

## Data availability

The datasets associated with this article are available as part of the ESI.† GenBank files of each BGC region annotated by RiPPER are available online at https://doi.org/10.6084/m9.figshare.14191544. Streptamidine BGC details have been deposited at MIBiG with accession number BGC0002115.

## Author contributions

Alicia H. Russell: investigation, methodology, data curation, visualisation, writing – original draft and review & editing. Natalia M. Vior: investigation, supervision, visualisation, writing – review & editing. Edward S. Hems: investigation, validation, writing – review & editing. Rodney Lacret: investigation, validation. Andrew W. Truman: conceptualisation, project administration, supervision, funding acquisition, methodology, visualisation, writing – original draft and review & editing.

## Conflicts of interest

There are no conflicts to declare.

## Supplementary Material

SC-012-D1SC01456K-s001

SC-012-D1SC01456K-s002

SC-012-D1SC01456K-s003

SC-012-D1SC01456K-s004
