# Supplementary material for: Discovery and characterisation of an amidine-containing ribosomally-synthesised peptide that is widely distributed in nature
Source: Chem Sci. 2021 Aug 2;12(35):11769–78. doi: 10.1039/d1sc01456k (PMC8442711; doi:10.1039/d1sc01456k)
Supplement: SC-012-D1SC01456K-s001 [file SC-012-D1SC01456K-s001.pdf]

Electronic Supplementary Material (ESI) for Chemical Science.
This journal is © The Royal Society of Chemistry 2021

SUPPORTING INFORMATION

Discovery and characterisation of an amidine-containing ribosomally-synthesised peptide that is widely distributed in nature

Alicia H. Russell, Natalia M. Vior, Edward S. Hems, Rodney Lacret and Andrew W. Truman*

Department of Molecular Microbiology, John Innes Centre, Norwich, NR4 7UH, UK

CONTENTS

Materials and Methods ... 3

**Table S1**	Strains used in this study	11
**Table S2**	Plasmids used in this study	11
**Table S3**	Constructs generated in this study	12
**Table S4**	Media used in this study	13
**Table S5**	Solutions used for TAR cloning	13
**Table S6**	Primers used in this study.	14
**Table S7**	Accurate masses of streptamidine and related compounds	16
**Table S8**	Proteins encoded in the streptamidine biosynthetic gene cluster	17
**Figure S1**	Precursor peptide networks associated with actinobacterial YcaO proteins	18
**Figure S2**	YcaO protein phylogenetic tree with branch lengths	19
**Figure S3**	Alignment of precursor peptides containing motif A	20
**Figure S4**	Alignment of precursor peptides containing motif B	22
**Figure S5**	80% identity networking of network 1 precursor peptides	23
**Figure S6**	Phylogenetic tree of YcaO proteins associated with network 1 peptides	24
**Figure S7**	MultiGeneBlast analysis of streptamidine BGC	25
**Figure S8**	Comparative analysis of LC-MS data	27
**Figure S9**	MS/MS fragmentation data for streptamidine and related compounds	28
**Figure S10**	MS/MS comparison between pCAPSalbC and *S. albidoflavus*	29
**Figure S11**	Structure of streptamidine with numbered atoms	29
**Table S9**	NMR chemical shift assignments for streptamidine in DMSO-d_6	30
**Figure S12**	NMR correlation data observed in 2D spectra.	31
**Figure S13**	^{1}H NMR spectrum of streptamidine	32
**Figure S14**	^{13}C NMR spectrum of streptamidine	32
**Figure S15**	2D COSY spectrum of streptamidine	33
**Figure S16**	2D HMBC spectrum of streptamidine	34
**Figure S17**	Selected regions of 2D HMBC spectrum	35
**Figure S18**	2D HSQCed spectrum of streptamidine	36
**Figure S19**	2D TOCSY spectrum of streptamidine	37
**Figure S20**	2D HSQC-TOCSY spectrum of streptamidine	38
**Figure S21**	Marfey's analysis of streptamidine	39
**Figure S22**	NMR analysis of isomerisation	40
**Figure S23**	Match to the MS/MS spectrum of streptamidine identified using MASST	41

Table S10 Indicator strains tested in bioactivity assays 42
Figure S24 LC-MS analysis of Δ*amiE* culture extract and synthetic *N*-acetylated LSA.........43
Figure S25 MS/MS fragmentation data for predicted modified LSA compounds 44
Figure S26 Sequence alignment of YcaO proteins..45
Figure S27 Proposed mechanism of amidine ring formation in streptamidine 46
Figure S28 Mutation of AmiA core peptide 47
Figure S29 Secondary structure alignment of AmiE with McbC. 48
References 49

MATERIALS AND METHODS

Chemicals and media

Unless otherwise stated, all chemicals and media components were purchased from Sigma Aldrich, except for the following: agar (Melford), NaCl, glucose and sorbitol (Fisher Scientific), yeast extract (Merck), soy flour (Holland and Barrett) and peptone (BD Biosciences). Ultrapure water was obtained using a Milli-Q purification system (Merck). All media were autoclaved prior to use and chemical solutions were filter sterilised using a 0.22 µM syringe filter. All primers were ordered from Eurofins Genomics and purified by HPSF. *N*-acetyl-LSA was custom synthesised by Genscript.

Strains and culture conditions

All strains, plasmids, culture media and primers used in this work are described in the Supporting Information (Tables S1-S6). Unless otherwise specified, all *Streptomyces* strains were grown at 28 °C on solid SFM for spore growth, solid SFM supplemented with 10 mM $MgCl_2$ for conjugations, liquid TSB for seed cultures and liquid SM12 media for fermentations. Other media used during screening trials include liquid R5, BPM and SM14. Liquid cultures were grown with shaking at 250 rpm. Spores and mycelium stocks were kept at −20 °C in 20% glycerol. *Saccharomyces cerevisiae* VL6–48N[1] was used for transformation-associated recombination (TAR) cloning and was grown at 30 °C with shaking at 250 rpm in YPD medium. Recombinant yeast selection was performed using selective media SD+CSM-Trp complemented with 5-fluoorotic acid (Fluorochem, 1 mg mL^{-1}). *Escherichia coli* DH5α was used for transformation and propagation of DNA plasmids. For gene deletions, *E. coli* DH5α BT340 was used for Flp-FRT recombination and *E. coli* BW25113/pIJ790 was used for Lambda-Red mediated recombination[2]. pIJ790-carrying strains were grown at 30 °C for plasmid replication, and Flp-FRT recombination was performed at 42 °C. *E. coli* ET12567/pR9604 and *E. coli* ET12567/pUZ8002 were used to transfer DNA to *Streptomyces* by intergeneric conjugation. All *E. coli* strains were grown in LB medium at 37 °C unless otherwise specified. *E. coli* hygromycin selection was performed in solid DNA media. *E. coli* cell stocks were kept at −20 °C in 20% glycerol. Antibiotic selection was carried out using the following final concentrations of antibiotic: kanamycin 50 µg mL^{-1}, apramycin 50 µg mL^{-1}, hygromycin 50 µg mL^{-1}, nalidixic acid 25 µg mL^{-1}, chloramphenicol 25 µg mL^{-1} and carbenicillin 100 µg mL^{-1}.

Analysis of YcaO domain proteins

All actinobacterial standalone YcaO-domain proteins were identified in NCBI Genbank using CDART (Conserved Domain Architecture Retrieval Tool)[3] (2,574 proteins). These were filtered using EFI-EST[4] to 2,338 sequences after excluding proteins smaller than 350 AA, and further filtered to 1,514 proteins using a 95% identity cut-off. Corresponding accession numbers were submitted to Batch Entrez and the resulting sequence files aligned using MUSCLE[5] (version 3.8.31). This alignment was used to construct a maximum likelihood tree using RAxML[6] (version 8.2.12) on the CIPRES

Science Gateway (https://www.phylo.org/) with the following settings: raxmlHPC-HYBRID -T 4 -n result -s infile.txt -p 12345 -m PROTCATDAYHOFF -f a -N 100 -x 12345

The YcaO proteins associated with Network 1 (plus outgroup protein WP_044386624.1) were aligned using MUSCLE. This alignment was used to construct a maximum likelihood tree using RAxML on the CIPRES Science Gateway with the following settings: raxmlHPC-HYBRID-AVX -T 4 -f a -N autoMRE -n result -s infile.txt -p 12345 -m PROTGAMMAWAGF -k -x 12345

The trees were visualised with the interactive Tree Of Life (iTOL)[7].

Retrieval and analysis of precursor peptides and BGCs

The 1,514 YcaO-domain protein accessions were used as the input for RiPPER[8,9] (https://github.com/streptomyces/ripper) with default settings. The resulting GenBank files are available to download online (DOI: 10.6084/m9.figshare.14191544). Some of the precursor peptide sequences obtained from RiPPER analysis were duplicated due to the presence of more than one YcaO protein in some BGCs, so 29 duplicated precursor sequences were manually removed prior to subsequent analysis. Peptide similarity networking of the precursor peptide sequences were created using EGN (Evolutionary Gene and genome Network)[10], visualised with Cytoscape 2.8.3[11] and is provided as Supplementary Dataset 1. Multiple sequence alignments of precursor peptides were performed using ClustalW[12] via MEGA7[13] using default settings, and motifs were searched for using the MEME tool in the MEME suite[14] (http://meme-suite.org/index.html) using classic mode with the site distribution as Any Number of Repetitions and searching for 3 motifs. The captured genomic regions were visualised and analysed in Artemis[15] and Vector NTI[16], and putative BGCs were compared using MultiGeneBlast[17] (http://multigeneblast.sourceforge.net/). Conserved protein domains were analysed using NCBI conserved domain search (https://www.ncbi.nlm.nih.gov/Structure/cdd/wrpsb.cgi) and Phyre2[18]. Network 1 peptides were tested with NeuRiPP using the Parallel CNN network architecture[19].

Non-actinobacterial BGCs were identified by BLAST analysis (Expect threshold = 0.05) of AmiD (WP_008409979.1) with actinobacterial proteins excluded from the results. RiPPER analysis identified related precursor peptides in the phylum Firmicutes. A firmicute YcaO protein (ETB73247.1, *Bacillus* sp. CPSM8) was then used for a further cycle of BLAST/RiPPER to assess for any additional related precursor peptides.

TAR cloning of *Streptomyces albidoflavus* J1074 gene cluster

A vector to capture the gene cluster from *S. albidoflavus* J1074 genomic DNA was constructed by Gibson assembly between a linearised pCAP03 vector[20] and two single-strand oligonucleotides (Salb_TAR_fw and Salb_TAR_rv). The forward and reverse oligonucleotides had 34 and 36 nucleotide homology sequences with pCAP03 respectively. These were designed to generate a vector with 50 and 49 nucleotide homology sequences with upstream and downstream regions of

the gene cluster respectively, either side of an AvrII restriction site. pCAP03 was digested with XhoI and NdeI, and 100 ng linearised plasmid and 10 pmol of each oligonucleotide were incubated with 5 μL ligase-free Gibson assembly reaction (100 mM Tris-HCl pH 7.5, 10 mM $MgCl_2$, 0.2 mM each dNTPs, 10 mM DTT, 1 mM NAD, 5% PEG-8000, 0.1125 units T5 exonuclease, 0.375 units Phusion polymerase, 10 μL total reaction volume) and incubated at 50 °C for 2 hours in a BioRad T100 ThermoCycler. 10 μL assembly reaction was then transformed into *E. coli* DH5α by chemical transformation selected with kanamycin. Colonies containing the correct capture vector were identified by PCR using primers pCAP_sp and pCAP_asp, and the plasmid was isolated using a Promega Wizard Plus SV Minipreps DNA Purification System.

Genomic DNA from *S. albidoflavus* J1074 was digested with NsiI and SmlI, and the capture vector was linearised between the capture arms with AvrII. Digested material was transformed into *S. cerevisiae* VL6–48N by spheroplast polyethylene glycol 8000 transformation. Successful gene cluster capture by pCAP03 was confirmed by colony PCR. For yeast-colony PCR, each colony was resuspended in 50 μL 1 M sorbitol (Fisher) and 2 μL of zymolyase (5 U μL^{-1}) was added to each cell suspension and incubated at 30 °C for 1 hour. Cell suspensions were then boiled for 10 min, centrifuged (15 s, 1,000 × *g*) and 1 μL of the supernatant was analysed by PCR using primers Salb_TARscr_Fw and Salb_TARscr_Rv. The plasmids from four positive clones were recovered and transformed into electrocompetent *E. coli* DH5α for further analytical digest of the purified construct with HindIII-HF and SrfI to confirm the identity of pCAPSalbC. The streptamidine BGC is shown in Table S8 and has been deposited at MIBiG[21] with accession number BGC0002115.

Conjugation of pCAPSalbC into *Streptomyces coelicolor* M1146

E. coli ET12567/pR9604 was transformed with pCAPSalbC by electroporation, and transformants were then used to transfer pCAPSalbC into *S. coelicolor* M1146 by intergeneric conjugation. Nalidixic acid and kanamycin-resistant exconjugants containing integrated pCAPSalbC (*S. coelicolor* M1146-pCAPSalbC) were verified by PCR using Promega GoTaq polymerase with primers Salb_TARscr_Fw and Salb_TARscr_Rv.

Construction of pathway mutant in *S. albidoflavus* J1074

A fragment of DNA corresponding to the translationally coupled YcaO and hydrolase genes was PCR cloned with restriction sites for EcoRI and HindIII. The DNA fragment was digested with EcoRI and HindIII and ligated into the pKC1132 plasmid digested with EcoRI and HindIII. The resulting DNA construct was isolated and transferred into *S. albidoflavus* J1074 via intergenic conjugation with *E. coli* ET12567/pUZ8002 selected with apramycin, chloramphenicol and kanamycin. Exconjugants resistant to apramycin were validated by PCR to confirm that the YcaO and hydrolase genes had been disrupted.

Deletion of genes in pCAPSalbC

Mutations of the *S. albidoflavus* J1074 BGC were carried out using an *E. coli*-based Lambda-Red-mediated PCR-targeting strategy[2], which allowed substitution of genes in pCAPSalbC by a PCR-generated cassette containing the apramycin resistance gene *aac(3)-IV*. Resistance cassettes were amplified by PCR using a pIJ773-derived cassette lacking OriT as a template, which allowed the elimination of the apramycin resistance cassette after Flp-FRT recombination in *E. coli* DH5α BT340. This created mutants with an in-frame 81 bp scar in the place of the original gene sequence. The PCR-targeting mutant versions of pCAPSalbC were introduced into *S. coelicolor* M1146 by *E. coli* ET12567/pR9604-mediated intergeneric conjugation and selected by resistance to nalidixic acid and kanamycin.

Complementation of deleted genes in *S. coelicolor* M1146-pCAPSalbC

Constructs for the complementation of mutants were obtained by high-fidelity PCR amplification (Q5 polymerase) of each of these genes, digestion of the PCR product with NdeI and HindIII and cloning by ligation (T4 DNA ligase, Invitrogen) into pIJ10257 digested with NdeI and HindIII. Ligation mixtures were transformed into chemically competent *E. coli* DH5α and the plasmids were recovered by miniprep and then sequenced. The constructs were introduced into the corresponding *S. coelicolor* M1146-pCAPSalbC mutants by *E. coli* ET12567/pR9604-mediated intergeneric conjugation. Exconjugants were selected by resistance to nalidixic acid, kanamycin and hygromycin.

Mutation of *amiA* in pCAPSalbC

Amino acids within the streptamidine core peptide were mutated using a Lambda-Red-mediated recombination strategy in *E. coli* cells deficient in mismatch repair[22]. *E. coli* HME68 cells were grown in LB (10 mL) with chloramphenicol and grown overnight at 30 °C with shaking at 250 rpm. 300 µL of this culture was used to inoculate LB (15 mL) with chloramphenicol, which was grown for four hours at 30 °C with shaking at 250 rpm. Recombineering activity was induced by incubating the cells for 20 minutes at 42 °C with shaking. Cells were then made electrocompetent and transformed with the pCAPSalbC construct. A 63 bp oligonucleotide (SalbPP_Ser3CysRV) was designed containing the desired amino acid mutation flanked by 30 bp regions of homology on either side. *E. coli* HME68-pCAPSalbC cells were then co-transformed with 1 µL (4 pmol) of the mutant oligonucleotide and 1 µL (4 pmol) of oligo100, which produces Gal^+ recombinants of HME68 cells.

Recombinant cells were grown for 2 days at 30 °C on MacConkey agar containing 1% galactose, and selected for using red/white screening. Red colonies were re-streaked as patches on MacConkey agar containing 1% galactose and grown for a further 2 days at 30 °C. Mutations were then screened for by PCR using screening primers (SalbPPmut_FWScr and SalbPPmut_RVScr) binding ~300 bp each side of the desired mutation. Mixed populations of wild type and mutant genes were identified from sequencing data by identifying overlapping peaks for both the wild type and

mutant codons in the sequence chromatogram. Plasmid DNA from corresponding cells was isolated and transformed into *E. coli* DH5α cells. Individual clones were screened for as before for pure mutants. Mutant plasmids were transformed into *S. coelicolor* M1146 by *E. coli* ET12567/pR9604-mediated intergeneric conjugation.

Fermentation screening for streptamidine production

Seed cultures of *S. coelicolor* M1146-pCAPSalbC were prepared by fermentation in a 50 mL flask containing 5 mL of TSB with kanamycin selection for 48 h. 500 µL seed culture was used to inoculate 10 mL SM12, SM14, BPM and R5 in 50 mL Falcon tubes with caps replaced by foam bungs. Control strains carrying the TAR clone with a precursor peptide gene deletion were cultured in the same way for comparison. Seed cultures of *S. albidoflavus* J1074 were grown in the same way with no antibiotic selection, and the *S. albidoflavus ΔamiD* mutant seed cultures were grown with apramycin selection. All fermentations were conducted in triplicate and incubated at 28 °C with shaking at 230 rpm. 1 mL culture samples were taken at day 4, mixed with one volume of methanol and agitated for 30 min at room temperature. These mixtures were then centrifuged (15,871 × *g*, 5.5 min) and 800 µL of the resulting supernatant was transferred to glass vials for liquid chromatography–mass spectrometry (LC–MS) analysis.

LC-MS analysis

LC-MS samples were analysed on a Shimadzu Nexera X2 UHPLC coupled to a Shimadzu IT-TOF mass spectrometer. 5 µL samples were injected onto a Phenomenex Luna Omega 1.6-µm Polar C18 column (50 mm x 2.1 mm, 100 Å) set at a temperature of 40 °C and eluting with a linear gradient of 0–60% methanol in water + 0.1% formic acid over 6 minutes with a flow-rate of 0.6 mL min^{-1}. Positive mode mass spectrometry data was collected between *m/z* 200 and 2,000. Tandem MS (MS/MS) data were collected between *m/z* 50 and 2000 in a data-dependent manner for parent ions between *m/z* 200 and 2000, using collision-induced dissociation energy of 50% and a precursor ion width of 3 Da. The instrument was calibrated using sodium trifluoroacetate cluster ions prior to every run. Untargeted comparative metabolomics was carried out on data from triplicate samples using Profiling Solution 1.1 (Shimadzu) with an ion *m/z* tolerance of 100 mDa, a retention time tolerance of 0.1 min, an ion intensity threshold of 100,000 units, LabSolutions compatible ion *m/z* tolerances = ON, De-Isotope matrix = ON.

For accurate mass analysis, mass spectra were acquired by LC–MS on a Synapt G2-Si mass spectrometer equipped with an Acquity UPLC (Waters). Samples were injected onto an Acquity UPLC BEH C18 column, 1.7 µm, 1 × 100 mm (Waters) and eluted with a gradient of (B) acetonitrile/0.1% formic acid in (A) water/0.1% formic acid with a flow rate of 0.08 mL min^{-1} at 45 °C. The concentration of B was kept at 1% for 1 min followed by a gradient up to 60% B over 10 min,

and then up to 99% over 2 min. MS data were collected with the following parameters: resolution mode, positive ion mode, scan time 0.5 s, mass range *m/z* 50–1200 (calibrated with sodium formate), capillary voltage = 3.0 kV; cone voltage = 40 V; source temperature = 110 °C; desolvation temperature = 250 °C. Leu-enkephalin was used to generate a lock-mass calibration with *m/z* = 556.2766 measured every 30 s during the run. Comparative metabolomic analyses were carried out on the Synapt G2-Si using the same settings as above but with a mass range of *m/z* 100–3000.

Additional MS/MS data were acquired on a Hybrid Quadrupole-Orbitrap Q-Exactive mass spectrometer coupled to a Vanquish UHPLC system (Thermo Scientific). 5 µL samples were injected onto a Phenomenex Luna Omega 1.6-µm Polar C18 column (50 mm x 2.1 mm, 100 Å) applying the same chromatography parameters used in the Shimadzu LC-MS analysis described above. Mass spectra were obtained in positive mode using full MS/dd-MS^2 acquisition settings with the following specific parameters: chromatography peak width = 7s; Full MS settings: resolution = 70,000, AGC target = $3x10^6$, maximum IT = 100 ms, scan range 150 to 2000 *m/z*; dd-MS^2 settings: resolution 17,500, AGC target = $1x10^5$, maximum IT = 50 ms, loop count = 5, isolation window 1.5 *m/z*, (N)CE/ stepped nce 20, 40, 60; dd settings: minimum AGC target = $8x10^3$, exclude isotopes ON, dynamic exclusion = 1s.

Streptamidine-like molecules were searched for using a MASST search[23] at Global Natural Products Social Molecular Networking (https://gnps.ucsd.edu/ProteoSAFe/static/gnps-splash.jsp) in January 2021. A single spectrum search (version release 27) was carried out using the following settings: parent mass tolerance = 2 Da; min matched peaks = 5; ion tolerance = 0.5 Da; score threshold = 0.7; library = speclibs.

Purification of streptamidine

Four 2-litre flasks containing 0.5 L of SM12 were each inoculated with 25 mL of *S. coelicolor* M1146-pCAPSalbC TSB seed culture grown for 48 hours at 28 °C. After four days fermentation at 28 °C with shaking at 250 rpm, the cultures were centrifuged to remove debris, combined and filtered to yield approximately 1.5 L of crude extract. The crude extract was extracted with ethyl acetate (3 × 1.5 litres). The aqueous layer was further extracted with 1-butanol (3 × 1.0 litres). The resulting aqueous extract was concentrated to 50 mL using a Buchi rotary evaporator and subjected to solid-phase chromatography (SPE) on a HP20 cartridge using a gradient of H_2O-MeOH (100:0 to 0:100). Methanol and water were removed using a Buchi rotary evaporator until the samples were concentrated to approximately 10 mL. Samples were then subject to semi-preparative HPLC using a Phenomenex Luna PFP(2) column (5 µm, 250 x 10 mm) with a gradient of aqueous 0.1% formic acid-MeOH (98:2 to 90:10) over 35 minutes with a flow rate of 2 mL min^{-1}. The compound was monitored at a UV wavelength of 210 nm and fractions were assessed by LC-MS. Fractions containing streptamidine were combined and freeze dried. A final purification step was then carried

out using a semi-preparative Luna Omega Polar C18 column (5 µm, 250 x 10 mm), with an isocratic gradient of aqueous 0.1% formic acid-MeOH (90:10) for 16 minutes followed by a wash gradient from 90:10 to 5:95 over 5 minutes with a flow rate of 2.8 mL min^{-1}. The compound was monitored at a UV wavelength of 210 nm and fractions assessed by LC-MS, yielding 1.4 mg pure streptamidine.

Structural elucidation of streptamidine

Streptamidine (1.4 mg) was dissolved in 600 µL DMSO-d_6 from an individual vial and subjected to a series of 1D and 2D nuclear magnetic resonance (NMR) experiments on a Bruker Ascend 600 MHz instrument at 298 K. The NMR experiments carried out were Proton (64 scans), Carbon (25,000 scans), HSQCed (100 scans), HMBC (64 scans), COSY (16 scans), TOCSY (32 scans) and HSQC-TOCSY (64 scans). Spectra were analysed using Bruker TopSpin 3.5 and Mestrelab Research Mnova 14.0 software. NMR data are reported in Figures S11-S20 and Table S9.

Marfey's analysis

500 µg of pure streptamidine was hydrolysed for 16 hours with 100 µL 6 M HCl at 100 °C in a sealed glass vial contained within a heated sand block. The HCl was dried off under a stream of N_2 for 1 hour at room temperature. The hydrolysed sample was mixed with 20 µL 1 M $NaHCO_3$ and 40 µL Marfey's reagent (L-FDAA [Nα-(2,4-dinitro-5-fluorophenyl)-L-alaninamide] 1% solution in acetone) and incubated at 40 °C for 1 hour. The reaction was then neutralised with 20 µL 1 M HCl. The samples were diluted with 500 µL 50% acetonitrile and centrifuged for 1 minute at 15,871 × *g* to remove debris. For derivatisation of amino acid standards, 50 µL of each L- and D- amino acid (histidine, leucine, serine, alanine, threonine, 2 mg mL^{-1} in H_2O) was mixed with 20 µL 1 M $NaHCO_3$ and 40 µL L-FDAA in a 1.5 mL Eppendorf tube and incubated at 40 °C for 1 hour. The reaction was then neutralised with 20 µL 1 M HCl. The samples were diluted with 1 mL 50% acetonitrile and centrifuged for 1 minute at 15,871 × *g* to remove debris. 1 µL each sample were injected onto a Phenomenex Kinetex 1.6-µm C18 column (50 mm x 2.1 mm, 100 Å) set at a temperature of 40 °C and eluting with a linear gradient of (B) 50% acetonitrile from 5-50% in (A) H_2O + 0.1% formic acid over 12 minutes with a flow rate of 0.6 mL min^{-1}. Samples were analysed on a Shimadzu Nexera X2 UHPLC coupled to a Shimadzu IT-TOF mass spectrometer.

Antimicrobial assays

10 mL cultures of each indicator strain (Table S14) were grown in LB (YPD for *Candida utilis*) overnight at 37 °C (30 °C for *C. utilis*). 100 µL of each culture was then used to inoculate a 10 mL subculture of each strain in the same medium, which were grown for 5 hours at 37 °C (30 °C for *C. utilis*). 1 mL of each culture was then mixed with 14 mL molten LB agar, which was poured into plates. Once solidified, three 1 cm diameter plugs were taken from each agar plate, which were then separately loaded with 50 µL streptamidine (1 mg mL^{-1}), 50 µL kanamycin, apramycin or nalidixic

acid (1 mg mL^{-1}) as a positive control and 50 µL water as a solvent control. Plates were incubated overnight at 37 °C (30 °C for *C. utilis*).

Metal binding assays

For the CAS assay, 500 µL of CAS assay solution (prepared as described by Alexander and Zuberer[24]) was mixed with 10 µL increasing concentrations of streptamidine from 1.5 µM to 25 µM. For LC-MS binding assays, solutions of 10 mM metal salts were prepared ($FeCl_3$, $CoCl_2$, $CuCl_2$, $MgCl_2$, $MnSO_4$, $NiSO_4$, $ZnCl_2$, dissolved in 10 mM HCl) and 500 µL of each were mixed with 20 µL streptamidine (15 µM). 5 µL samples were analysed on a Shimadzu Nexera X2 UHPLC coupled to a Shimadzu IT-TOF mass spectrometer with a Phenomenex Luna Omega 1.6-µm Polar C18 column (50 mm x 2.1 mm, 100 Å) set at a temperature of 40 °C and eluting with a linear gradient of 0–60% methanol in water + 0.1% formic acid over 6 minutes with a flow-rate of 0.6 mL min^{-1}. Positive mode mass spectrometry data was collected between *m/z* 200 and 2,000.

For metal starvation experiments, a minimal medium was prepared (2 g K_2SO_4, 3 g K_2HPO_4, 1 g NaCl, 5 g NH_4Cl, 0.005 mg $CuSO_4$, 0.035 mg $MnSO_4.H_2O$, 2 mg $ZnSO_4.7H_2O$, 80 mg $MgSO_4.7H_2O$, 100 mg $CaCl_2.2H_2O$, 2.5% glycerol) as described by Müller and Raymond[25] in glassware washed with EDTA. A series of the same minimal media omitting either copper, zinc or iron were also prepared. Seed cultures of *S. albidoflavus* J1074 and the *S. albidoflavus ΔamiD* mutant were prepared by fermentation in a 50 mL flask containing 5 mL of TSB (with apramycin selection for the *ΔamiD* mutant) for 48 h. 500 µL each seed culture was used to inoculate 10 mL minimal media and each metal dropout media in 50 mL Falcon tubes with caps replaced by foam bungs. Cultures were grown for 4 days and 1 mL samples were taken for LC-MS analysis as described above.

Table S1 Strains used in this study.

Strain	Genotype/description	Application
Saccharomyces cerevisiae VL6-48N[1]	*MATα*, *his3-Δ1, trp1-Δ1, ura3-Δ1, lys2, ade2-101, met14 cir°*	TAR cloning
Escherichia coli DH5α	F^- ϕ80 *lacZ*ΔM15 Δ(*lacZYA-argF*)U169 *recA*1 *endA*1 *hsdR*17 (r_k^-, m_k^+) *phoAsupE*44 *thi*-1 *gyrA*96 *relA*1 λ^-	Transformation and maintenance of plasmids and constructs
E. coli ET12567/pUZ8002	*dam-13::Tn9 dcm-6 hsdM* Cml^R, carrying helper plasmid pUZ8002	Conjugations with pKC1132 disruption construct
E. coli ET12567/ pR9604	*dam-13::Tn9 dcm-6 hsdM* Cml^R, carrying helper plasmid pR9604	Conjugations with TAR cloned BGC, mutated BGC and complementation constructs
E. coli DH5α/BT340	*E. coli* DH5α carrying BT340 plasmid.	Gene deletions
E. coli BW25113/pIJ790	*(Δ(araD-araB)567, ΔlacZ4787(::rrnB-4), lacIp-4000(lacIQ), λ-,rpoS369(Am), rph-1, Δ(rhaD-rhaB)568, hsdR514. Plasmid: pIJ790 [oriR101], [repA101(ts)], araBp-gam-be-exo*	Gene deletions
E. coli HME68	W3110 *galKtyr145UAG ΔlacU169 [λ cI857 Δ(cro-bioA)] ΔmutS*	Mutagenesis of core peptide amino acids
Streptomyces albidoflavus J1074[26]	Restriction-defective derivative (R^-M^-) of *S. albidoflavus* G	Genetic source of BGC
Streptomyces coelicolor M1146[27]	Δ*act* Δ*red* Δ*cpk* Δ*cda*	Heterologous expression of gene cluster

Table S2 Plasmids used in this study.

Plasmid	Features	Resistance marker	Application
pCAP03[20]	ARSH4/CEN6-Trp1, pUC ori, C31 int-attP-oriT-aph, URA3, ADH1	Kanamycin	TAR cloning
pKC1132[28]	Conjugative vector, non-integrative, lacZa	Apramycin	*S. albidoflavus* pathway disruption
pIJ773[2]	oriT, non-conjugative, flippase recognition target (FRT) sites	Apramycin	Gene deletions via PCR targeting
pIJ10257[29]	ΦBT1, p*erm*E*	Hygromycin	Genetic complementation of mutants

Table S3 Constructs generated in this study.

Construct	Resistance marker	Application
pCAPSalbC	Kanamycin	pCAP03-based plasmid containing the TAR cloned *S. albidoflavus* BGC
pCAPSalbC_ΔPP	Kanamycin	pCAPSalbC with deletion of precursor peptide gene
pCAPSalbC_ΔYcaO	Kanamycin	pCAPSalbC with deletion of YcaO gene
pCAPSalbC_ΔE1	Kanamycin	pCAPSalbC with deletion of E1-like gene
pCAPSalbC_ΔDehy	Kanamycin	pCAPSalbC with deletion of dehydrogenase gene
pCAPSalbC_ΔHydro	Kanamycin	pCAPSalbC with deletion of hydrolase gene
pCAPSalbC_ΔOxido	Kanamycin	pCAPSalbC with deletion of oxidoreductase gene
pCAPSalbC_ΔABC	Kanamycin	pCAPSalbC with deletion of set of ABC transporter genes
pCAPSalbC_ΔIrTr	Kanamycin	pCAPSalbC with deletion of set of iron transporter genes
pCAPSalbC_ΔPepMet	Kanamycin	pCAPSalbC with deletion of peptidyl methionine gene
pCAPSalbC_ΔOxyg	Kanamycin	pCAPSalbC with deletion of oxygenase gene
pCAPSalbC_ΔAcet	Kanamycin	pCAPSalbC with deletion of both acetyltransferase genes
pCAPSalbCSer3Cys	Kanamycin	pCAPSalbC with core peptide Ser3 to Cys3 mutation
pKC1132_SalbYH	Apramycin	Construct for disruption of *S. albidoflavus* BGC in native host
pIJSalb_YcaO	Hygromycin	Construct for complementation of YcaO deletion
pIJSalb_E1	Hygromycin	Construct for complementation of E1 deletion
pIJSalb_Dehy	Hygromycin	Construct for complementation of dehydrogenase deletion

Table S4 Media used in this study.

Medium	Application	Ingredients per 1 L, made up with milliQ water
LB	*E. coli*	10 g Bacto-tryptone, 5 g yeast extract, 10 g NaCl, adjust to pH 7 with NaOH
DNA	*E. coli*	4 g Difco Nutrient Broth powder, 10 g agar
MacConkey agar	*E. coli*	17 g peptone, 3 g protease peptone, 10 g lactose monohydrate, 1.5 g bile salts, 5 g NaCl, 0.03 g neutral red, 0.001 g crystal violet, 13.5 g agar
TSB	*Streptomyces*	17 g tryptone, 3 g phytone, 5 g NaCl, 2.5 g K_2HPO_4, 2.5 g glucose
SFM	*Streptomyces*	20 g mannitol, 20 g soya flour, 100 mM $CaCl_2$, 20 g agar
YPD	*Saccharomyces*	10 g yeast extract, 20 g peptone, 20 g glucose (15 g agar), 0.004% adenine
SD-Trp	*Saccharomyces*	5 g $(NH_4)_2SO_4$, 1.7 g YNB-AA, 20 g glucose, 0.74 g CSM-Trp (20 g agar), 0.004% adenine
Top selective agar	*Saccharomyces*	182 g sorbitol, 22 g dextrose, 30 g agar, 0.0002% 5-FOA, 0.004% adenine
Bottom selective agar	*Saccharomyces*	182 g sorbitol, 22 g dextrose, 20 g agar, 0.0002% 5-FOA, 0.004% adenine
R5	*Streptomyces*	103 g sucrose, 0.25 g K_2SO_4, 10.12 g $MgCl_2.6H_2O$, 10 g glucose, 0.1 g casamino acids, 2 mL trace element solution, 5 g yeast extract, 5.73 g TES buffer
SM12	*Streptomyces*	10 g soy flour, 50 g glucose, 4 g peptone, 4 g beef extract, 1 g yeast extract, 2.5 g NaCl, 5 g $CaCo_3$, adjust to pH 7.6 with KOH
SM14	*Streptomyces*	10 g glucose, 20 g soy peptone, 5 g meat extract, 5 g NaCl, 0.01 g $ZnSO_4.7H_2O$, adjust to pH 7.0 with KOH
BPM	*Streptomyces*	15 g starch, 5 g yeast extract, 10 g soy flour, 5 g NaCl, 3 g $CaCO_3$, 25 µg/mL $CoCl_2$

Table S5 Solutions used for TAR cloning.

Solution	Ingredients per 100 mL, made up with milliQ water
10x nitrogen bases	1.9 g YNB-AA, 1.9 g CSM-Trp, 5 g NH_4SO_4
100x adenine	1 g adenine, 74 mM HCl
SPE	10 mM HEPES buffer pH 7.5, 100 mM EDTA pH 8, 18.2 g sorbitol
SOS	15 mM $CaCl_2$, 0.25 g yeast extract, 18.2 g sorbitol, 1 g peptone
STC	10 mM Tris.HCl pH 7.5, 10 mM $CaCl_2$, 18.2 g sorbitol
PEG	10 mM Tris.HCl pH 7.5, 10 mM $CaCl_2$, 20 g PEG8000

Table S6 Primers used in this study.

Primer name	Sequence (5'-3')	Use	Restriction site
SalbCap_Fw	GCTGCCGGGCCGGCTCCTAGGTCTACATCGGGGACATCAGCGACGCCCGTCCCGCGAGTCTTCCGATGCCGTTAATTAAGCCACTATTTATACCATGGGAGGCGTCAAAC	Construction of pCAP03-derived capture vector for TAR cloning	-
SalbCap_Rv	TGTCCCCGATGTAGACCTAGGAGCCGGCCCGGCAGCTGACGGGTCAGCCACGGCAGGAACCGCGGGCCGTCATATGTCGAAAGCTACATATAAGGAACGTGCTG		-
Salb_ClusScr_Fw	GCAGGACGGAACCGAGGGATG	Screening for cluster capture	-
Salb_ClusScr_Rv	TGGGAGAGGATCGCCTCGGC		-
Salb_YHMut_Fw	GATACAAAGCTTGACTGGATACGCGCCCAGC	Amplification of DNA fragment for pathway disruption in *S. albidoflavus*	HindIII
Salb_YHMut_Rv	GATACAGAATTCGCTCACCTCCAGGCCGGACC		EcoRI
Salb_TAR_PPDel_Fw	CGTCCACCACGCATCGAACTGAATGGAGCTCAACT CATGATTCCGGGGATCCGTCGACC	Precursor peptide gene deletion	-
Salb_TAR_PPDel_Rv	TGTCAGCCGGCCGCCGTCACCGGCGGCCTGGGCTGACTATGTAGGCTGGAGCTGCTTC		-
Salb_TAR_E1Del_Fw	GCCCATCCCCTCGTACTCCATCCGACGGAGGTTTCCGTGATTCCGGGGATCC	E1-like gene deletion	-
Salb_TAR_E1Del_Rv	CGGGCCGCTCCCCCTTGCGGGCAGCGGGGCCACCGGCGGTGTAGGCTGGAGCTGCTTC		-
Salb_TAR_YcaODel_Fw	GACCTGCCGATGACCGCCGCCCTGCCCCTCGACGCCCTCATTCCGGGGATCCGTCGACC	YcaO gene deletion	-
Salb__TAR_YcaODel_Rv	GGGAGCCGGGGGTCACATGTGCGGGGCGGGGGCGAGGGTTGTAGGCTGGAGCTGCTTC		-
Salb_TAR_HydrDel_Fw	GCTGCCCGCAAGGGGGAGCGGCCCGGCATGACGCCCGCGATTCCGGGGATCCGTCGACC	Hydrolase gene deletion	-
Salb_TAR_HydrDel_Rv	GGGCGTCGAGGGGCAGGGCGGCGGTCATCGGCAGGTCTCTGTAGGCTGGAGCTGCTTC		-
Salb_TAR_DehyDel_Fw	CGCCCCCGCCCCGCACATGTGACCCCCGGCTCCCCCATGATTCCGGGGATC	Dehydrogenase gene deletion	-
Salb_TAR_DehyDel_Rv	GGGTGAGGTGGTCGGGGGCGGGCCGTGCGCGGCGGCTCATGTAGGCTGGA		-
Salb_TAR_OxidoDel_Fw	CGCCCGGCCCCCGCACCCCCTACCGAGGAGTTCCCCGTGATTCCGGGGATCCGTCGACC	Oxidoreductase gene deletion	-
Salb_TAR_OxidoDel_Rv	GCGACACCCTGGCCCGCGCCTGGCCGAGCTGAGGAGTCATGTAGGCTGGAGCTGCTTC		-
Salb_TAR_MarRDel_Fw	AGGGACGCTACACGACGAGCGAGGAGACCCGCGACCATGATTCCGGGGATCCGTCGACC	MarR regulator gene deletion	-
Salb_TAR_MarRDel_Rv	ACGGGGCGGAGGCGGACCCGGTGGGGCGGTGACTCCTCATGTAGGCTGGAGCTGCTTC		-

Primer name	Sequence (5'-3')	Use	Restriction site
Salb_TAR_Oxyg_Fw	TCGAAGTTCACCATCCAGCAGCGCGCGGTTCCC GCGATGATTCCGGGGATCCGTCGACC	Oxygenase gene deletion	-
Salb_TAR_Oxyg_Rv	CGTTCTCGCTCATGCGCGTCTCCTTCCTCGGCTC GTTCATGTAGGCTGGAGCTGCTTC		-
Salb_TAR_IrTr_Fw	ACGCGTCCCCGCAGGACGGAACCGAGGGATGAA GCCATGATTCCGGGGATCCGTCGACC	Iron transporters gene deletions	-
Salb_TAR_IrTr_Rv	GGTCGGTTCGTCGAGGAGGAGGGTCCGGGTGTC CTGGGCTGTAGGCTGGAGCTGCTTC		-
Salb_TAR_ABC_Fw	CGACCACCTCACCCCGCCCGGAGAGACGTACCC GCGATGATTCCGGGGATCCGTCGACC	ABC transporters gene deletions	-
Salb_TAR_ABC_Rv	CGTGTGCGTGCGCGTGTACGCACGCTTCGGTGC GGGTCATGTAGGCTGGAGCTGCTTC		-
Salb_TAR_PepMet_Fw	CCGGGCGCATGTCGATGCCAGTCGGGAGCACAG CGTATGATTCCGGGGATCCGTCGACC	Peptide methionine sulfoxide reductase MsrA gene deletion	-
Salb_TAR_PepMet_Rv	CCCCGGCTCCTCGGTCCGGTGAAGGAGTGCTGT GGCTCATGTAGGCTGGAGCTGCTTC		-
Salb_TAR_Acet_Fw	CCGCCGGTCCGCACGCCACCGGGAGGGGCCCA CCGCATGATTCCGGGGATCCGTCGACC	Acetyl transferase and maltose-O-acetyltransferase gene deletions	-
Salb_TAR_Acet_Rv	GGGCCCGGCCCCCTTCGCGTGTACGTACGGGCC CCGTCATGTAGGCTGGAGCTGCTTC		-
SalbCycl_PE_ Fw	GATACACATATGACCAGCAGCCGACTCGCC	Complementation of E1-like protein	NdeI
SalbCycl_PE_ Rv	GATACAAAGCTTGTGGTCGCGGGCGTCATGC		HindIII
SalbYcaO_PE_Fw	GATACACATATGACCGCCGCCCTGCCC	Complementation of YcaO	NdeI
SalbYcaO_PE _Rv	GATACAAAGCTTGGGGAGCCGGGGGTCACATG		HindIII
SalbDehy_PE _Fw	GATACACATATGACCCCTGACGCCACCCTCG	Complementation of dehydrogenase	NdeI
SalbDehy_PE _Rv	GATACAAAGCTTGCTCATCGGGCGGCTCCCAG		HindIII
PPDel_screen_Fw	GCGGCTGGCCGGTCTGTTAC	Screening precursor peptide gene deletion	-
PPDel_screen_Rv	CGGCTGCTGGTCACGGAAACC		-
CyclDel_screen_Fw	GGTGCCGCGGACGACAAGTAG	Screening E1-like gene deletion	-
CyclDel_screen_Rv	GTCGTACGGGGTGCGGATCAG		-
YcaODel_screen_Fw	ACCAGGCTGCGCGTCGAGA	Screening YcaO gene deletion	-
YcaODel_screen_Rv	AGGTGGTCGAGGTCGACGGG		-
HydrDel_screen_Fw	CTCCTCACCGCCGACCTCCTC	Screening hydrolase gene deletion	-
HydrDel_screen_Rv	GTGCCGAGCGAGACCCGGT		-
DehyDel_screen_Fw	TCGACCTGACCACCGAGGACG	Screening dehydrogenase gene deletion	-
DehyDel_screen_Rv	ACCAGGGCGAGCAGGGCG		-
OxidoDel_Screen_Fw	CATCCCTCGGTTCCGTCCTG	Screening oxidoreductase deletion	-
OxidoDel_Screen_Rv	AGCACCCTGATCCGGCTGAC		-
Salb_oxyg_scr_Fw	CAGTTGAGGGGCGGATCGTTC	Screening oxygenase deletion	-
Salb_oxyg_scr_Rv	GAAAGGCCCAGCTGGGCGTC		-
Salb_IronTr_scr_Fw	CAGAGGCGTCCCACGCGTC		-

Primer name	Sequence (5'-3')	Use	Restriction site
Salb_IronTr_scr_Rv	GAACGGCGTGGCGACTGCC	Screening iron transporters deletion	-
Salb_ABCTr_scr_Fw	CTCACCCCGCCCGGAGAGAC	Screening ABC transporters deletion	-
Salb_ABCTr_scr_Rv	GTGCGTGCGCGTGTACGCAC		-
Salb_PepMet_scr_Fw	GACCCCGGCTCCTCGGTCC	Screening peptide methionine sulfoxide reductase MsrA deletion	-
Salb_PepMet_scr_Rv	CGCATGTCGATGCCAGTCGG		-
Salb_acet_scr_Fw	GTGACACCAAGGTGCCGCGAAC	Screening acetyl transferase and maltose-O-acetyltransferase deletions	-
Salb_acet_scr_Rv	GGCCCCCTTCGCGTGTACG		-
MarR_screen_Fw	TCAGCCCGACCGGTCCTG	Screening MarR deletion	-
MarR_screen_Rv	CGACCACGCCGAGGAGGTC		-
pIJ10257_Fw	TTCGAGTGGCGGCTTGCG	Screening for pIJ10257 insert	-
pIJ10257_Rv	CAAACGGCATTGAGCGTCAGC		-
SalbPP_Ser3CysRV	GTTCTCGACGAGGGCGTTGGAGTGGGTGGCGCAGAGGTGGGCCAGCTGGCCCGGGTCGGCGAT	Core peptide mutation	
Oligo100	AAGTCGCGGTCGGAACCGTATTGCAGCAGCTTTATCATCTGCCGCTGGACGGCGCACAAATCGCGCTTAA		
SalbPPmut_FWScr	GCCGAAGTGCTGGGTGTCGAG	Screening core peptide mutation	
SalbPPmut_RVScr	GTGCCGGTGTCGAGGTGCAG		

Table S7 Accurate masses of streptamidine and related compounds.

Description	Formula	Calc. $[M+H]^+$	Obs. *m/z*	Error (ppm)
Streptamidine (HLSATH amidine)	$C_{28}H_{42}N_{10}O_8$	324.1666 ($[M+2H]^{2+}$)	324.1666	0.00
		647.3260	647.3251	1.39
Predicted HLSAT amidine	$C_{22}H_{35}N_7O_7$	510.2671	510.2668	0.59
Predicted HLSA amidine	$C_{18}H_{28}N_6O_5$	409.2194	409.2195	-0.24
Predicted acetylated and dehydrated LSA (peptide A)	$C_{14}H_{23}N_3O_5$	314.1710	314.1711	-0.32
Predicted dehydrated LSA (peptide B)	$C_{12}H_{21}N_3O_4$	272.1604	272.1595	3.31
Acetylated LSA (peptide C)	$C_{14}H_{25}N_3O_6$	354.1636 ($[M+Na]^+$)	354.1636	0.00

Table S8 Proteins encoded in the streptamidine biosynthetic gene cluster.

Protein Name	Locus tag[a] NC_020990.1/ CP004370.1	Protein ID[a] NC_020990.1/ CP004370.1	Pfam domain	Predicted function/domain	Size (AA)
AmiX	XNR_RS28595 XNR_5767	WP_106428983.1 AGI92072.1	No Pfam match	Oxidoreductase	410[b]
AmiF1	XNR_RS28600 XNR_5768	WP_015508294.1 AGI92073.1	PF01497	Iron transporter complex	329
AmiF2	XNR_RS28605 XNR_5769	WP_003946623.1 AGI92074.1	PF01032	Iron transporter complex	344
AmiF3	XNR_RS28610 XNR_5770	WP_015508295.1 AGI92075.1	PF01032	Iron transporter complex	351
AmiF4	XNR_RS28615 XNR_5771	WP_003946621.1 AGI92076.1	PF00005	Iron transporter complex	278
AmiA[c]	XNR_5772[c]	AGI92077.1[c]	No Pfam match	Precursor peptide	44
AmiB	XNR_RS28620 XNR_5773	WP_015508297.1 AGI92078.1	No Pfam match	E1-like protein	281
AmiC	XNR_RS28625 XNR_5774	WP_015508298.1 AGI92079.1	PF02129	Hydrolase	508
AmiD	XNR_RS28630 XNR_5775	WP_008409979.1 AGI92080.1	PF02624	YcaO-domain	455
AmiE	XNR_RS28635 XNR_5776	WP_015508299.1 AGI92081.1	No Pfam match	Dehydrogenase	435
AmiT1	XNR_RS28640 XNR_5777	WP_003946615.1 AGI92082.1	PF00005	ABC transporter	581
AmiT2	XNR_RS28645 XNR_5778	WP_015508300.1 AGI92083.1	PF00005	ABC transporter	594

[a.] Locus tags and protein IDs are provided both for the NCBI RefSeq accession (NC_020990.1) and the GenBank accession (CP004370.1) of the *S. albidoflabus* J1074 genome sequence.
[b.] AGI92072.1/XNR_5767 annotated as a 417 AA protein.
[c.] Precursor peptide AmiA is not annotated in the RefSeq accession (NC_020990.1).

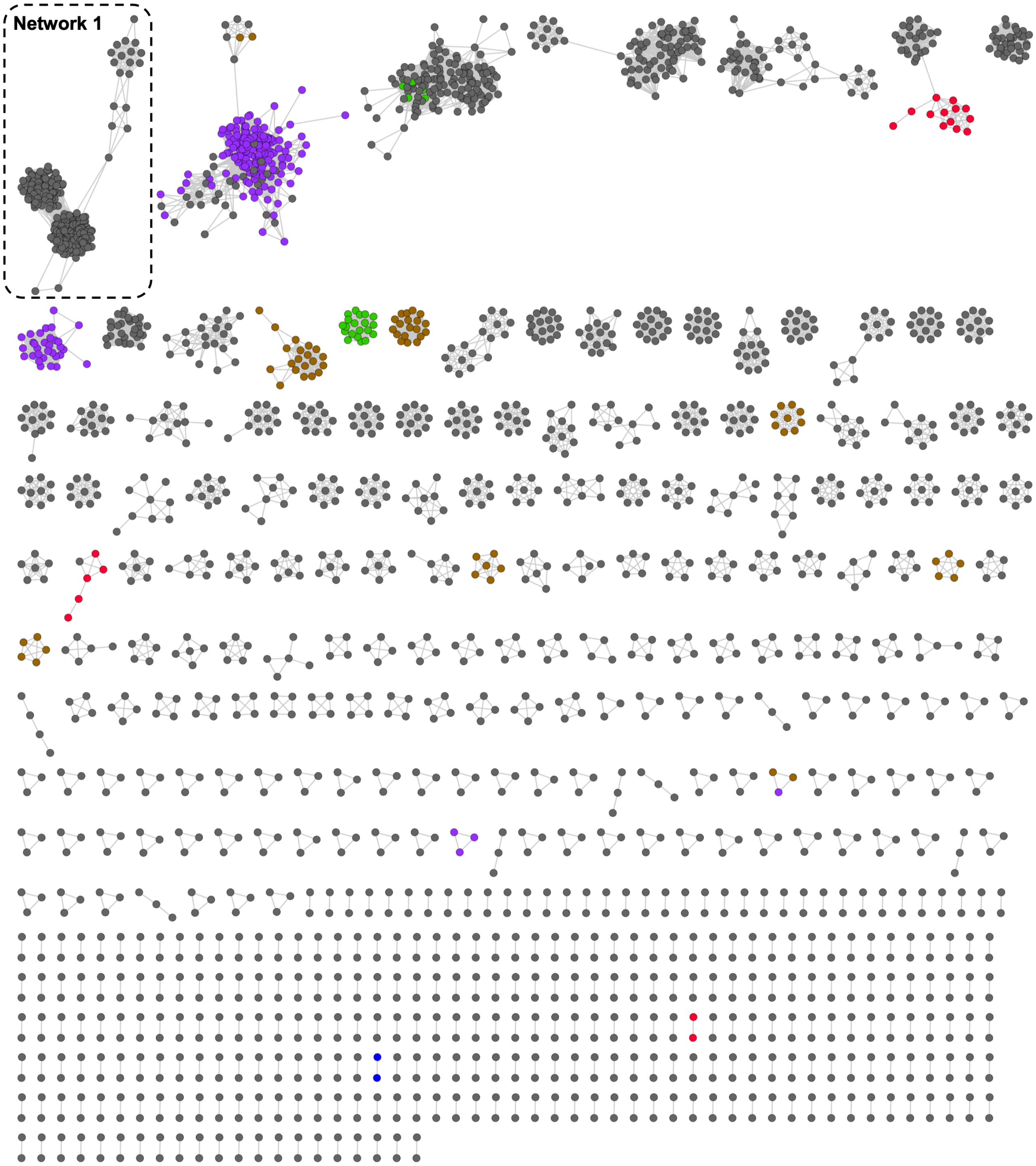

Figure S1 Precursor peptide networks associated with Actinobacterial YcaO proteins. Precursors with homology to known RiPPs are highlighted: green = bottromycin family (NCBI HMM domain NF033414), brown = thiazolylpeptide families (NF033400 and NF033399), red = thioviridamide family (NF033415), purple = thiocillin families (NF033482 and NF033401), dark blue = lasso peptide family (NF033521).

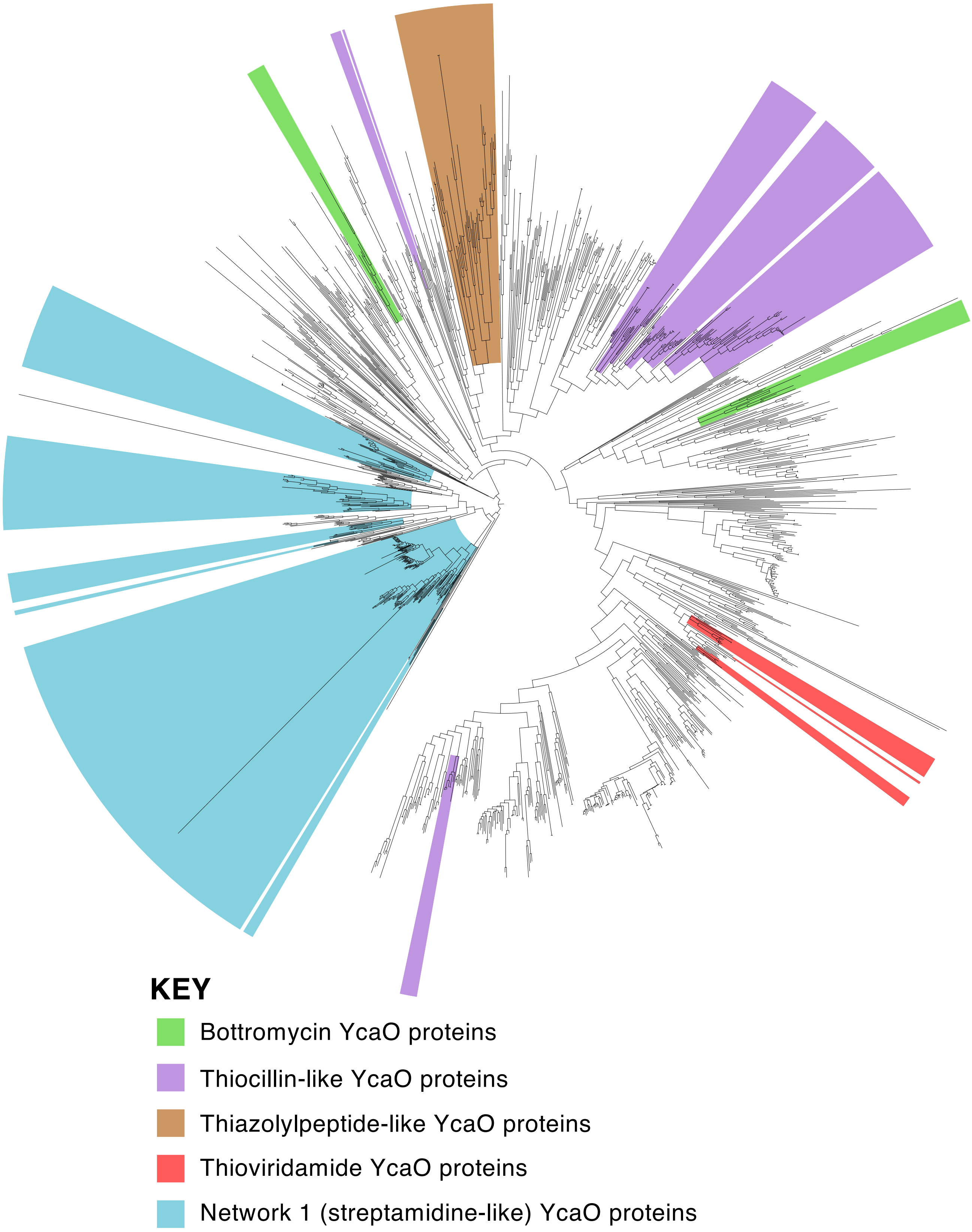

Figure S2 Version of the actinobacterial YcaO maximum likelihood tree shown in Figure 2 with branch distances included. A single colour (blue) is used to highlight all YcaO protein clades associated with Network 1 peptides. Figure generated using iTOL[7].

Figure S3 (shown below) Alignment of precursor peptides from network 1 containing motif A. Sequences were aligned using ClustalW[12] and visualised using Jalview[30] with Taylor colour-coding[31]. The figure extends to the following page, which includes the consensus sequence for these Motif A peptides with the streptamidine core peptide region highlighted.

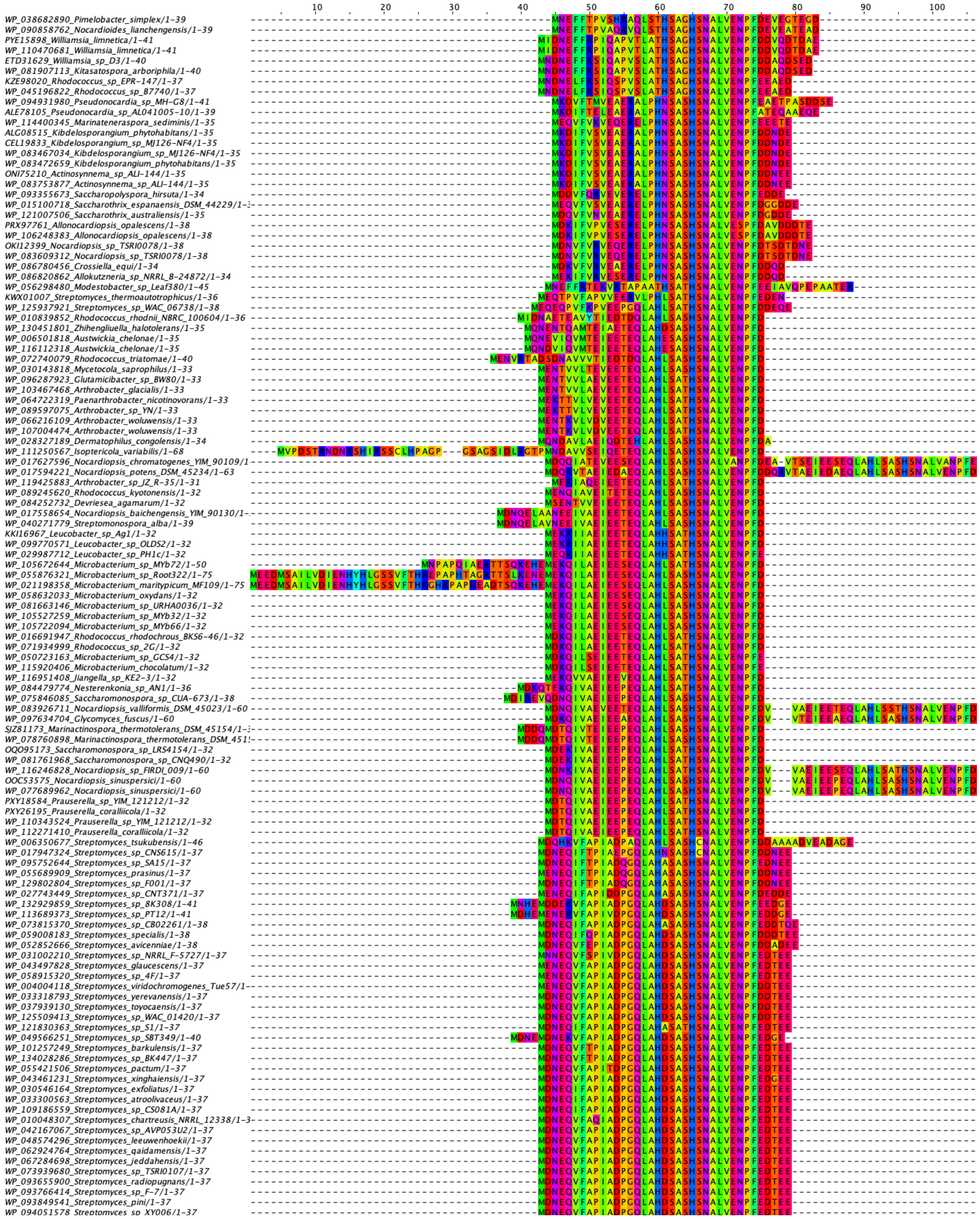

Figure S3 continued:

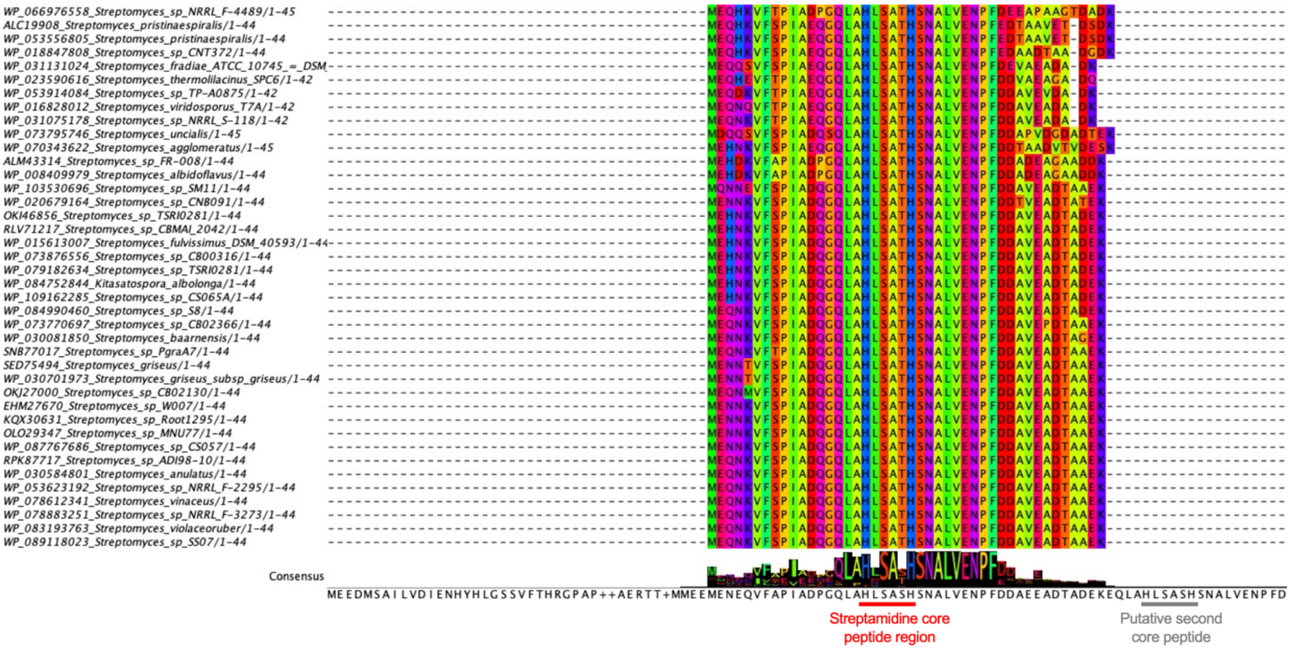

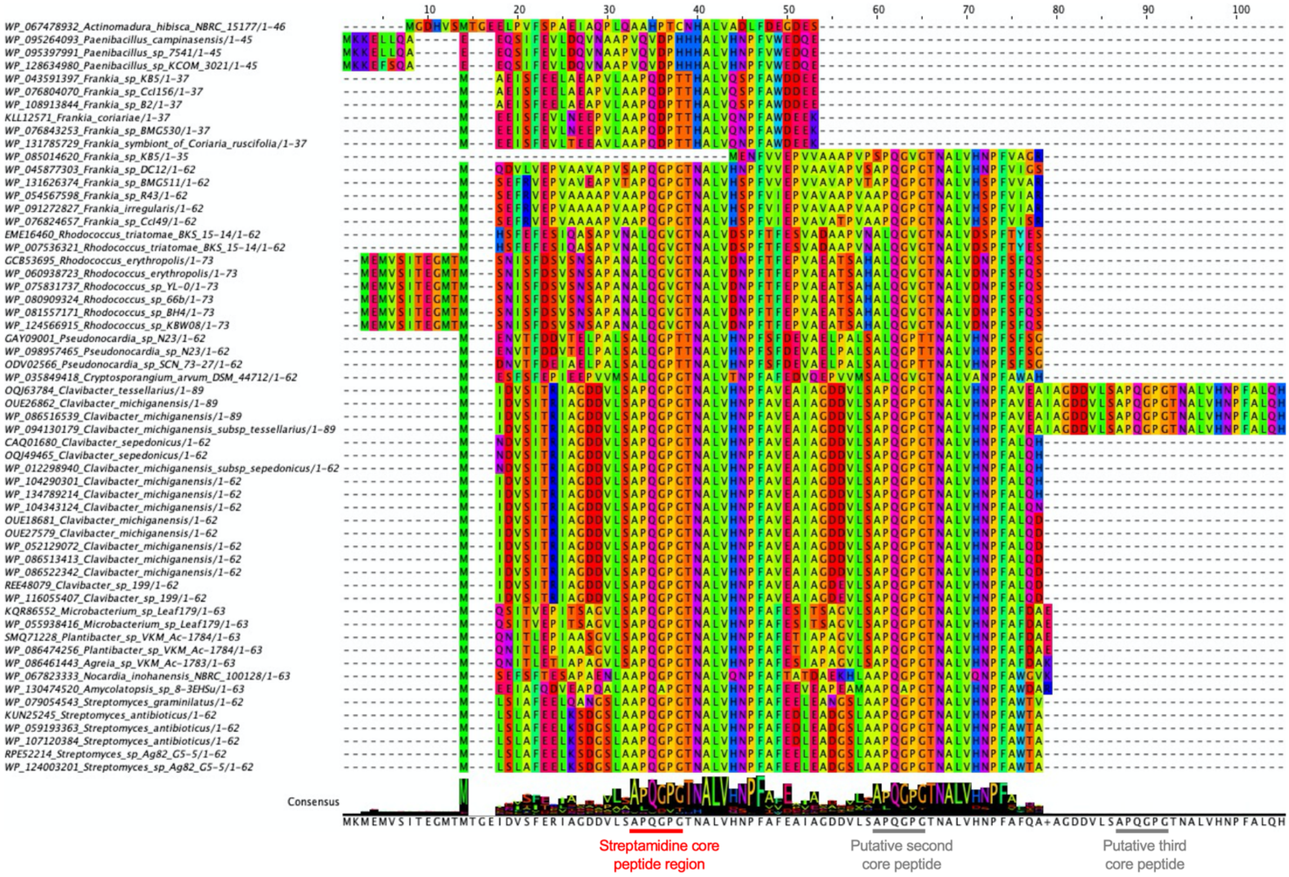

Figure S4 Alignment of precursor peptides from network 1 containing motif B. Sequences were aligned using ClustalW[12] and visualised using Jalview[30] with Taylor colour-coding[31]. The consensus sequence for these Motif B peptides is shown with the putative streptamidine core peptide region(s) highlighted based on their proximity to the ALV motif.

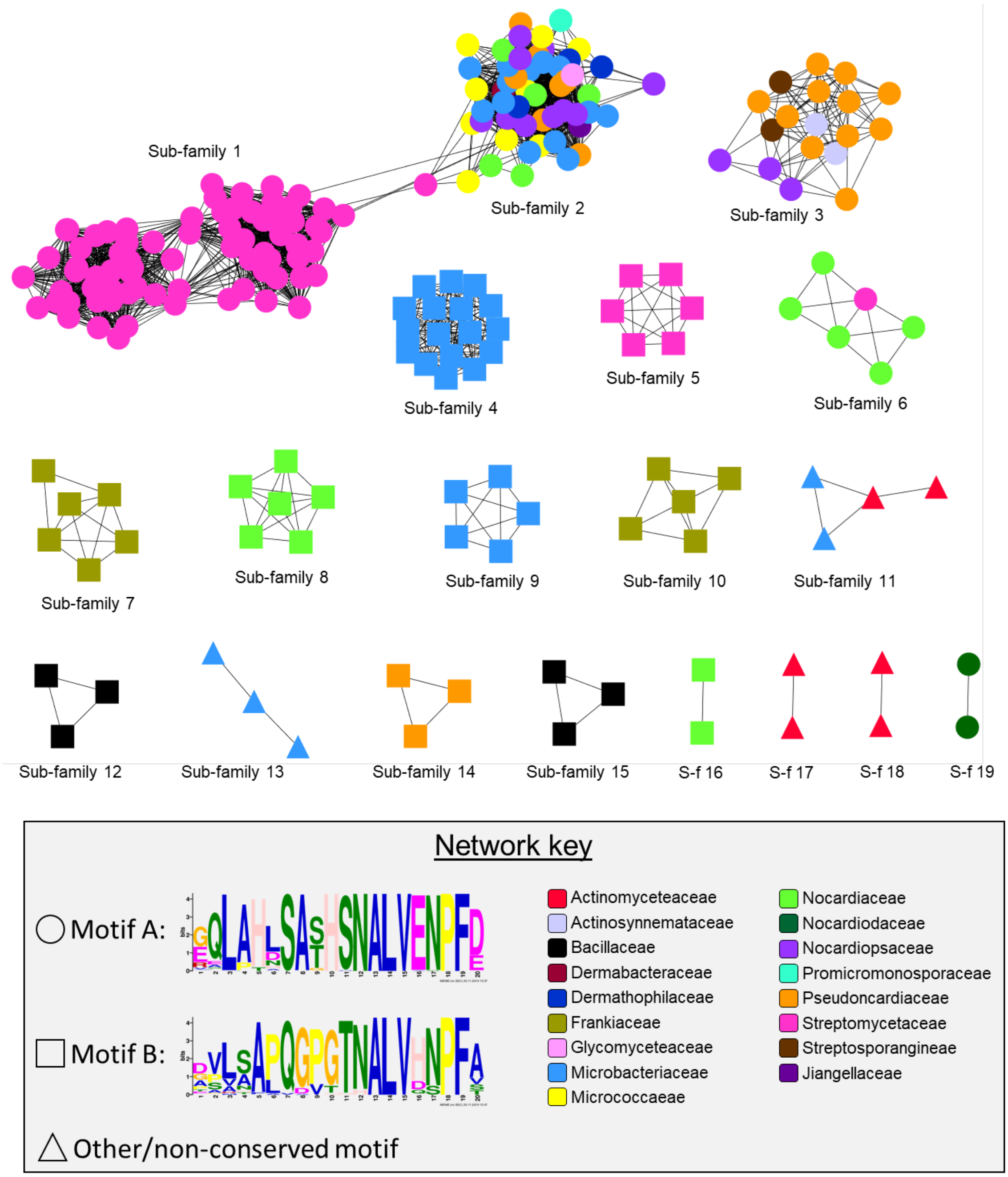

Figure S5 Network analysis of the 237 peptide sequences identified with RiPPER (network 1 from Figure 1 and Figure S1) using an identity cut-off of 80%. Similar sequences are clustered together in sub-families and nodes are colour coded by the associated bacterial family. Peptides containing motif A are indicated with a circular node and peptides containing motif B are indicated by a square node. Precursors containing neither motif are indicated by a triangular node. Motifs A and B were identified by MEME[14] analysis of the 237 sequences. Each motif appears once, twice or three times in 208 of the precursor sequences.

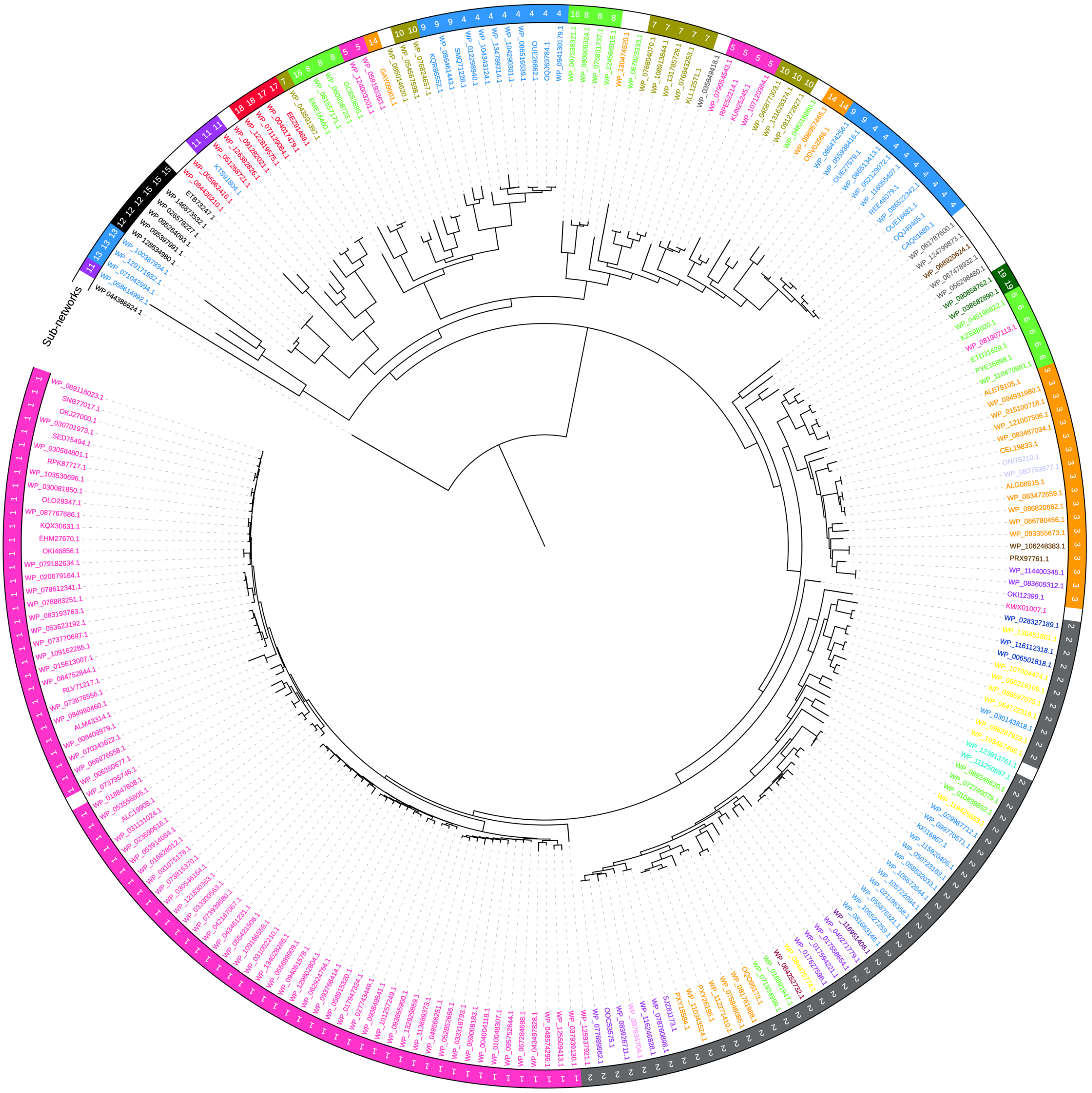

Figure S6 Maximum likelihood tree of YcaO proteins associated with network 1 of putative precursor peptides. The tree is rooted using WP_044386624.1 as an outgroup. Precursor peptide sub-networks (80% identity networks from Figure S5) are mapped as an outer colour strip that is labelled with the sub-network number. This is colour-coded according to the dominant bacterial family within each sub-network (colour coding as in Figure S5), with the exception of sub-network 2, which consists of diverse bacterial families. Tree node labels are colour-coded by bacterial family. This shows that precursor peptides appear to have co-evolved with their cognate YcaO. Note that a small number of peptides do not network with the 80% identity cut-off and are therefore unlabelled on the colour strip. Figure generated using iTOL[7].

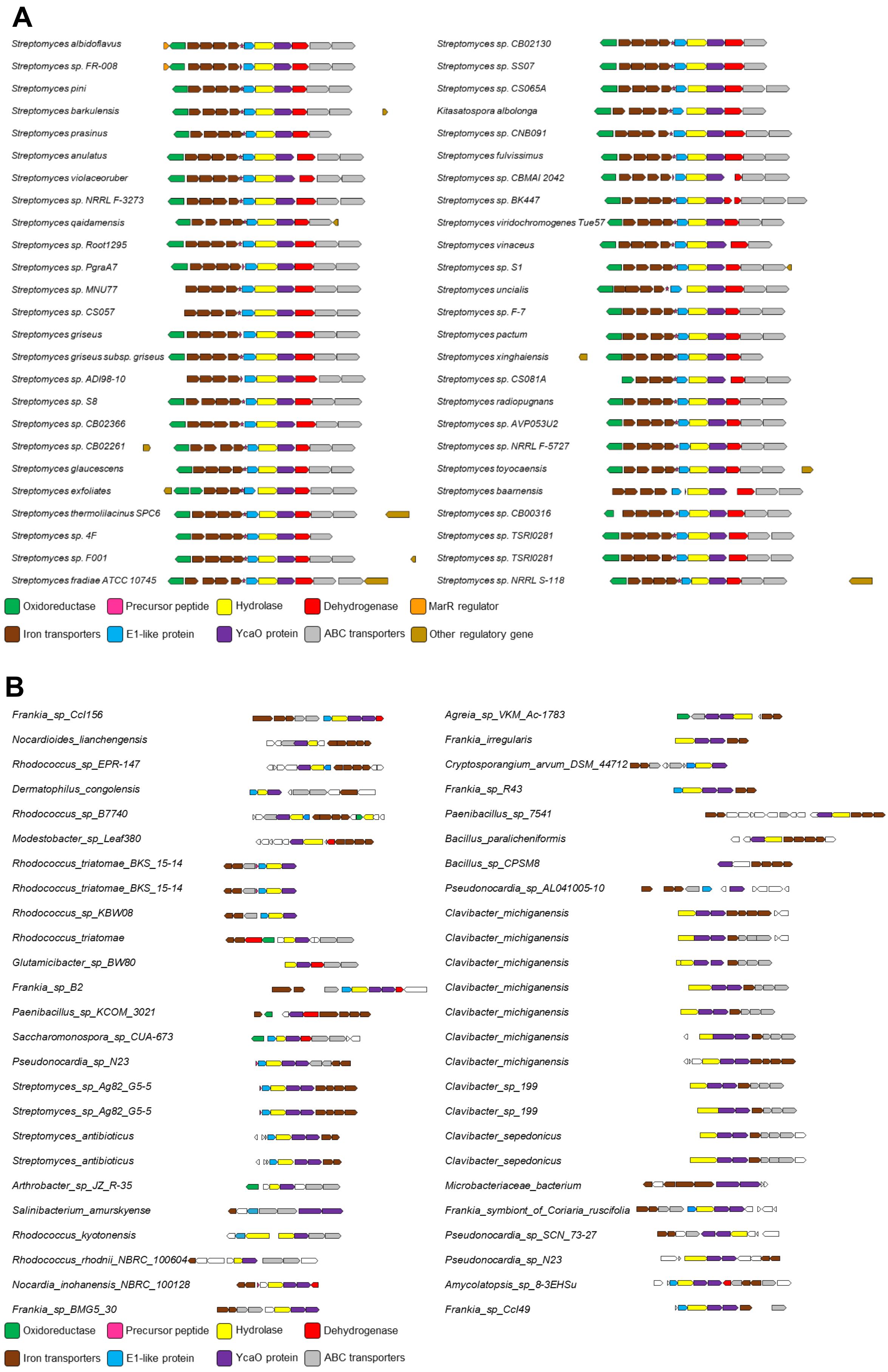
A
Streptomyces albidoflavus
Streptomyces sp. FR-008
Streptomyces pini
Streptomyces barkulensis
Streptomyces prasinus
Streptomyces anulatus
Streptomyces violaceoruber
Streptomyces sp. NRRL F-3273
Streptomyces qaidamensis
Streptomyces sp. Root1295
Streptomyces sp. PgraA7
Streptomyces sp. MNU77
Streptomyces sp. CS057
Streptomyces griseus
Streptomyces griseus subsp. griseus
Streptomyces sp. ADI98-10
Streptomyces sp. S8
Streptomyces sp. CB02366
Streptomyces sp. CB02261
Streptomyces glaucescens
Streptomyces exfoliates
Streptomyces thermolilacinus SPC6
Streptomyces sp. 4F
Streptomyces sp. F001
Streptomyces fradiae ATCC 10745
Streptomyces sp. CB02130
Streptomyces sp. SS07
Streptomyces sp. CS065A
Kitasatospora albolonga
Streptomyces sp. CNB091
Streptomyces fulvissimus
Streptomyces sp. CBMAI 2042
Streptomyces sp. BK447
Streptomyces viridochromogenes Tue57
Streptomyces vinaceus
Streptomyces sp. S1
Streptomyces uncialis
Streptomyces sp. F-7
Streptomyces pactum
Streptomyces xinghaiensis
Streptomyces sp. CS081A
Streptomyces radiopugnans
Streptomyces sp. AVP053U2
Streptomyces sp. NRRL F-5727
Streptomyces toyocaensis
Streptomyces baarnensis
Streptomyces sp. CB00316
Streptomyces sp. TSRI0281
Streptomyces sp. TSRI0281
Streptomyces sp. NRRL S-118
Oxidoreductase
Precursor peptide
Hydrolase
Dehydrogenase
MarR regulator
Iron transporters
E1-like protein
YcaO protein
ABC transporters
Other regulatory gene
B
Frankia_sp_CcI156
Nocardioides_lianchengensis
Rhodococcus_sp_EPR-147
Dermatophilus_congolensis
Rhodococcus_sp_B7740
Modestobacter_sp_Leaf380
Rhodococcus_triatomae_BKS_15-14
Rhodococcus_triatomae_BKS_15-14
Rhodococcus_sp_KBW08
Rhodococcus_triatomae
Glutamicibacter_sp_BW80
Frankia_sp_B2
Paenibacillus_sp_KCOM_3021
Saccharomonospora_sp_CUA-673
Pseudonocardia_sp_N23
Streptomyces_sp_Ag82_G5-5
Streptomyces_sp_Ag82_G5-5
Streptomyces_antibioticus
Streptomyces_antibioticus
Arthrobacter_sp_JZ_R-35
Salinibacterium_amurskyense
Rhodococcus_kyotonensis
Rhodococcus_rhodnii_NBRC_100604
Nocardia_inohanensis_NBRC_100128
Frankia_sp_BMG5_30
Agreia_sp_VKM_Ac-1783
Frankia_irregularis
Cryptosporangium_arvum_DSM_44712
Frankia_sp_R43
Paenibacillus_sp_7541
Bacillus_paralicheniformis
Bacillus_sp_CPSM8
Pseudonocardia_sp_AL041005-10
Clavibacter_michiganensis
Clavibacter_michiganensis
Clavibacter_michiganensis
Clavibacter_michiganensis
Clavibacter_michiganensis
Clavibacter_michiganensis
Clavibacter_michiganensis
Clavibacter_sp_199
Clavibacter_sp_199
Clavibacter_sepedonicus
Clavibacter_sepedonicus
Microbacteriaceae_bacterium
Frankia_symbiont_of_Coriaria_ruscifolia
Pseudonocardia_sp_SCN_73-27
Pseudonocardia_sp_N23
Amycolatopsis_sp_8-3EHSu
Frankia_sp_CcI49
Oxidoreductase
Precursor peptide
Hydrolase
Dehydrogenase
Iron transporters
E1-like protein
YcaO protein
ABC transporters

Figure S7 (previous page) A. First 50 examples of homologous gene clusters identified by MultiGeneBlast[17] analysis of the *S. albidoflavus* BGC. The core set of putative biosynthetic genes are present in all examples, which are predominantly from the *Streptomyces* genus and are associated with precursor peptides containing motif A. Surrounding genes that do not have predicted biosynthetic function within the RiPP biosynthetic pathway have been omitted, with the exception of genes annotated as having a regulatory function. B. BGCs 151-200 identified from MultiGeneBlast analysis of the *S. albidoflavus* BGC. These BGC examples are distinct from the first 50 examples, as many encode two YcaO proteins, additional hypothetical proteins (in white), and generally lack the *amiE*, *amiB* and *amiX* genes. Additionally, this subset of BGCs is usually associated with precursor peptides containing motif B.

A

Ion *m/z*	RT / min	WT1	WT2	WT3	WT4	Mutant1	Mutant2	Mutant3	Mutant4
324.17	2.58	1628189	1895000	2314187	2466265	0	0	136742	158062
290.16	0.92	1783434	2104341	1970184	2186368	154724	0	0	0
324.67	1.74	1301830	1929863	2040364	1981539	0	0	0	0
324.67	2.59	809435	1254146	1371572	1559192	0	0	0	0
338.18	1.35	959988	1359900	989904	994114	0	0	0	131368
320.17	0.88	491922	553274	574435	585459	0	0	0	0
647.33	1.74	372300	544327	573868	484952	0	0	0	0
647.33	2.60	305683	460086	454254	527232	0	0	0	0
251.15	0.91	291034	353716	375759	330858	0	0	0	0
510.27	2.52	267676	309829	307114	259442	0	0	0	0
290.16	1.79	190600	242783	300188	303723	0	0	0	0
320.17	1.34	197786	251664	229483	225336	0	0	0	0
329.17	2.69	187811	285428	160111	188850	0	0	0	0
315.17	1.74	147017	179590	175624	143522	0	0	0	0
290.16	2.56	117759	182052	168519	151214	0	0	0	0
356.18	1.09	151608	165779	122061	155647	0	0	0	0
324.67	2.83	121792	118528	121152	154880	0	0	0	0
359.21	2.12	135010	113220	120006	120660	0	0	0	0

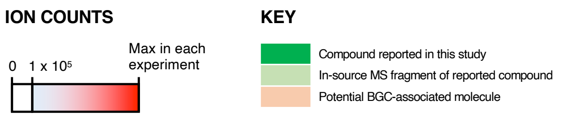

B

Ion *m/z*	RT / min	SalbC1	SalbC2	Δ*amiA1*	Δ*amiA2*	Δ*amiA3*	Δ*amiB1*	Δ*amiB2*	Δ*amiB3*	Δ*amiC1*	Δ*amiC2*	Δ*amiC3*	Δ*amiD1*	Δ*amiD2*	Δ*amiD3*	Δ*amiE1*	Δ*amiE2*	Δ*amiE3*	Δ*amiX1*	Δ*amiX2*	Δ*amiX3*
314.17	3.03	1042037	847530	0	0	0	0	0	0	0	0	0	0	0	0	1605224	2048790	1588020	2825272	2569195	2451654
272.16	2.98	387710	410005	0	0	0	0	0	0	0	0	0	0	0	0	796578	1351619	943842	1570774	1699116	1560029
332.18	3.02	393459	329963	0	0	0	0	0	0	0	0	0	0	0	0	887866	1116939	712023	1980880	1813192	1386092
324.17	1.40	2262279	2214873	0	0	0	0	0	0	0	0	0	0	0	0	0	0	0	1057241	1172300	1139022
268.16	3.02	317850	255269	0	0	0	0	0	0	0	0	0	0	0	0	542292	723234	471336	1355002	1150870	981815
510.27	1.55	1475999	1171449	0	0	0	0	0	0	0	0	0	0	0	0	0	0	0	810404	763166	660021
354.16	3.03	280038	252484	0	0	0	0	0	0	0	0	0	0	0	0	443885	483075	389179	649966	604358	585720
252.68	2.59	0	0	0	0	0	805274	823917	750249	652656	0	0	0	0	0	0	0	0	0	0	0
731.49	5.88	0	0	0	0	0	472995	414900	387367	483298	185884	593732	0	0	0	0	0	0	0	0	0
534.28	0.94	0	0	0	0	0	535754	340404	311922	409939	462929	279795	0	0	0	0	0	0	0	0	0
282.18	2.79	0	0	0	0	0	480684	308963	334050	426267	187481	358679	0	0	0	0	0	0	0	0	0
301.71	2.68	0	113920	0	0	0	0	0	0	0	0	0	0	0	0	528479	618234	813954	0	0	0
647.32	1.39	544662	545338	0	0	0	0	0	0	0	0	0	0	0	0	0	0	0	290000	237703	223171
753.48	5.89	0	0	0	0	0	330045	334540	286914	278207	135959	390631	0	0	0	0	0	0	0	0	0
414.69	1.63	498706	567594	0	0	0	0	0	0	0	0	0	0	0	0	0	0	0	179712	206891	210799
572.27	2.91	0	220338	0	0	0	0	0	0	309065	0	0	240054	235837	200915	0	207047	0	0	0	206498

C

Ion *m/z*	RT / min	SalbC1	SalbC2	SalbC3	Δ*amiA1*	Δ*amiA2*	Δ*amiA3*	Δ*amiF1*	Δ*amiF2*	Δ*amiF3*	Δ*amiT1*	Δ*amiT2*	Δ*oxy1*	Δ*oxy2*	Δ*marR1*	Δ*marR2*	Δ*marR3*	Δ*metred1*	Δ*metred2*	Δ*metred3*	Δ*acylT1*	Δ*acylT2*
324.17	1.34	2262502	2715880	2252375	0	0	0	0	0	0	389484	206483	2638215	3555376	3931198	2664038	4194032	3046506	2325230	2642271	2608474	129689
324.67	1.34	1480698	1563640	1455540	0	0	0	0	0	0	134003	0	1586077	2033893	2325951	1784058	2506411	1742056	1510709	1510224	1693574	0
414.69	1.58	1181609	1645592	1047319	0	0	0	0	0	0	363852	290531	1451534	1318812	1240448	1324889	1337093	1704719	1659297	1820455	809350	379525
415.19	1.58	647608	973697	518697	0	0	0	0	0	0	204596	150095	714917	663617	595986	641513	867811	791543	1048942	944463	519469	208578
405.69	1.94	204544	473083	449510	0	0	0	0	0	0	279087	349649	488694	521578	724514	443171	529039	575600	634771	456094	421526	0
607.34	4.90	0	0	0	0	0	0	0	0	0	661060	585094	405660	453464	0	0	0	0	0	0	1448119	1570230
647.32	1.34	513445	744674	437623	0	0	0	0	0	0	0	0	447968	462424	582933	555897	579071	508534	523511	408668	407028	0
405.69	1.58	279266	392827	179786	0	0	0	0	0	0	0	0	316305	346105	290873	320717	341332	416925	564351	383123	202526	0
272.16	2.92	257884	387278	284473	0	0	0	0	0	0	338726	253540	227157	309806	327270	286979	364493	179404	253616	202020	112176	0
510.27	1.50	104359	294043	110043	0	0	0	0	0	0	0	0	167448	222399	1113021	268230	524694	158387	126083	131314	300238	0
406.19	1.94	203053	238427	179119	0	0	0	0	0	0	190795	189693	272956	274319	350573	235753	298792	304645	302031	198489	166590	0
290.16	1.32	197753	216467	152115	0	0	0	0	0	0	0	0	177502	227549	310417	202821	348978	224991	167021	185888	216711	0
855.45	5.88	0	0	0	0	0	0	139416	270140	299096	119115	157407	0	0	0	0	0	0	0	0	0	243550
843.38	4.41	160391	143875	116861	0	0	0	119398	128183	195715	128757	149272	0	0	0	0	0	127475	166995	126728	0	157425
619.33	3.63	0	0	0	0	0	0	765322	752744	691650	0	0	0	0	0	0	0	0	0	0	0	0
708.38	4.82	0	0	0	0	0	0	0	0	0	165798	291112	0	0	0	0	0	144121	0	0	0	430029
314.17	2.97	0	167284	0	0	0	0	0	0	0	0	0	0	167189	465471	237824	360018	175044	129755	137679	146284	0
251.15	1.34	227258	241712	139190	0	0	0	0	0	0	0	0	218172	289739	0	184452	0	171103	169619	138027	171547	0

Figure S8 Comparative LC-MS data acquired using a Shimadzu IT-TOF and processed using Profiling Solution (Shimadzu). Numbers reflect ion intensities as calculated using Profiling Solution and most abundant ions are shown in each panel. Columns represent independent cultures. A. *S. albidoflavus* J1074 wild type versus pathway-disrupted *S. albidoflavus* J1074 mutant. Retention times differ to other experiments due to different chromatography conditions for this analysis. B. *S. coelicolor* M1146-pCAPSalbC versus biosynthetic gene mutants: Δ*amiA*, Δ*amiB*, Δ*amiC*, Δ*amiD*, Δ*amiE* and Δ*amiX*. C. *S. coelicolor* M1146-pCAPSalbC versus versus mutants of other genes cloned in pCAPSalbC: iron transporter deletion (Δ*amiF*), ABC transporter deletion (Δ*amiT*), oxygenase deletion (Δ*oxy*), MarR deletion (Δ*marR*), peptide methionine sulfoxide reductase deletion (Δ*metred*), and acetyltransferase deletion (Δ*acylT*).

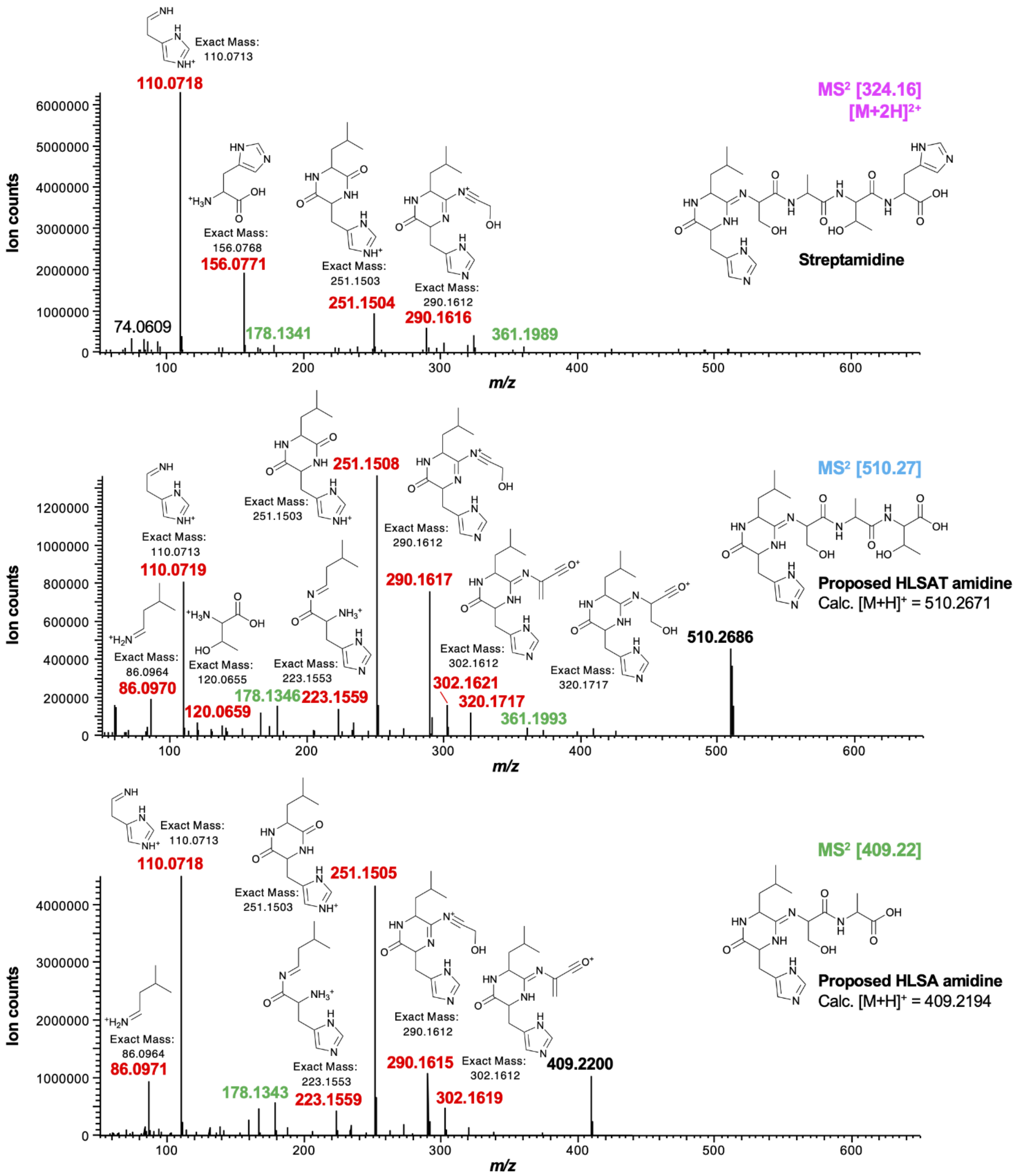

Figure S9 Accurate MS/MS fragmentation data for streptamidine (*m/z* 324.16), proposed HLSAT amidine (*m/z* 510.27) and proposed HLSA amidine (*m/z* 409.22), obtained using a Thermo Q-Exactive. Fragments with proposed structures are labelled in red, while unannotated fragments that are common to two or more spectra are labelled in green. Parent ions are labelled in black. The following fragments are common to all spectra: *m/z* 110.07, *m/z* 178.13, *m/z* 251.15, *m/z* 290.16.

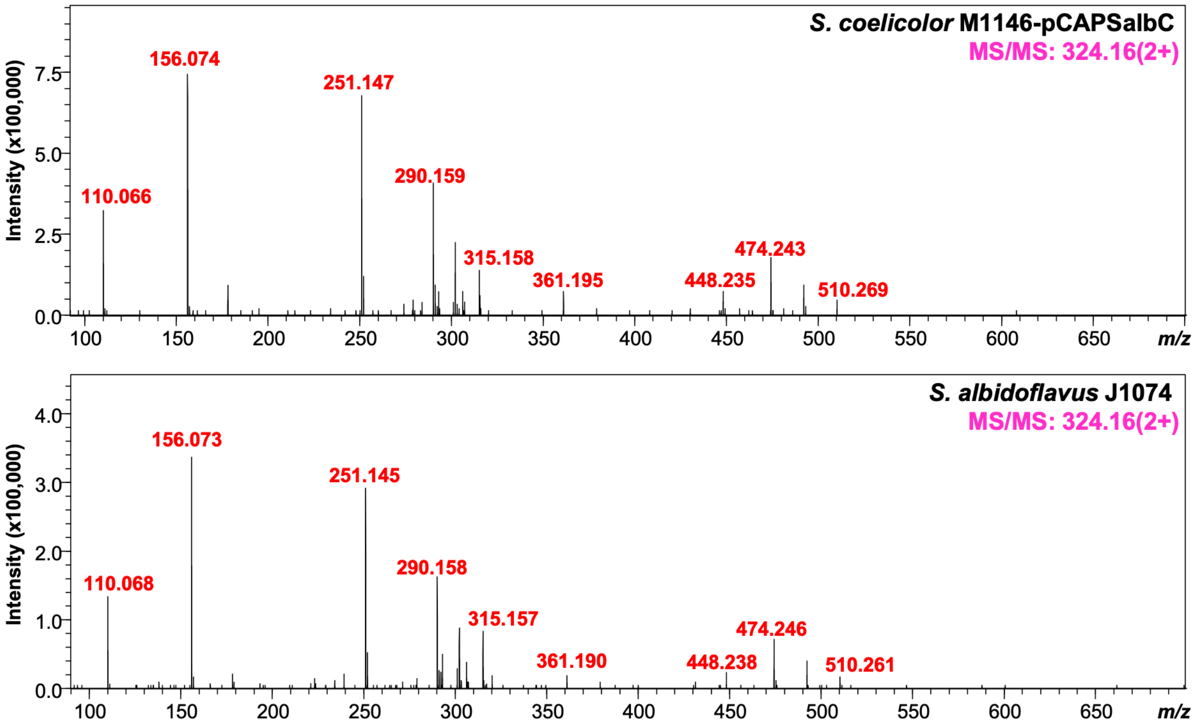

Figure S10 MS/MS fragmentation data for streptamidine (*m/z* 324.16, $[M+2H]^{2+}$) from *S. coelicolor* M1146-pCAPSalbC and *S. albidoflavus* J1074. Spectra obtained on a Shimadzu IT-TOF.

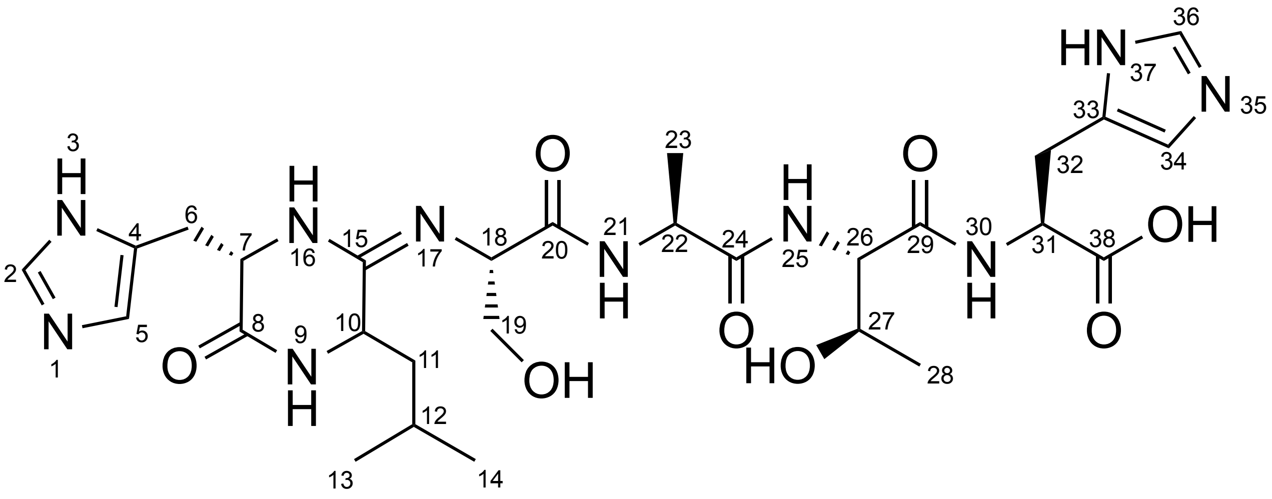

Figure S11 Structure of streptamidine with numbered atoms.

Table S9 NMR chemical shift assignments for streptamidine in DMSO-d_6.

C/N number	Amino acid	δ_C (ppm)	δ_H (ppm)	Multiplicity	Coupling constant (Hz)
1	His1	--	--	--	
2	His1	134.7	7.48	s	
3	His1	--			
4	His1	131.6[a]	--	--	
5	His1	121.0[b]	6.67	s	
6	His1	32.6	2.84<br>2.75	dd<br>dd	$J_{6a,6b}$ = 14.1, $J_{6a,7}$ = 5.2<br>$J_{6a,6b}$ = 14.1, $J_{6b,7}$ = 7.4
7	His1	58.8	3.95-3.91	m	
8	His1	170.7	--	--	
9	Leu2	--	8.05	s	
10	Leu2	51.0	3.82	d	$J_{10,11a}$ = 10.8
11	Leu2	45.9	1.41-1.35, 0.77-0.71	m, m	
12	Leu2	23.8	1.64-1.57	m	
13	Leu2	21.4	0.82	d	$J_{12,13}$ = 6.4
14	Leu2	24.1	0.78	d	$J_{12,14}$ = 6.6
15	Leu2	157.1	--	--	
16	His1	--			
17	Ser3	--	--	--	
18	Ser3	57.1	4.30-4.27	m	
19	Ser3	62.2	3.62	d	$J_{18,19}$ = 5.9
20	Ser3	171.4	--	--	
21	Ala4	--	8.09	d	$J_{21,22}$ = 7.0
22	Ala4	48.9	4.41-4.35	m	
23	Ala4	18.7	1.25	d	$J_{22,23}$ = 7.1
24	Ala4	172.9	--	--	
25	Thr5	--	7.88	d	$J_{25,26}$ = 8.6
26	Thr5	59.3	4.14	dd	$J_{25,26}$ = 8.6, $J_{26,27}$ = 3.6
27	Thr5	66.9	4.02-3.98	m	
28	Thr5	19.9	1.00	d	$J_{27,28}$ = 6.3
29	Thr5	170.0	--	--	
30	His6	--	7.78	d	$J_{30,31}$ = 7.5
31	His6	53.5	4.27-4.24	m	
32	His6	29.2	2.97<br>2.82	dd<br>dd	$J_{32a,32b}$ = 14.8, $J_{31,32a}$ = 5.1<br>$J_{32a,32b}$ = 14.8, $J_{31,32b}$ = 7.4
33	His6	134.1[a]	--	--	
34	His6	117.1[b]	6.81	s	
35	His6	--	--	--	
36	His6	135.1	7.54	s	
37	His6	--		--	
38	His6	173.1	--	--	

a. ^{13}C chemical shifts for His1(4) and His6(33) were obtained from HMBC, and match literature values expected for corresponding carbons in the histidine ring[32]

b. ^{13}C chemical shifts for His1(5) and His6(34) were obtained from HMBC and HSQCed spectra, and match literature values expected for corresponding carbons in the histidine ring[32]

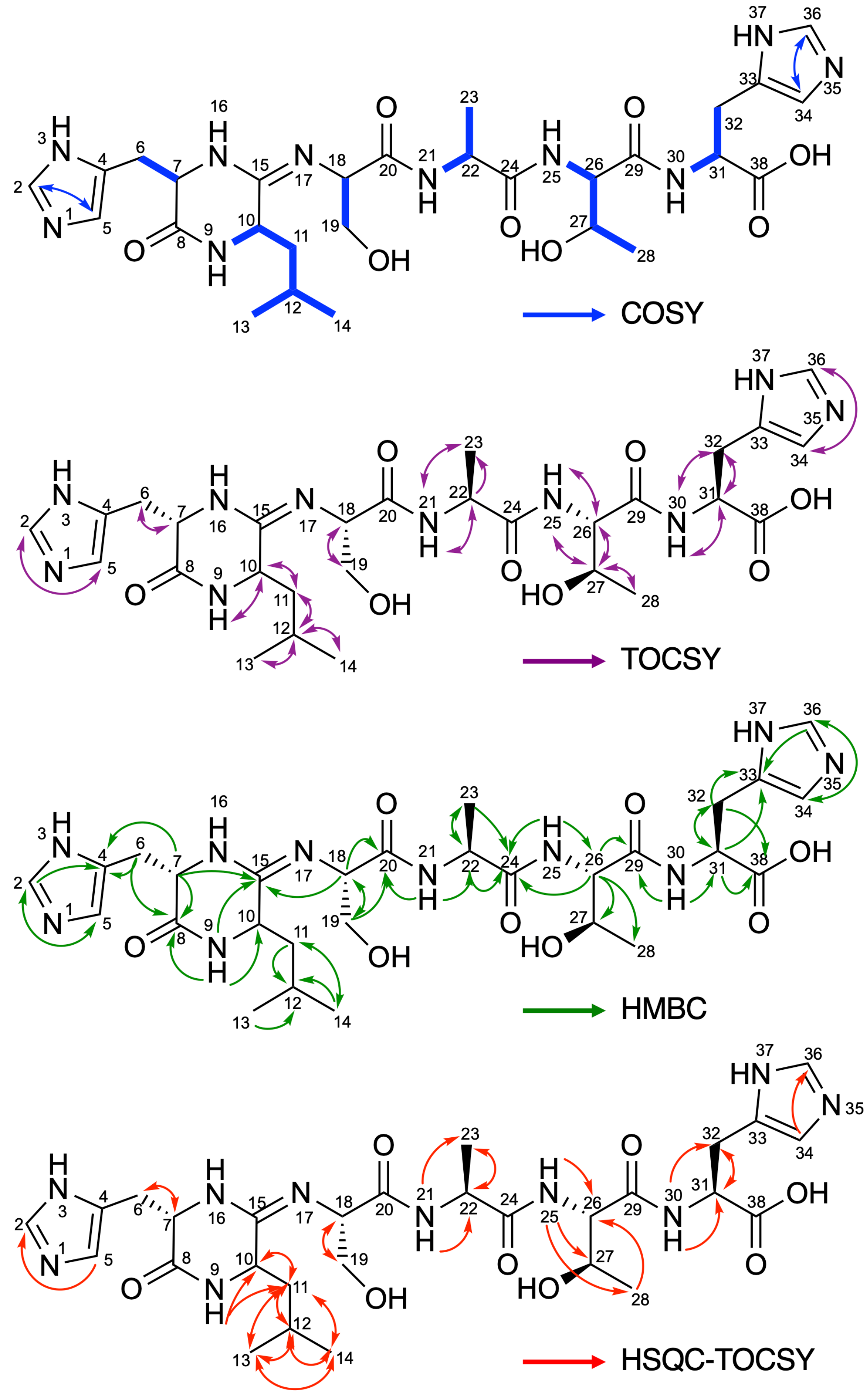

Figure S12 NMR correlation data observed in 2D spectra (Figures S15 - S20). Double-headed arrows indicate where mutual correlations were observed; single-headed arrows to indicate where one-way correlations were observed (H to C).

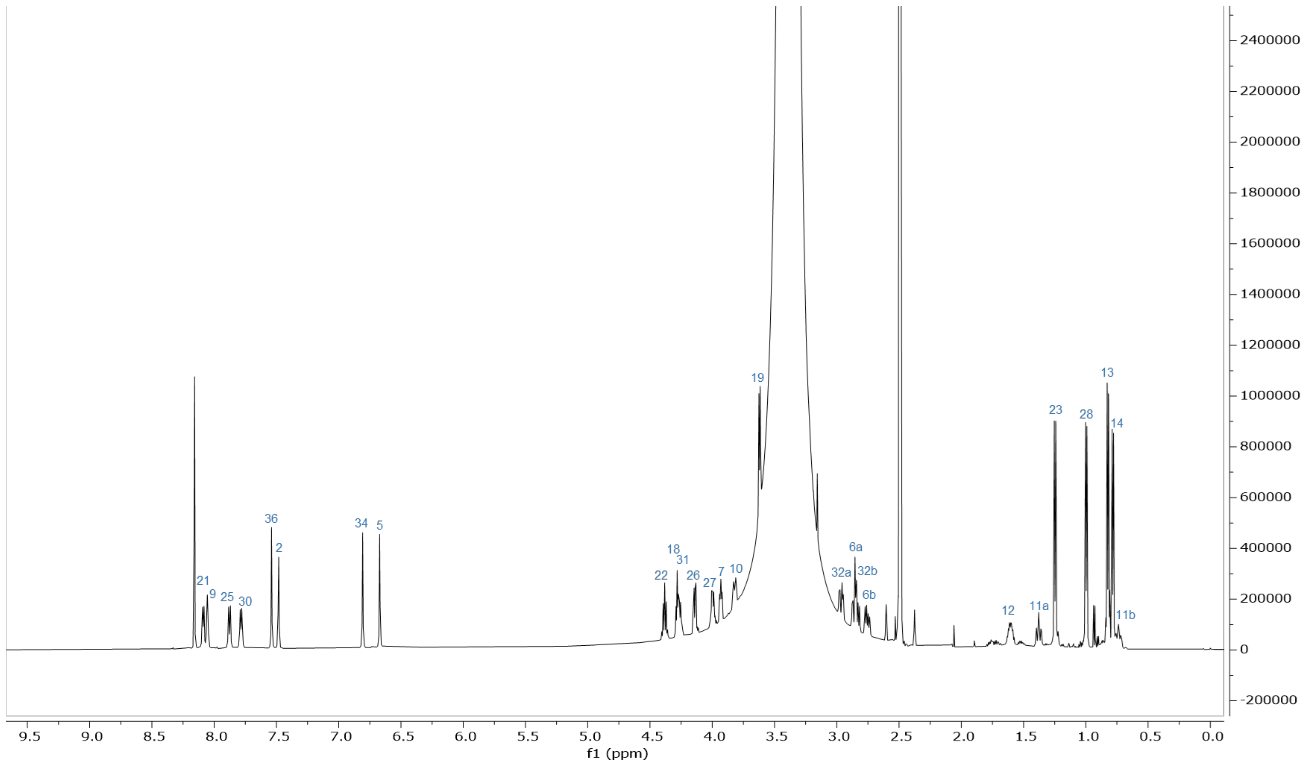

Figure S13 ^{1}H NMR spectrum (600 MHz, DMSO-d_6, 298 K) of streptamidine.

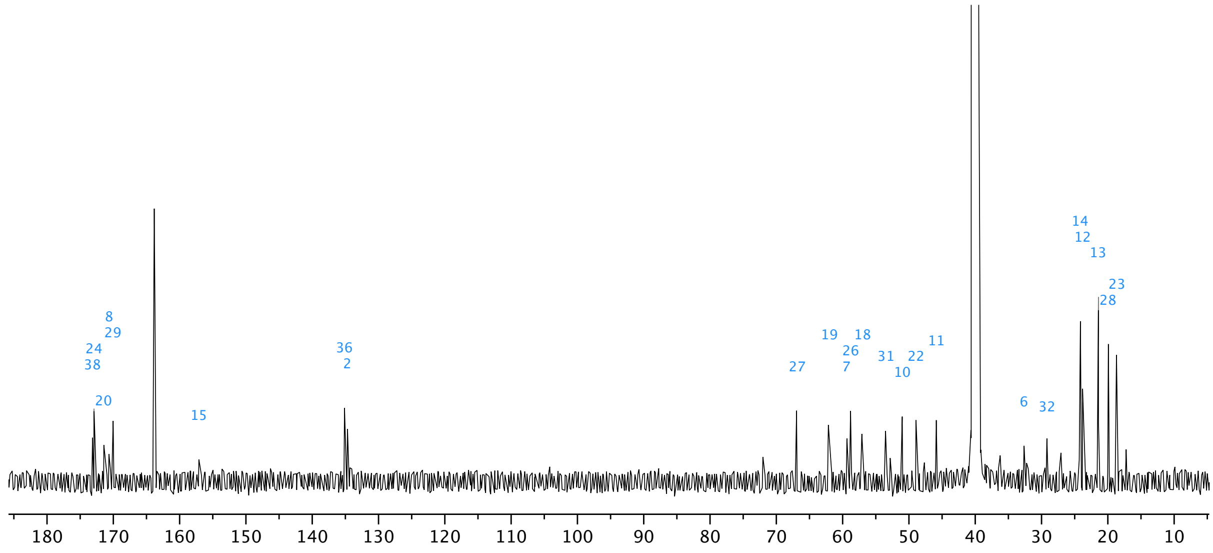

Figure S14 ^{13}C NMR spectrum (150 MHz, DMSO-d_6, 298 K) of streptamidine.

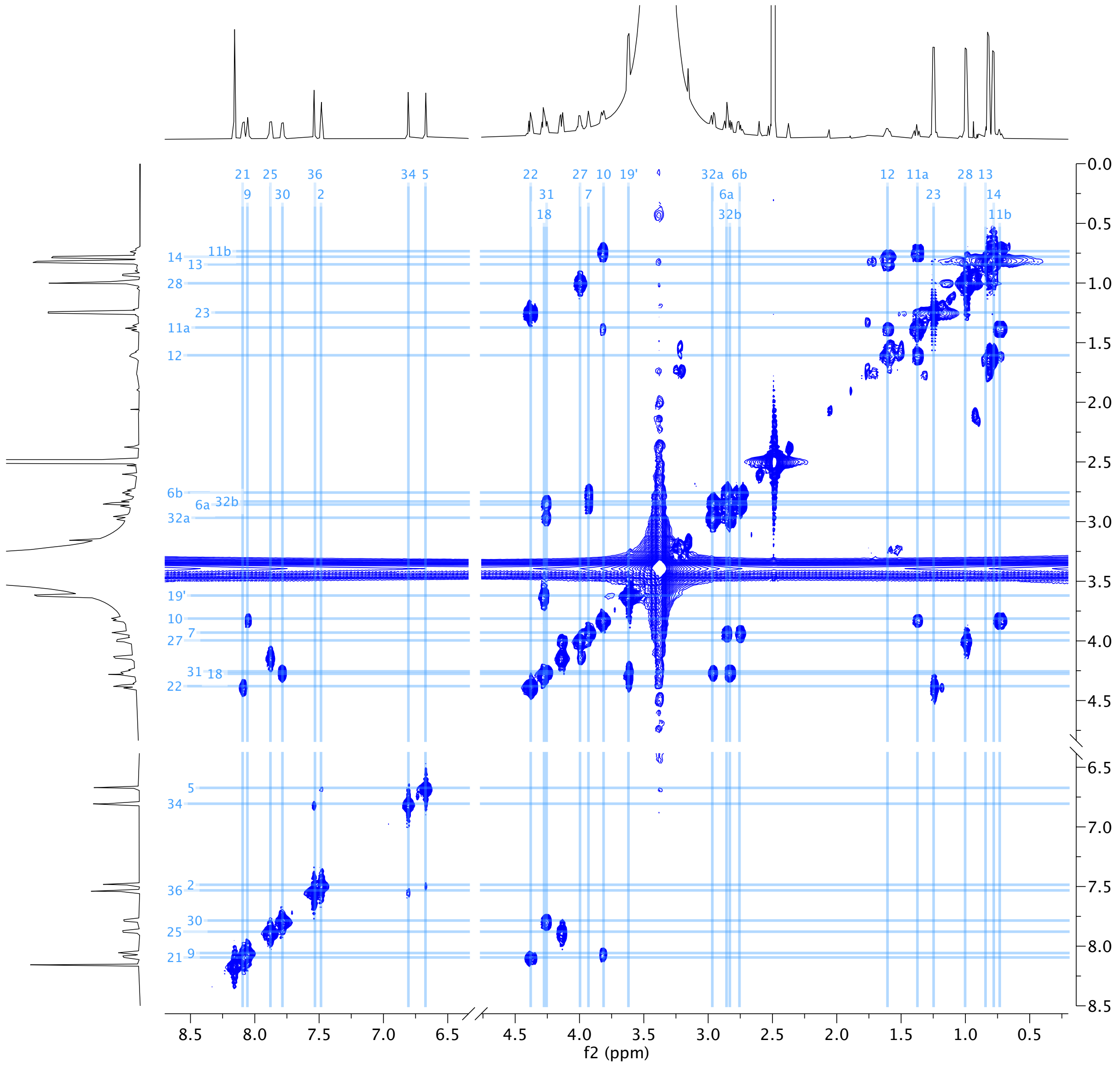

Figure S15 2D COSY spectrum (DMSO-d_6, 298 K) of streptamidine.

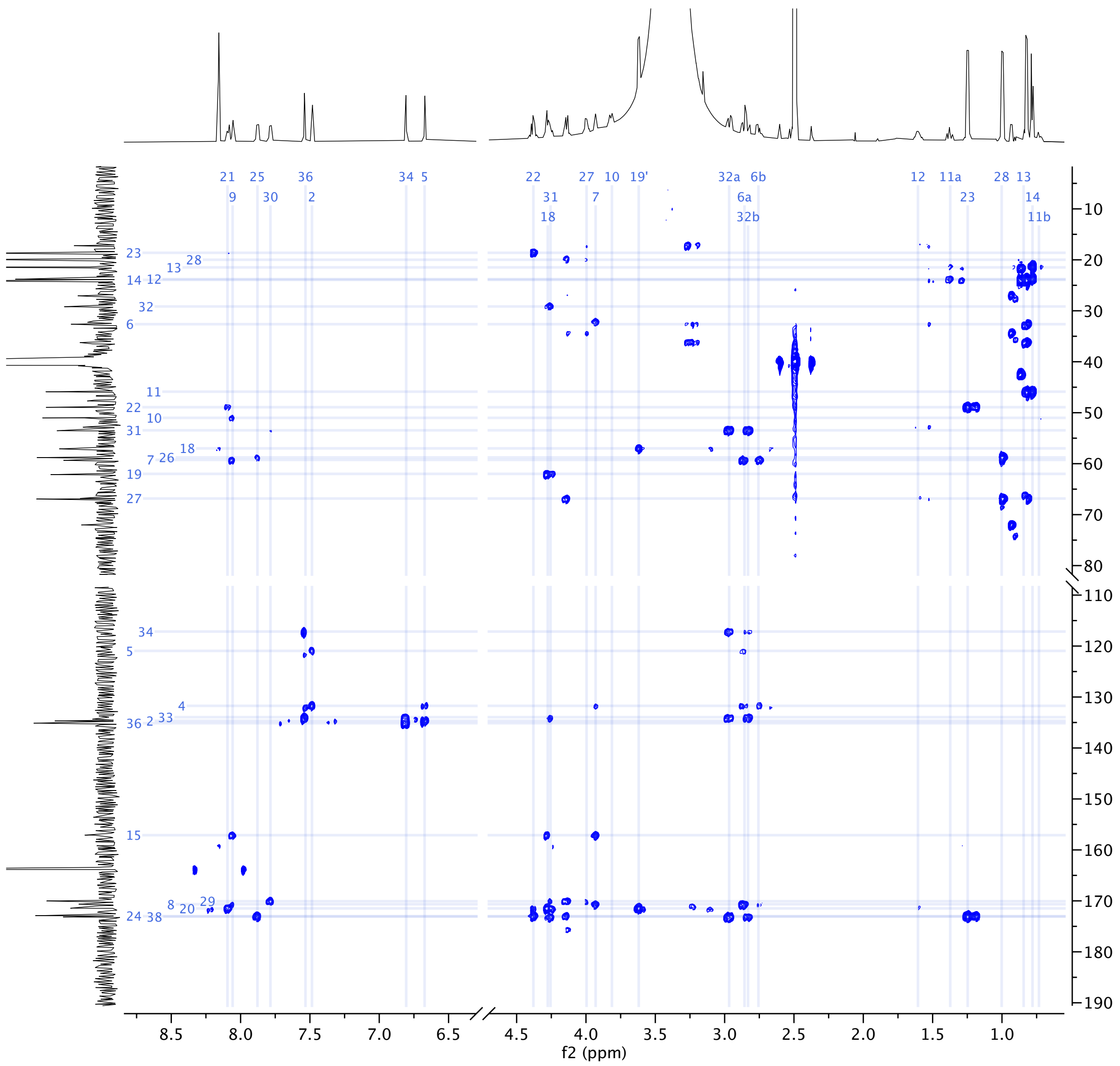

Figure S16 2D HMBC spectrum (DMSO-d_6, 298 K) of streptamidine.

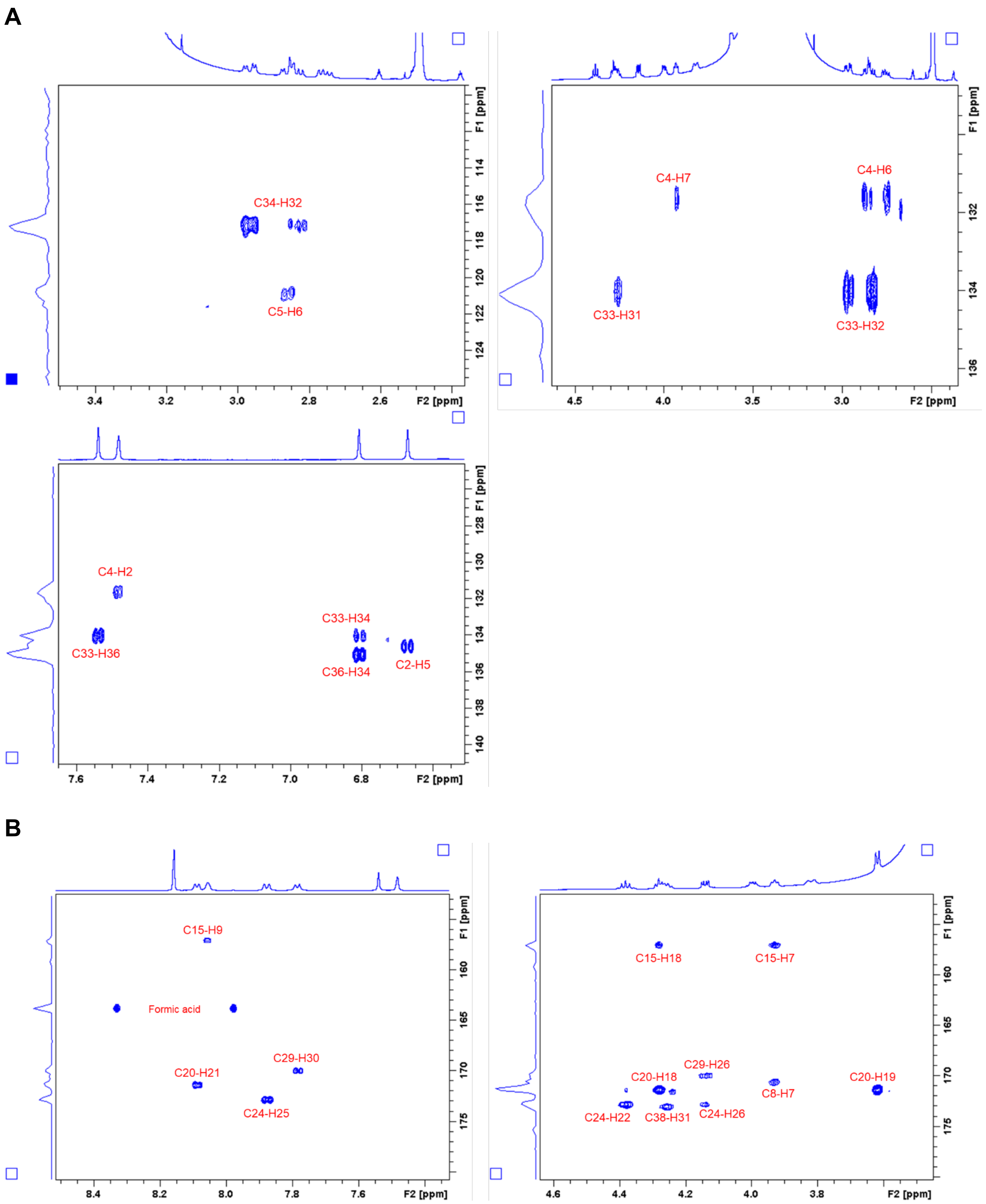

Figure S17 Selected regions of 2D HMBC spectrum. A. Key correlations for histidine residues. B. Key correlations for amidine carbon and carbonyls.

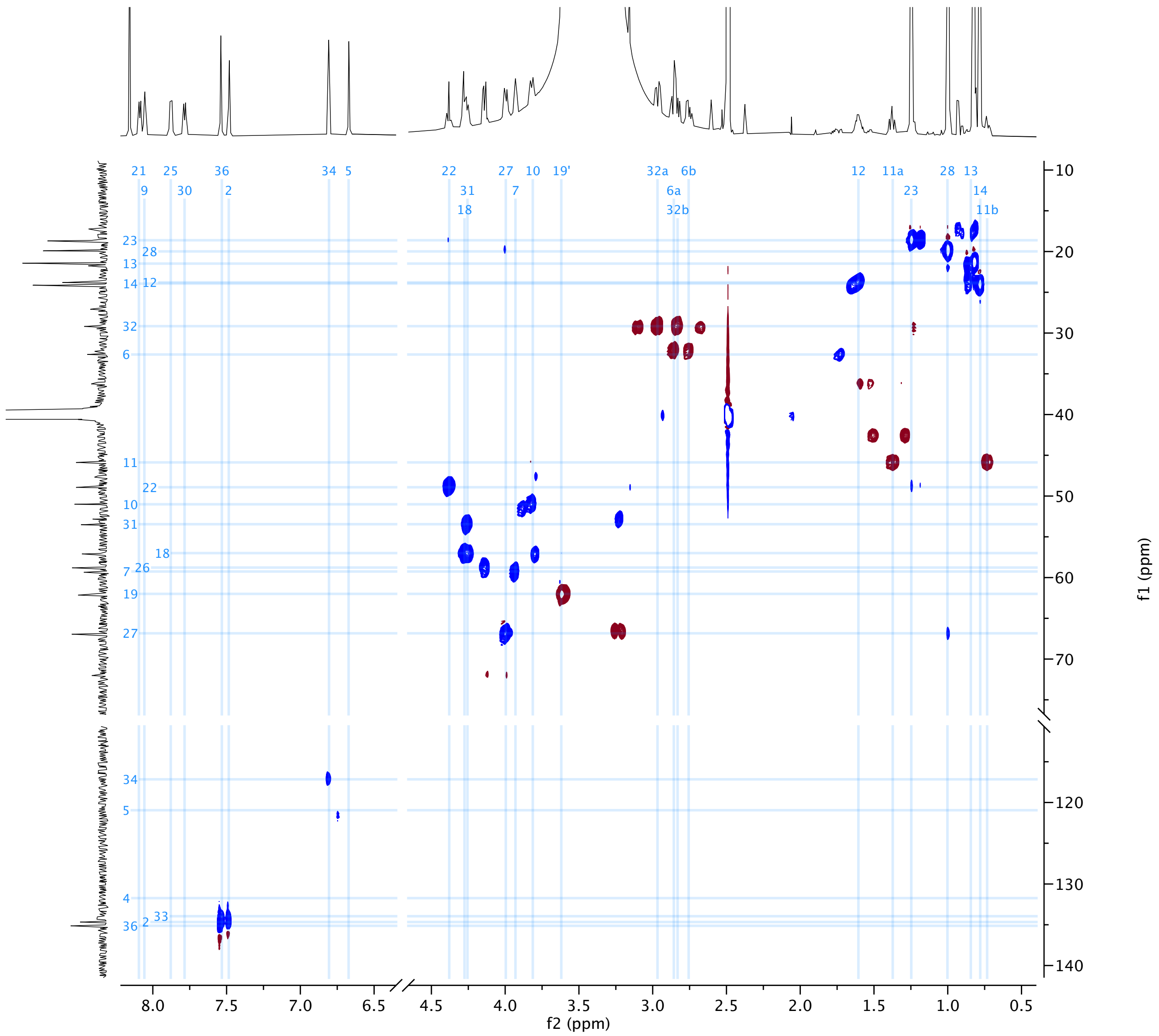

Figure S18 2D HSQCed spectrum (DMSO-d_6, 298 K) of streptamidine.

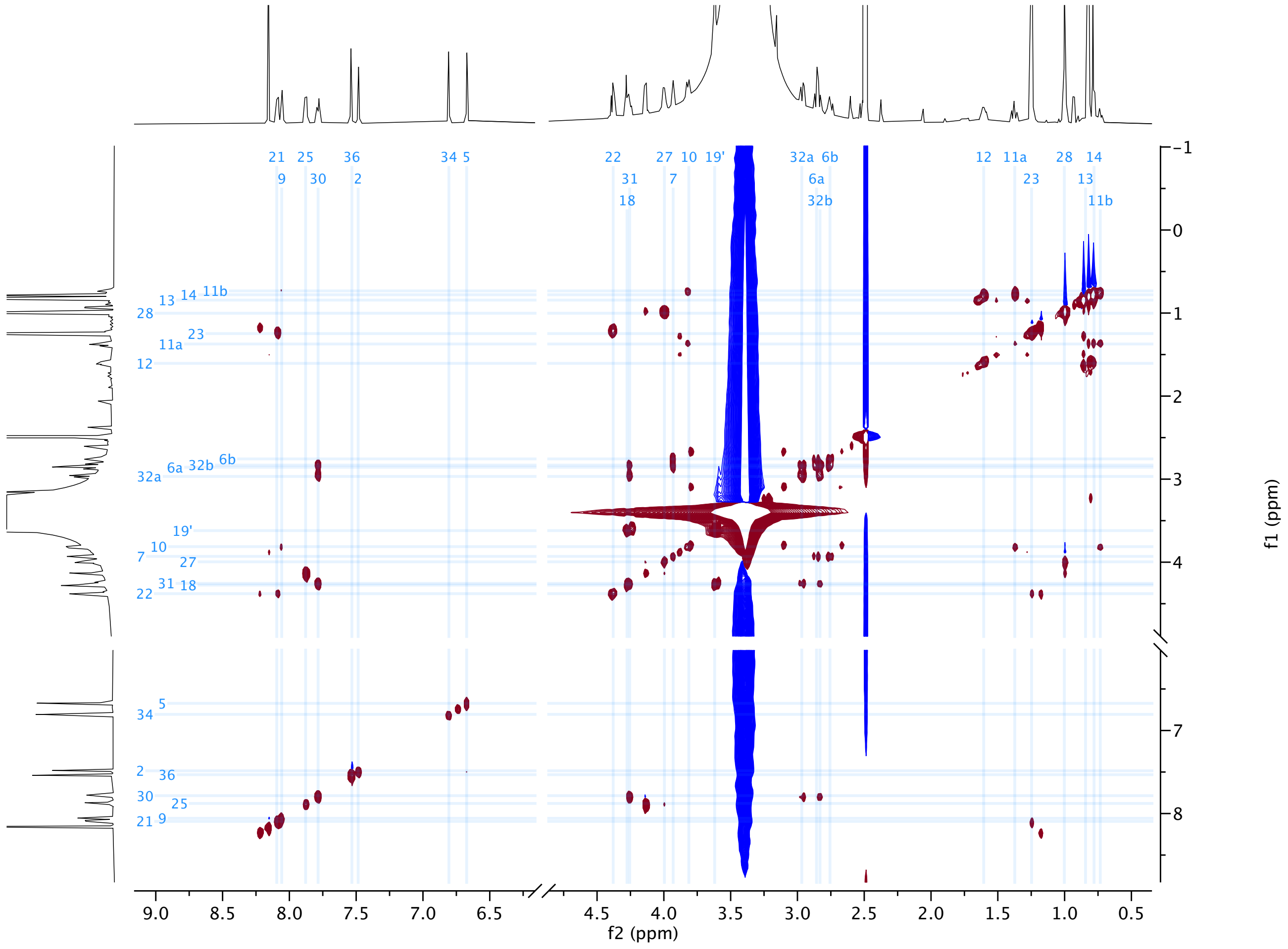

Figure S19 2D TOCSY spectrum (DMSO-d_6, 298 K) of streptamidine.

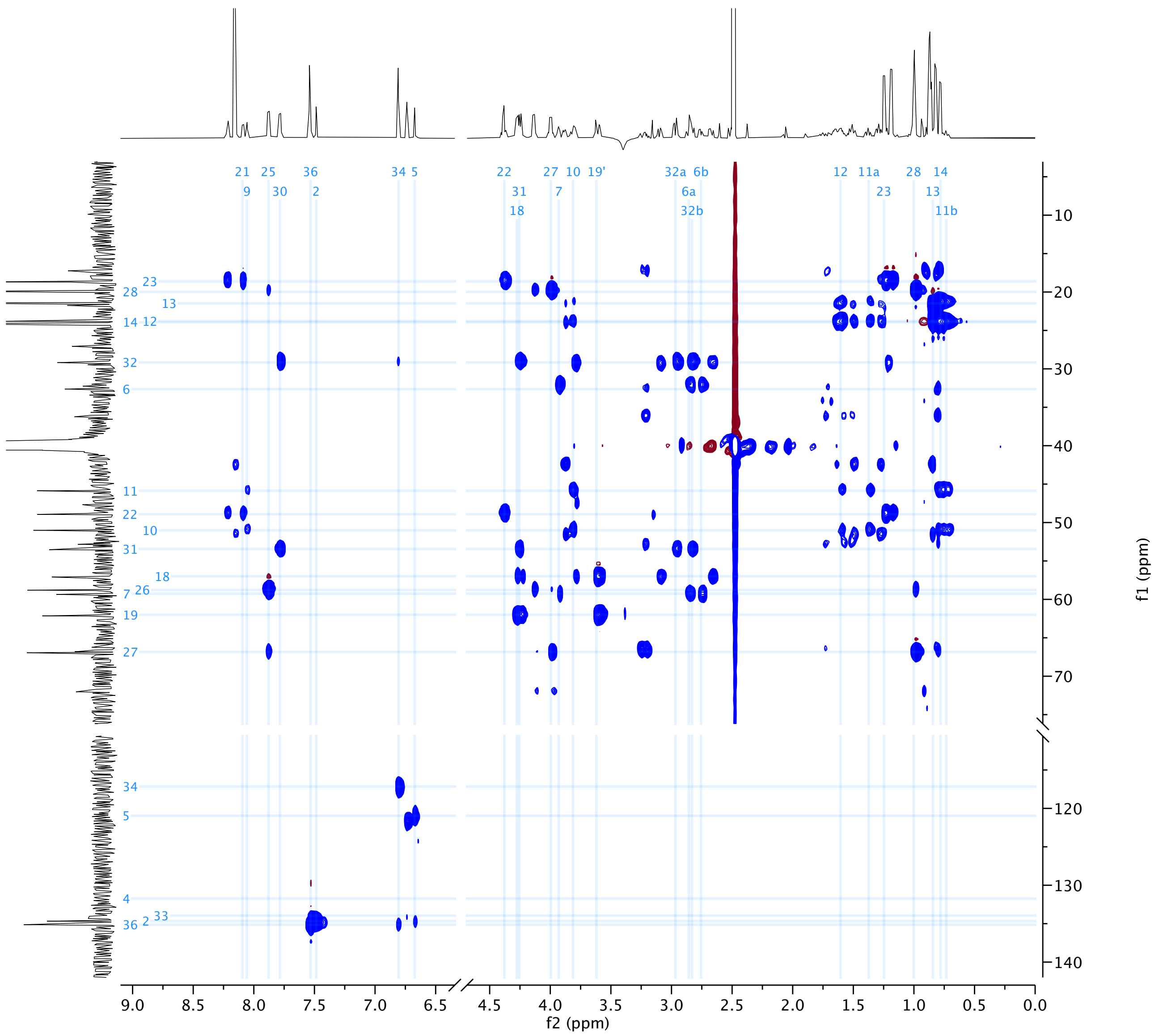

Figure S20 2D HSQC-TOCSY spectrum (DMSO-d$_6$, 298 K) of streptamidine. Some extra cross-peaks (e.g. for C5) are predicted to relate to an isomerisation during the acquisition of spectra.

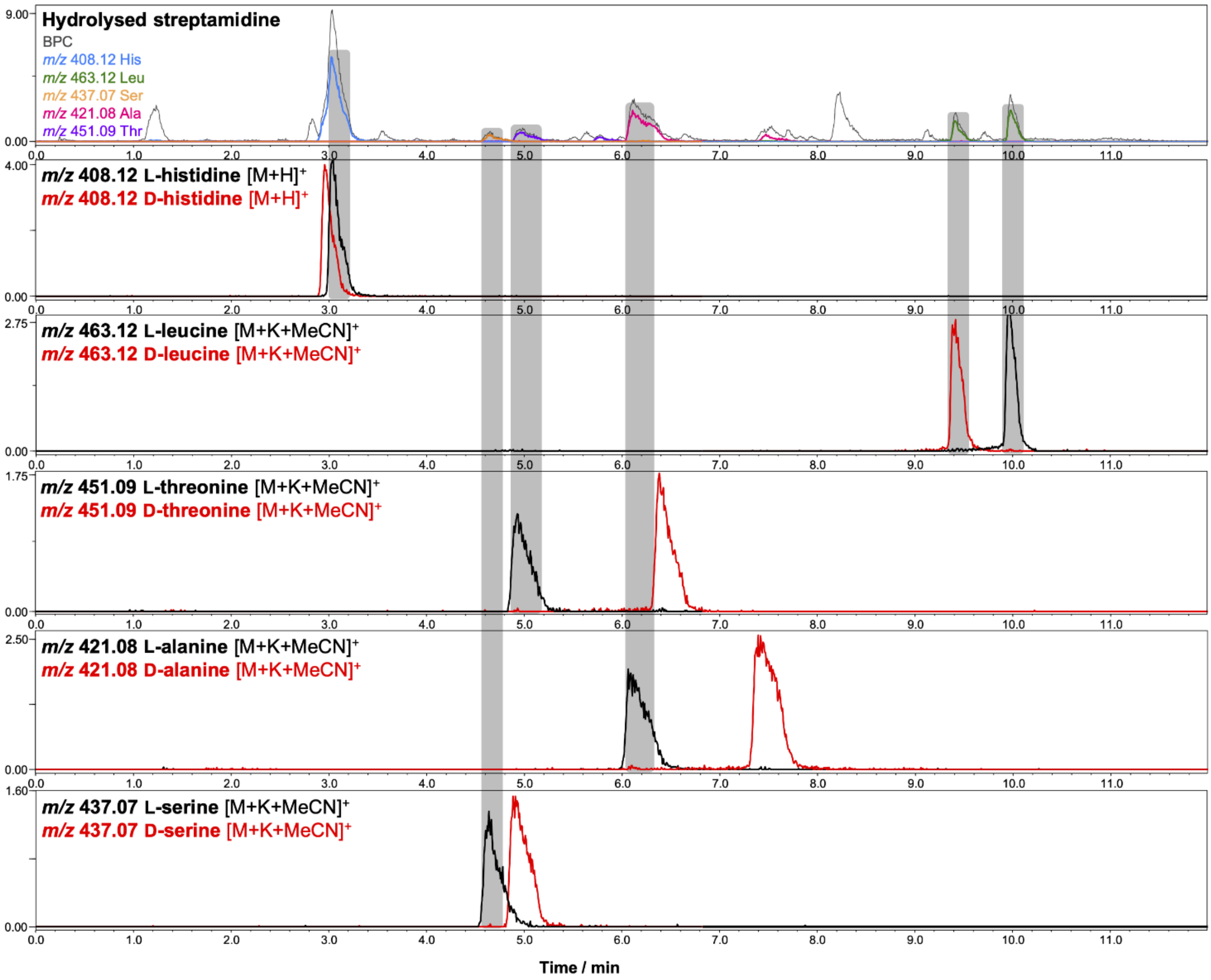

Figure S21 LC-MS chromatograms following Marfey's analysis of streptamidine. The top panel shows the hydrolysed streptamidine sample where constituent amino acids have been derivatised with Marfey's reagent. Below this are the LC-MS chromatograms obtained from derivatisation of individual amino acid standards of L- and D- amino acids. With the exception of histidine, the unusual $[M+K+MeCN]^+$ adducts were the dominant species detected in both the streptamidine sample and the amino acid standards.

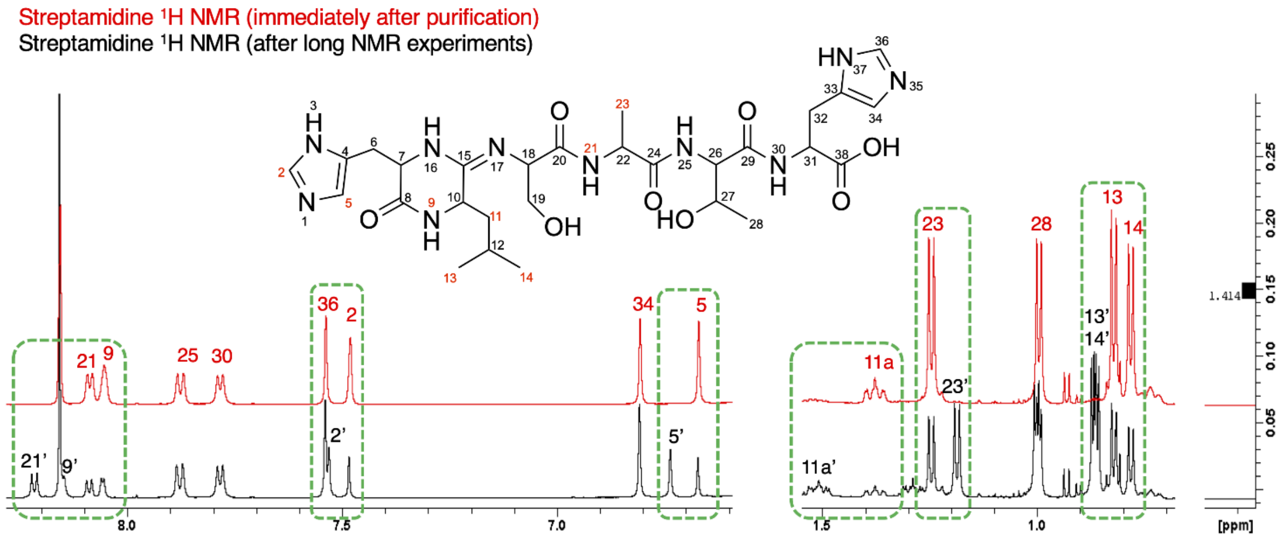

Figure S22 Streptamidine isomerisation observed via NMR. The spectrum immediately after purification is shown as a red trace and the spectrum after long NMR experiments is shown in black. Protons shifts that significantly change are highlighted in red on the structure.

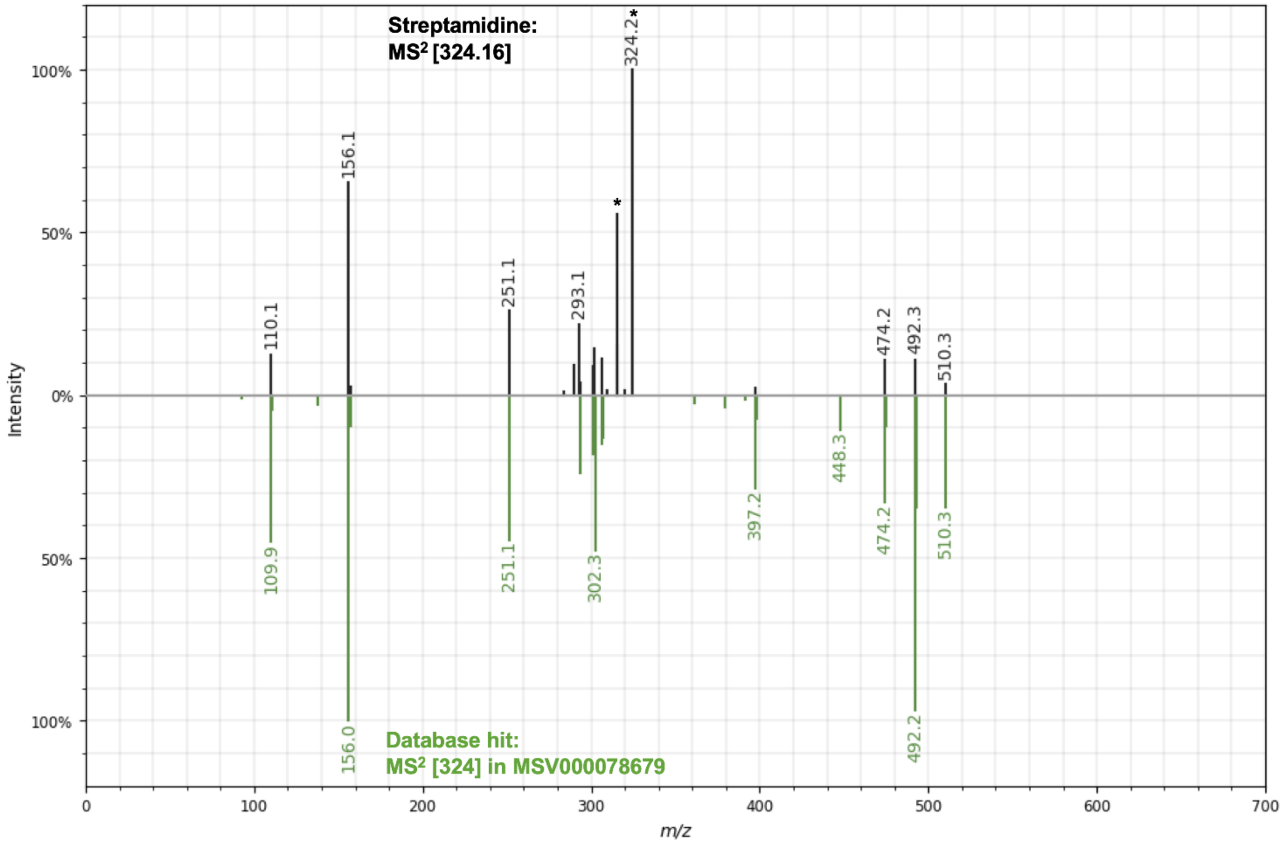

Figure S23 Spectral match to the MS/MS spectrum of [streptamidine+2H]$^{2+}$ (*m/z* 324.16) identified using MASST (Mass Spectrometry Search Tool)[23] at GNPS (Global Natural Products Social Molecular Networking). The top spectrum (black) is from streptamidine and the mirror bottom spectrum (green) is the database hit. The hit is found in multiple samples of MassIVE Dataset MSV000078679 ("Zhang lab_microbes library_MS130001~9"), which is defined as an actinomycete dataset. Asterisks indicate non-matching peaks that feature a double charge: *m/z* 324.2 (unfragmented streptamidine) and *m/z* 315.2 (dehydrated streptamidine).

Table S10 Indicator strains tested in bioactivity assays.

Organism tested	Description	Inhibition observed
E. coli ATCC25922	Gram-negative bacterium	None
E. coli NR986	Gram-negative bacterium (mutant with increased membrane permeability)[33]	None
Pseudomonas aeruginosa PA01	Gram-negative bacterium	None
Pseudomonas fluorescens	Gram-negative bacterium	None
Bacillus subtilis 168	Gram-positive bacterium	None
Micrococcus luteus	Gram-positive bacterium	None
Mycobacterium smegmatis MC2155	Gram-positive bacterium	None
Streptomyces scabies	Gram-positive bacterium	None
Streptomyces cattleya	Gram-positive bacterium	None
Candida utilis	Fungus	None

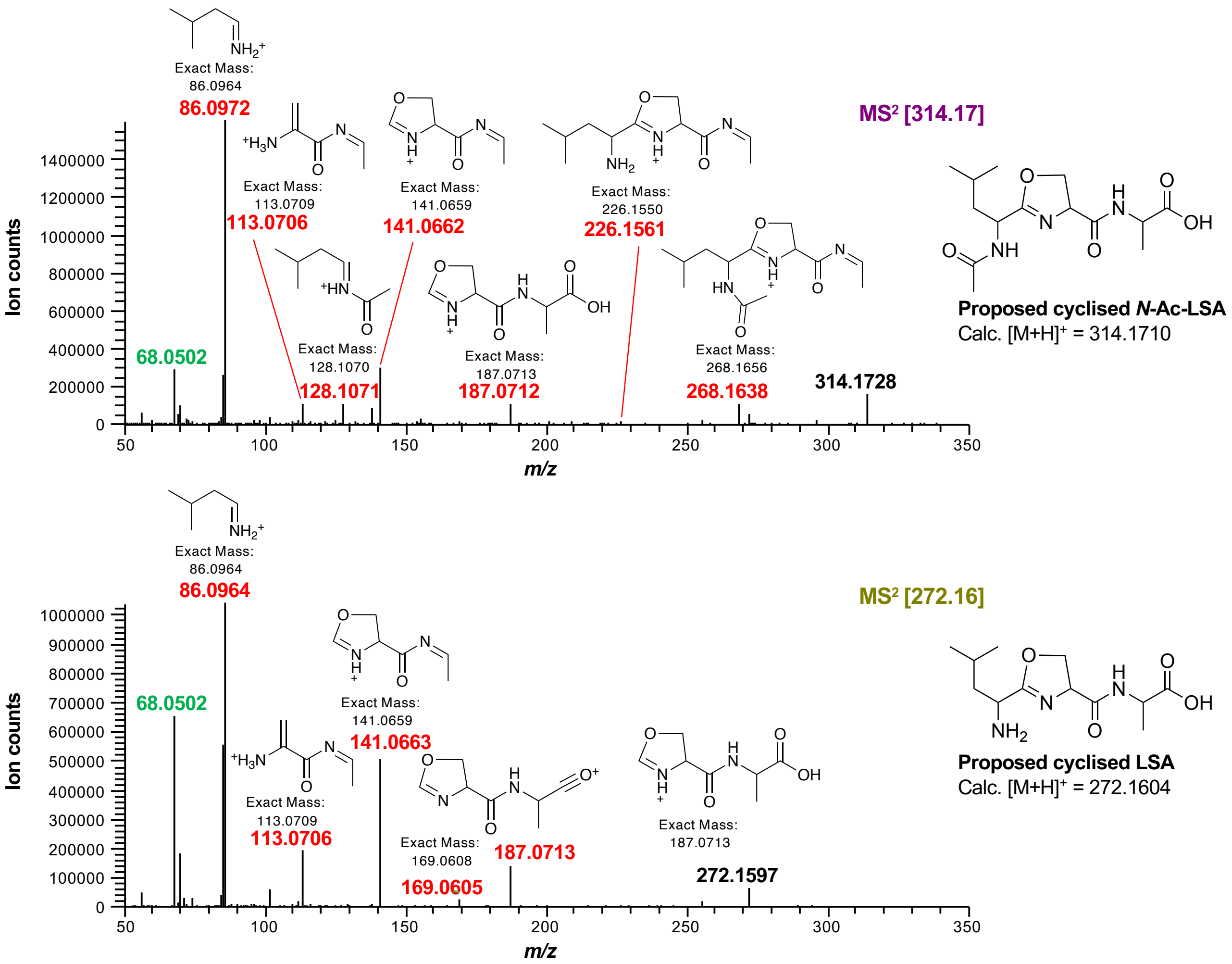

Figure S24 MS/MS fragmentation data for predicted N-acetylated and cyclised LSA (*m/z* 314.17, peptide A) and predicted cyclised LSA (*m/z* 272.16, peptide B), obtained using a Thermo Q-Exactive.

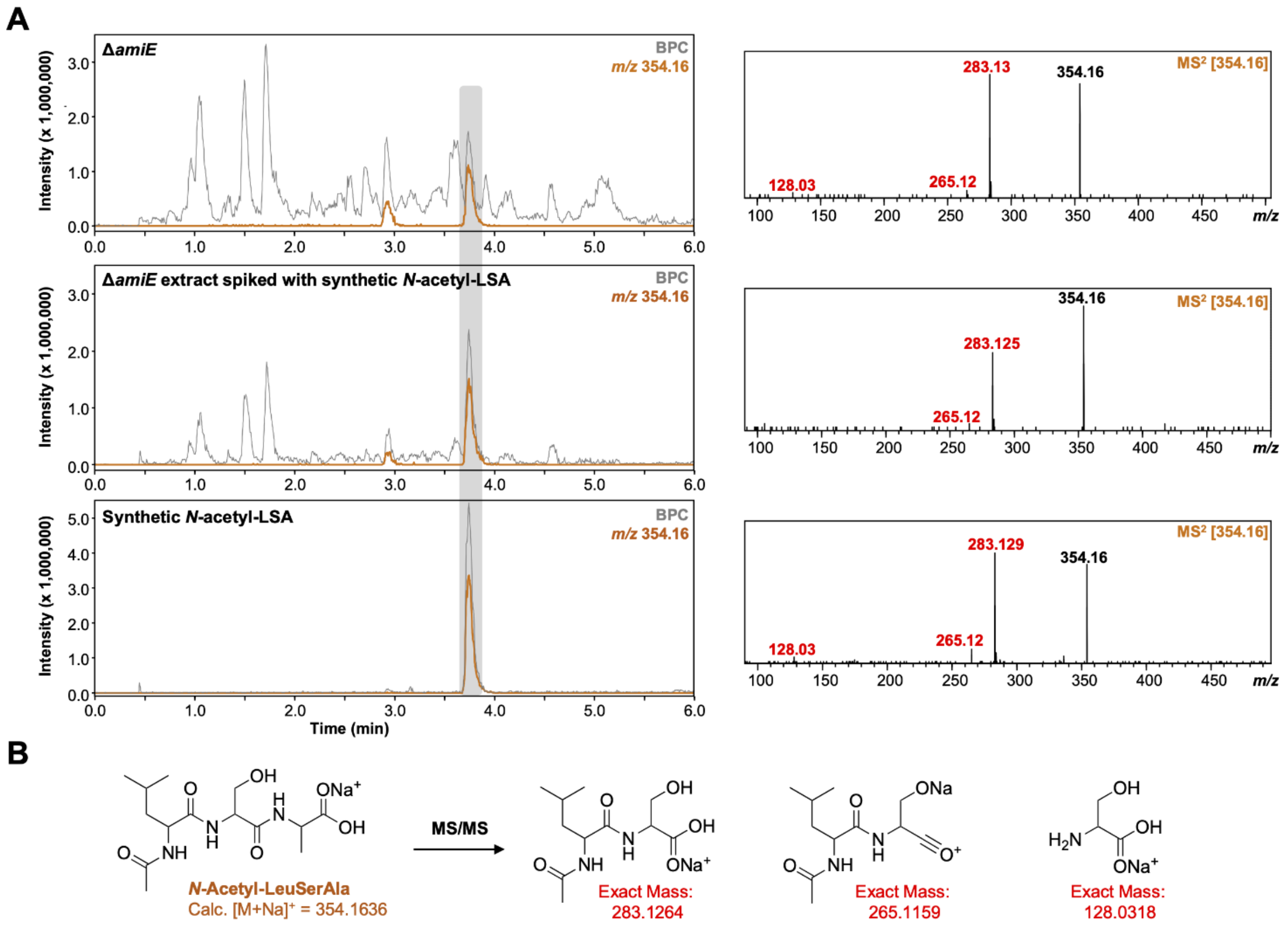

Figure S25 Identity of *m/z* 354.16 (peptide C). A. LC-MS analysis (Shimadzu IT-TOF) of *S. coelicolor* M1146-SalbC Δ*amiE* culture extract versus a synthetic standard of *N*-acetylated LSA peptide (BPC = base peak chromatogram) and a mixed sample. Extracted ion chromatogram of *m/z* 354.16 (orange) corresponds to the mass of the sodium adduct of *N*-acetylated LSA. MS/MS spectra are shown for *m/z* 354.16 in the culture extract, the spiked sample, and the synthetic standard. B. Proposed MS/MS fragmentation for *m/z* 354.16. Fragments 283.13 and 128.03 represent examples of non-canonical MS/MS fragmentation of sodiated peptides that results in the generation of carboxylated fragments instead of conventional b ions[34,35].

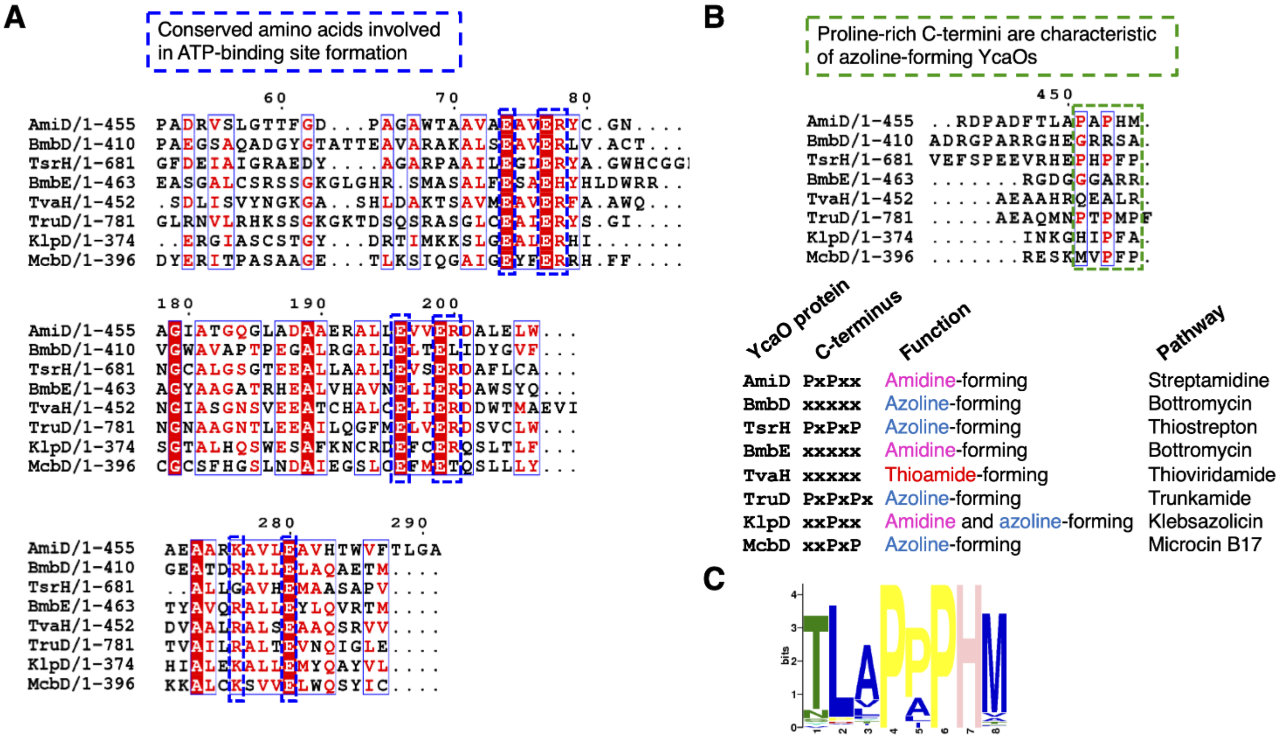

Figure S26 Multiple sequence alignment of YcaO proteins. A. Conserved ATP-binding residues across diverse YcaO proteins involved in RiPP biosynthesis. Sequences aligned using MUSCLE[5] and visualised using Espript[36]. B. Proline content of the C-termini of these proteins. C. MEME analysis[14] of the C-termini of all YcaO proteins associated with streptamidine-like (motif A) precursor peptides.

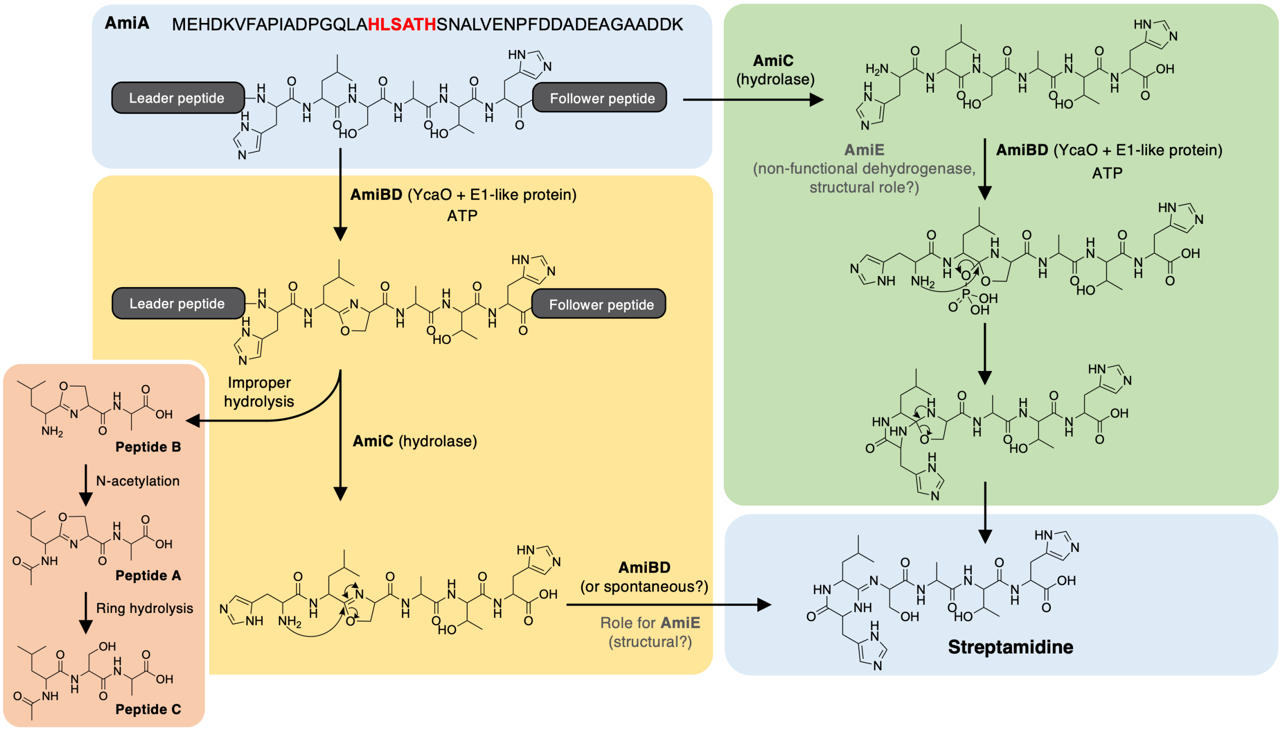

Figure S27 Proposed mechanism of amidine ring formation in streptamidine based on predicted intermediates produced by dehydrogenase mutant. The yellow box represents a route that goes via a stable oxazoline intermediate prior to leader/follower peptide removal, where AmiE potentially has a structural role within an AmiBDE complex for proper amidine formation but is not needed for initial ATP-dependent oxazoline formation. The green box represents a route where the leader/follower peptide is removed first, thereby providing a free *N*-terminal amine for cyclisation via an *O*-phosphorylated hemiorthoamide. In the absence of AmiE, the final amidine forming step could potentially be disrupted. A possible shunt route to peptides A, B and C is proposed.

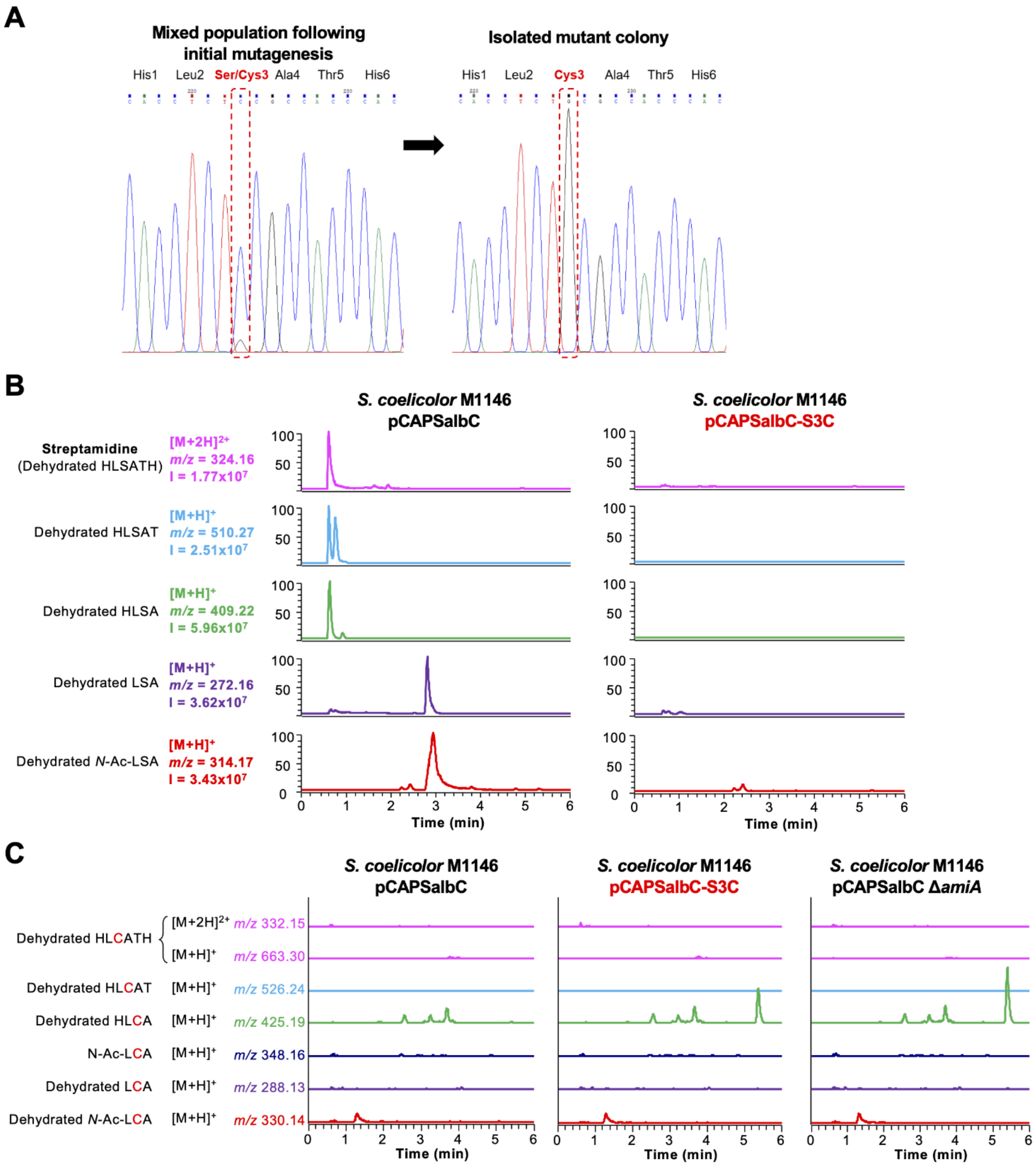

Figure S28 Site-directed mutagenesis of AmiA. A. Sequence files obtained following PCR screening of colonies following mutagenesis of pCAPSalbC. A mixed plasmid population of "wild type" and mutant pCAPSalbC is shown following transformation of *E. coli* HME68-pCAPSalbC with mutant oligonucleotide. A clean pCAPSalbC-S3C mutant was obtained following transformation of the mixed plasmid population into *E. coli* DH5α and PCR screening of single colonies. B. EICs for masses corresponding to streptamidine and other pathway shunt metabolites in culture extracts from *S. coelicolor* M1146 containing the wild-type *ami* BCG (pCAPSalbC) or the AmiA-S3C mutant version (pCAPSalbC-S3C). Chromatograms in each row are normalised to the intensity (I) indicated to their left. C. EICs for predicted masses corresponding to cysteine-containing versions of pathway metabolites in culture extracts from *S. coelicolor* M1146 containing the wild-type *ami* BCG (pCAPSalbC), the S3C mutant (pCAPSalbC-S3C) and the Δ*amiA* mutant (pCAPSalbC Δ*amiA*). All spectra obtained on a Thermo Q-Exactive.

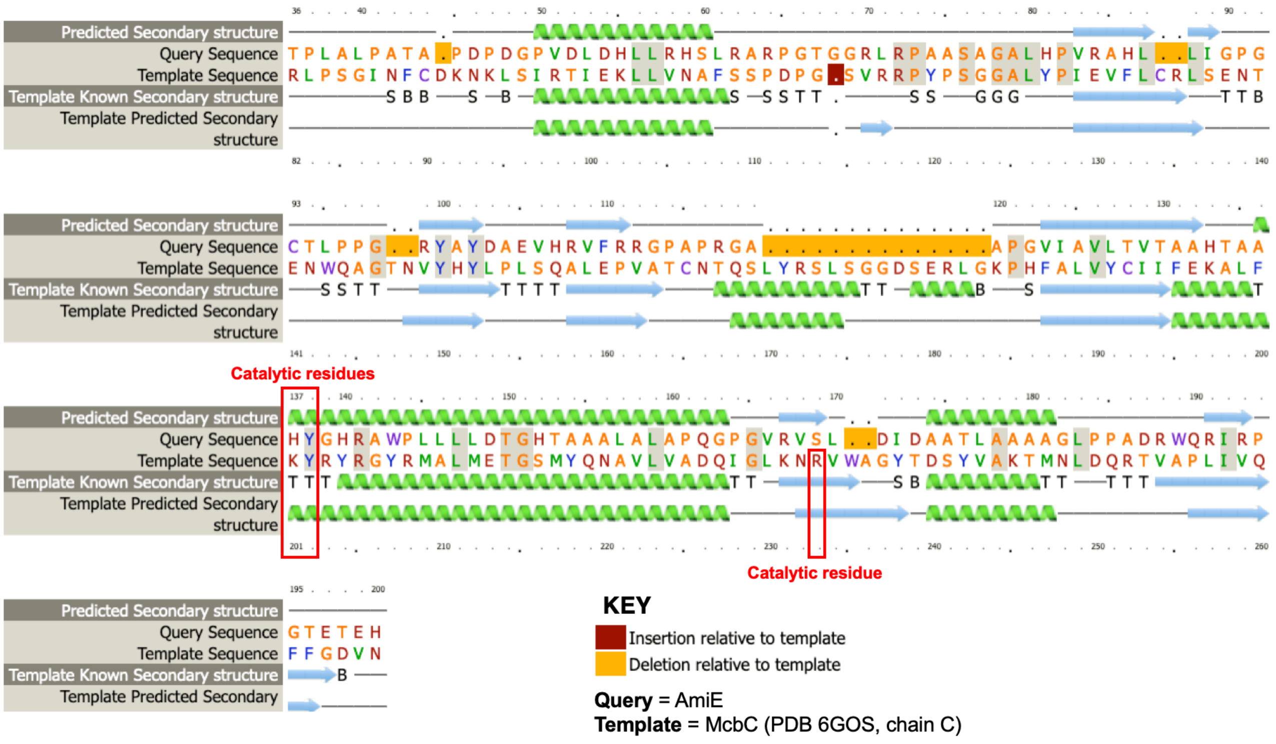

Figure S29 Secondary structure alignment of AmiE with McbC (PDB 6GOS)[37] from the microcin B17 pathway generated with Phyre2[18]. Catalytic residues identified by Melby *et al.*[38] in McbC are highlighted.

REFERENCES

1 V. Noskov, N. Kouprina, S.-H. Leem, M. Koriabine, J. C. Barrett and V. Larionov, *Nucleic Acids Res.*, 2002, **30**, E8.

2 B. Gust, G. L. Challis, K. Fowler, T. Kieser and K. F. Chater, *Proc. Natl. Acad. Sci. U.S.A.*, 2003, **100**, 1541–1546.

3 L. Y. Geer, M. Domrachev, D. J. Lipman and S. H. Bryant, *Genome Res.*, 2002, **12**, 1619–1623.

4 R. Zallot, N. Oberg and J. A. Gerlt, *Biochemistry*, 2019, **58**, 4169–4182.

5 R. C. Edgar, *Nucleic Acids Res.*, 2004, **32**, 1792–1797.

6 A. Stamatakis, *Bioinformatics*, 2014, **30**, 1312–1313.

7 I. Letunic and P. Bork, *Nucleic Acids Res.*, 2016, **44**, W242–W245.

8 J. Santos-Aberturas, G. Chandra, L. Frattaruolo, R. Lacret, T. H. Pham, N. M. Vior, T. H. Eyles and A. W. Truman, *Nucleic Acids Res.*, 2019, **47**, 4624–4637.

9 A. D. Moffat, J. Santos-Aberturas, G. Chandra and A. W. Truman, *Methods Mol. Biol.*, 2021, **2296**, 227–247.

10 S. Halary, J. O. McInerney, P. Lopez and E. Bapteste, *BMC Evol. Biol.*, 2013, **13**, 146.

11 P. Shannon, A. Markiel, O. Ozier, N. S. Baliga, J. T. Wang, D. Ramage, N. Amin, B. Schwikowski and T. Ideker, *Genome Res.*, 2003, **13**, 2498–2504.

12 J. D. Thompson, D. G. Higgins and T. J. Gibson, *Nucleic Acids Res.*, 1994, **22**, 4673–4680.

13 S. Kumar, G. Stecher and K. Tamura, *Mol. Biol. Evol.*, 2016, **33**, 1870–1874.

14 T. L. Bailey, M. Boden, F. A. Buske, M. Frith, C. E. Grant, L. Clementi, J. Ren, W. W. Li and W. S. Noble, *Nucleic Acids Res.*, 2009, **37**, W202–W208.

15 T. Carver, S. R. Harris, M. Berriman, J. Parkhill and J. A. McQuillan, *Bioinformatics*, 2012, **28**, 464–469.

16 G. Lu and E. N. Moriyama, *Brief Bioinform.*, 2004, **5**, 378–388.

17 M. H. Medema, E. Takano and R. Breitling, *Mol. Biol. Evol.*, 2013, **30**, 1218–1223.

18 L. A. Kelley, S. Mezulis, C. M. Yates, M. N. Wass and M. J. E. Sternberg, *Nat. Protoc.*, 2015, **10**, 845–858.

19 E. L. C. de Los Santos, *Sci. Rep.*, 2019, **9**, 13406.

20 X. Tang, J. Li, N. Millán-Aguiñaga, J. J. Zhang, E. C. O'Neill, J. A. Ugalde, P. R. Jensen, S. M. Mantovani and B. S. Moore, *ACS Chem. Biol.*, 2015, **10**, 2841–2849.

21 S. A. Kautsar, K. Blin, S. Shaw, J. C. Navarro-Muñoz, B. R. Terlouw, J. J. J. van der Hooft, J. A. van Santen, V. Tracanna, H. G. Suarez Duran, V. Pascal Andreu, N. Selem-Mojica, M. Alanjary, S. L. Robinson, G. Lund, S. C. Epstein, A. C. Sisto, L. K. Charkoudian, J. Collemare, R. G. Linington, T. Weber and M. H. Medema, *Nucleic Acids Res.*, 2020, **48**, D454–D458.

22 N. Costantino and D. L. Court, *Proc. Natl. Acad. Sci. U.S.A.*, 2003, **100**, 15748–15753.

23 M. Wang, A. K. Jarmusch, F. Vargas, A. A. Aksenov, J. M. Gauglitz, K. Weldon, D. Petras, R. da Silva, R. Quinn, A. V. Melnik, J. J. J. van der Hooft, A. M. Caraballo-Rodríguez, L.-F. Nothias, C. M. Aceves, M. Panitchpakdi, E. Brown, F. Di Ottavio, N. Sikora, E. O. Elijah, L. Labarta-Bajo, E. C. Gentry, S. Shalapour, K. E. Kyle, S. P. Puckett, J. D. Watrous, C. S. Carpenter, A. Bouslimani, M. Ernst, A. D. Swafford, E. I. Zuniga, M. J. Balunas, J. L. Klassen, R. Loomba, R. Knight, N. Bandeira and P. C. Dorrestein, *Nat. Biotechnol.*, 2020, **38**, 23–26.
24 D. B. Alexander and D. A. Zuberer, *Biol. Fert Soils*, 1991, **12**, 39–45.
25 G. Müller and K. N. Raymond, *J. Bacteriol.*, 1984, **160**, 304–312.
26 K. F. Chater and L. C. Wilde, *J. Gen. Microbiol.*, 1980, **116**, 323–334.
27 J. P. Gomez-Escribano and M. J. Bibb, *Microb. Biotechnol.*, 2011, **4**, 207–215.
28 M. Bierman, R. Logan, K. O'Brien, E. T. Seno, R. N. Rao and B. E. Schoner, *Gene*, 1992, **116**, 43–49.
29 H.-J. Hong, M. I. Hutchings, L. M. Hill and M. J. Buttner, *J. Biol. Chem.*, 2005, **280**, 13055–13061.
30 A. M. Waterhouse, J. B. Procter, D. M. A. Martin, M. Clamp and G. J. Barton, *Bioinformatics*, 2009, **25**, 1189–1191.
31 W. R. Taylor, *Protein Eng.*, 1997, **10**, 743–746.
32 V. Prabhu, B. Chatson, G. Abrams and J. King, *J. Plant Physiol.*, 1996, **149**, 246–250.
33 J. E. Button, T. J. Silhavy and N. Ruiz, *J. Bacteriol.*, 2007, **189**, 1523–1530.
34 R. P. Grese, R. L. Cerny and M. L. Gross, *J. Am. Chem. Soc.*, 1989, **111**, 2835–2842.
35 K. A. Newton and S. A. McLuckey, *J. Am. Soc. Mass Spectrom.*, 2004, **15**, 607–615.
36 X. Robert and P. Gouet, *Nucleic Acids Res.*, 2014, **42**, W320–W324.
37 D. Ghilarov, C. E. M. Stevenson, D. Y. Travin, J. Piskunova, M. Serebryakova, A. Maxwell, D. M. Lawson and K. Severinov, *Mol. Cell*, 2019, **73**, 749-762.e5.
38 J. O. Melby, X. Li and D. A. Mitchell, *Biochemistry*, 2014, **53**, 413–422.
